# GC–MS-Based Nontargeted and Targeted Metabolic Profiling Identifies Changes in the *Lentinula edodes* Mycelial Metabolome under High-Temperature Stress

**DOI:** 10.3390/ijms20092330

**Published:** 2019-05-10

**Authors:** Xu Zhao, Mingjie Chen, Yan Zhao, Lei Zha, Huanling Yang, Yuejin Wu

**Affiliations:** 1 Institute of Technical BiologyAgriculture Engineering, Hefei Institutes of Physical Science, Chinese Academy of Sciences, Hefei 230031, China; zhaoxu512@163.com; 2 University of Science and Technology of China, Hefei 230026, China; 3 Institute of Edible Fungi, Shanghai Academy of Agricultural Sciences, Shanghai 201403, China; mjchen@saas.sh.cn (M.C.); zhalei@saas.sh.cn (L.Z.); yanghuanling@saas.sh.cn (H.Y.)

**Keywords:** *Lentinula edodes*, high temperature stress, GC-MS, metabolomics

## Abstract

To clarify the physiological mechanism of the *Lentinula edodes* (*L. edodes*) response to high-temperature stress, two strains of *L. edodes* with different tolerances were tested at different durations of high temperature, and the results showed that there were significant changes in their phenotypes and physiology. To further explore the response mechanism, we established a targeted GC–MS-based metabolomics workflow comprising a standardized experimental setup for growth, treatment and sampling of *L. edodes* mycelia, and subsequent GC–MS analysis followed by data processing and evaluation of quality control (QC) measures using tailored statistical and bioinformatic tools. This study identified changes in the *L. edodes* mycelial metabolome following different time treatments at high temperature based on nontargeted metabolites with GC-MS and further adopted targeted metabolomics to verify the results of the analysis. After multiple statistical analyses were carried out using SIMCA software, 74 and 108 differential metabolites were obtained, respectively. Kyoto Encyclopedia of Genes and Genomes (KEGG) analysis showed that the metabolic pathways with significant changes included those related to the following: amino acid metabolism, the glycolysis pathway, the tricarboxylic acid (TCA) cycle, and sugar metabolism. Most amino acids and carbohydrates enriched in these metabolic pathways were upregulated in strain 18, downregulated in strain 18N44, or the synthesis in strain 18 was higher than that in strain 18N44. This result was consistent with the physiological phenotypic characteristics of the two strains under high-temperature stress and revealed the reason why strain 18N44 was more heat-sensitive. At the same time, under high temperature, the decrease of intermediate products in glycolysis and the TCA cycle resulted in carbon starvation and insufficient energy metabolism, thus inhibiting the growth of *L. edodes*. In addition, the results also showed that the metabolites produced by different *L. edodes* strains under high-temperature stress were basically the same. However, different strains had species specificity, so the changes in the content of metabolites involved in the response to high-temperature stress were different. This provides a theoretical basis for further understanding the mechanism of the *L. edodes* response to high temperature and can be used to establish an evaluation system of high-temperature-resistant strains and lay the foundation for molecular breeding of new *L. edodes* strains resistant to high temperature.

## 1. Introduction

*Lentinula edodes* (*L. edodes*), also called dried mushrooms, are rich in nutrition, have a delicious taste and a variety of health functions, and are known as the Queen of Mushrooms [1,2]. This species is the second largest edible fungus in the world and one of the major edible fungi produced on a large scale in China [3]. *L. edodes* is a medium- and low-temperature fructifying mushroom. The optimum temperature for the growth of *L. edodes* mycelia is 24–27 °C, and it will die at 38 °C and above [4]. However, in actual production, the summer temperature in the main production area of *L. edodes* is much higher than 25 °C. This high temperature seriously damages *L. edodes*. A “fungus burning phenomenon” occurs at high temperature, and the mycelium becomes yellow or even stops growing, which seriously affects the yield of *L. edodes* [5]. Therefore, it is of great significance to study the physiological mechanism of resistance to high-temperature stress in *L. edodes*.

Metabonomics is an emerging branch of systems biology following genomics, transcriptomes, and proteomics [6], and was proposed by Nicholson et al. in 1999 [7]. That is, all metabolites of a certain biological component or cell are analyzed qualitatively and quantitatively at the same time in a certain physiological period or condition to discover differential metabolites and then to clarify the complete physiological state of the biological component or cell at a certain time. Currently, metabonomics is mainly divided into nontargeted metabonomics and targeted metabonomics. Nontargeted metabonomics is an analytical strategy for detecting metabolites in as many samples as possible, which can guide the resolution of biological problems at the metabolic level, generally without bias. Targeted metabonomics is a metabolic analysis strategy that considers a specific number or type of metabolite, with a subjective purpose. The main analytical techniques used for metabonomics include gas chromatography (GC), liquid chromatography (LC), mass spectrometry (MS), and nuclear magnetic resonance (NMR) [8]. According to the difference in the chromatographic mobile phase, techniques can be divided into gas chromatography–mass spectrometry (GC–MS) and liquid chromatography–mass spectrometry (LC–MS). Metabonomic studies during microbial abiotic stress have been reported. Benedikt Warth et al. [9] analyzed by GC–MS the metabolomic changes of wheat treated with deoxynivalenol (DON). The metabolism and transport of primary carbohydrates, the tricarboxylic acid cycle, and primary nitrogen metabolism were significantly affected in DON-treated samples. Xie Huali et al. [10] studied the effects of temperature on the physiological metabolism of *Aspergillus flavus* by a nontargeted metabonomics method based on ultrahigh efficiency liquid chromatography–high resolution mass spectrometry. The study results showed that temperature can significantly affect the biosynthetic pathways of the tricarboxylic acid cycle, fatty acids, phenylalanine, tryptophan, and tyrosine and regulate the activity of biosynthetic pathways of secondary metabolites, such as aflatoxins, pinic acid, and kojic acid. Zhou Lianyu et al. [11] studied the GC–MS-based metabolomics of fescue–endophyte fungi symbiosis under low-temperature stress. The biomarkers obtained for an endophytic fungus, *Epichloe* XH 03, with low-temperature adaptability included glutamic acid, aspartic acid, stearic acid, fructose, glucose, mannitol, rhamnose, and other substances. Zhou Jia et al. [12] analyzed by GC–MS the metabolic changes of wild-type and *Bacillus thuringiensis* (*Bt*) transgenic rice after pesticide treatment. The results show that *Bt* transgenic rice has a more intense response than wild-type rice in the antioxidant system and signal regulation.

Metabonomics has been introduced into the study of edible fungi in recent years and has attracted wide attention [13]. *L. edodes*, as one of the edible fungi, has wide-ranging edible and medicinal value. Therefore, most studies focus on quality improvement and yield improvement [14,15,16,17,18,19,20,21], but there are few studies on *L. edodes* under adversity stress. High temperature is a practical problem commonly faced in the production of *L. edodes*. However, there is no report on the analysis of high-temperature stress in *L. edodes* based on advanced metabolomic methods. Therefore, this paper aims to explore the changes in the phenotype and physiological traits of *L. edodes* stimulated by high temperatures. Then, this paper analyzes the changes in endogenous metabolites of *L. edodes* mycelium under high-temperature stress at different times by GC–MS and provides a targeted quantitative verification of key differential metabolites to evaluate the differences in stress response between the starting strain and a mutant strain and to explore the key metabolic pathways involved in the response to high temperature. This study provides a theoretical basis for further understanding the mechanism of the *L. edodes* response to high temperature, which can be used to establish an evaluation system of high-temperature-resistant strains and lay a foundation for molecular breeding of new high-temperature-resistant strains of *L. edodes*.

## 2. Results

### 2.1. Physiological Study of Different Strains of *L. edodes* in Response to High-Temperature Stress

**Figure 1:**
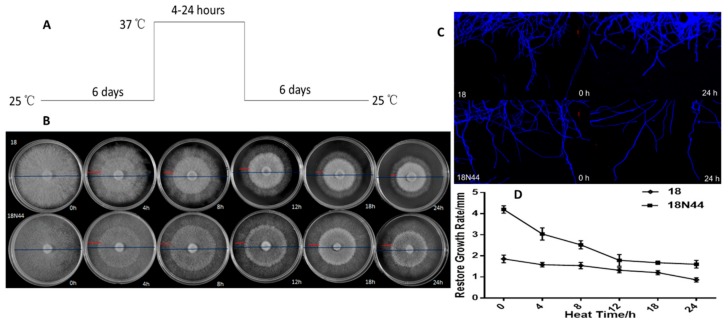
Heat stress inhibited the growth of *Lentinula edodes* mycelia. (**A**) Two strains of *L. edodes* were cultured on PDA plates for 6 d and then exposed to 37 °C for 0 to 24 h followed by 6 days at 25 °C. (**B**) The morphology of mycelial growth for the entire 13 days (blue line) and the following 6 days at 25 °C (after heat stress) (red line) is shown. (**C**) Microscopically, the mycelial morphology changed after 24 h of heat stress. Vegetative hyphae were removed from actively growing colonies, suspended in Fluorescent Brightener 28, and detected under a microscope. (**D**) Recovery of the mycelial growth rate of *L. edodes* after heat stress. The values are the means ± SD of three independent experiments.

To investigate the effects of heat stress on the growth of *L. edodes*, strain 18 and strain 18N44 were cultured normally at 25 °C for 6 d and then subjected to heat stress at 37 °C for 0–24 h. Finally, they were placed at 25 °C for 6 d of normal culture (Figure 1A). The mycelial morphology of *L. edodes* under heat stress is shown in Figure 1B. The blue line represents the mycelial morphology of *L. edodes* during the whole growth cycle, namely, during culture for 13 d; the red line represents the mycelial morphology of *L. edodes* during normal growth for 6 days at 25 °C after being treated at 37 °C. The mycelia became sparse after once being dense, and growth was inhibited after heat shock, forming an obvious heat stress circle. Figure 1C shows that, after being treated under heat stress for 24 h, the mycelial branches of *L. edodes* decreased significantly. The growth rate of mycelia after heat shock is shown in Figure 1D. With the increase of heat stress duration, the recovery of the mycelial growth rate at 25 °C became slower. The growth rate of strain 18 under heat shock at 37 °C for 24 h was 0.81 mm/d, and the growth rate of strain 18N44 under heat shock at 37 °C for 24 h was 1.613 mm/d. The growth rate of both strains was inhibited. The recovered growth rates of strain 18N44 under 0–8 h of heat shock were significantly different, and, with the increase in heat shock duration, there was no significant change in the recovered growth rate following 12–24 h of heat stress. This shows that strain 18N44 is more sensitive to heat shock treatment. However, as heat shock duration increased to 12–24 h, mycelial germination lasted for a longer time, while the effects on the recovered growth rate were not significant. With the increase of heat shock duration, the recovered growth rate of strain 18 slowed significantly.

**Figure 2:**
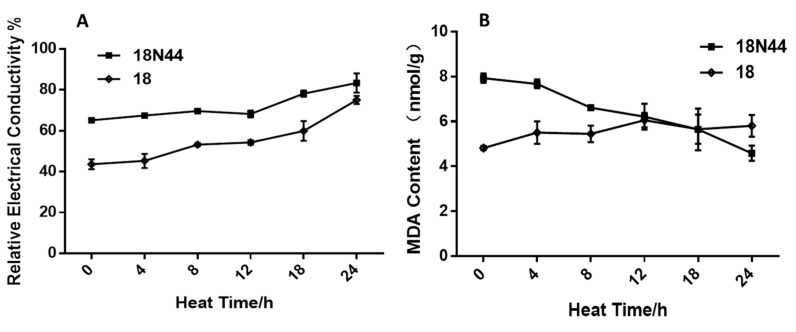
Determination of physiological indices of *L. edodes* mycelium under high stress. (**A**) Change in the mycelial conductivity of *L. edodes* under high stress. (**B**) Changes in the malondialdehyde (MDA) content of *L. edodes* after heat stress. The values are the means ± SD of three independent experiments.

The changes in the conductivity are shown in Figure 2A. Under heat shock treatment for 0 h, the relative conductivity of strain 18N44 was significantly higher than that of strain 18. By increasing the temperature stress time, the relative conductivity of mycelial cells of *L. edodes* strains 18 and 18N44 increased gradually. Under heat shock treatment for 12–24 h, the relative conductivity of strain 18 and strain 18N44 increased the most, reaching 75.1% and 83.3%, respectively.

The changes in malondialdehyde (MDA) contents are shown in Figure 2B. Under high-temperature stress, the MDA content in cells of *L. edodes* strain 18 showed a gradually increasing trend with the increase of high-temperature stress duration. At 24 h, the content was the highest. Under high-temperature stress, the MDA content in cells of *L. edodes* strain 18N44 showed a gradually decreasing trend with increasing high-temperature stress duration.

### 2.2. GC/MS Detection Results and Confirmation by Nontargeted Metabonomics

**Figure 3:**
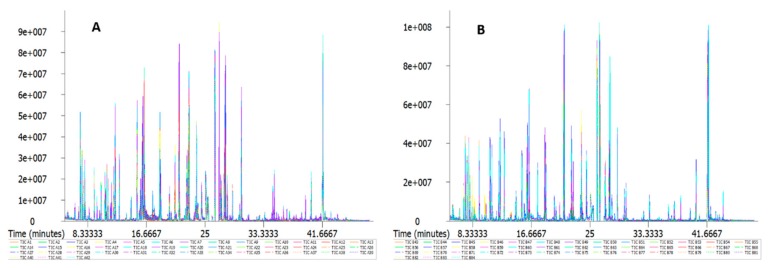
(**A**) (strain 18) and (**B**) (strain 18N44) samples were aligned with the original peak of total ion current; seven repeated samples were taken for each treatment.

The nontargeted metabonomics method aims to analyze thousands of unknown metabolites. Its method of verification is different from that of the targeted analytical method, which is more challenging [22]. In this study, we first observed the morphological changes of *L. edodes* mycelia after high-temperature stress and determined the relevant physiological indicators to find the differences between them and summarize the mechanism of change. High-temperature heat shock at 37 °C was performed for two different strains (18 and 18 N44) and for different time periods (0, 4, 8, 12, 18, and 24 h). To ensure the accuracy and reliability of the experimental results, we tried to ensure the consistency of culture and treatment conditions. Seven repeated samples were taken for each treatment. All 84 samples were subjected to Metabolomics Ion-based Data Extraction Algorithm (MET-IDEA) for peak extraction. In Figure 3, ion currents shown in seven colors represent seven biological repeats. The ordinate indicates peak strength, and each peak represents one or more metabolites. If the peak strength is higher, the content of the substance is higher. The abscissa is the retention time, which represents the time required for a substance to reach the maximum concentration from the beginning of sample placement in the column. If the retention time is longer, it requires a longer time to separate the substance from the capillary column. We visualized the total ion current (TIC) of all samples (shown in the Supplementary Materials). Figure 3 shows that the original peak had a good alignment effect, and there was no significant peak drift. The instrumental analysis of all samples showed a strong signal, large peak capacity, good reproducibility of retention time, and high instrumental stability.

### 2.3. Identification of Compounds

Based on naming conditions, the matching ion peak, including the relative molecular mass of characteristic ions, peak times, and peak areas (i.e.; relative quantitative results), of nontargeted metabolites were obtained. The retention time indexes of the peaks detected by the instrument were compared with the values of the Fiehn database. If the difference from the Fiehn RI (the retention time index of this substance in the database) in the database is within ±5000, it is meaningful to characterize the measured peak as the substance. Then, compared with the values in the chemical abstracts service (CAS), KEGG and Pub Chem databases, the condition that the difference is within ±5000 was met. After the matching degrees in the databases were ranked from high to low, we will obtain the characterized target metabolites.

Based on the above screening principle, there were 1152 peaks detected from all samples and quality controls, and each chromatogram was normalized to the total area. Metabolites were identified by a library search and were confirmed by authentic standards. After filtering the missing values and discarding the peaks with large measurement errors (RSD, relative standard deviation > 30% in QCs), a data matrix with 297 metabolic features was retained for further analysis (shown in Supplementary Materials). According to the relative quantification of each substance, a thermal map of the relative content of metabolites was obtained. The obtained metabolites were grouped according to the Human Metabolome Database (HMDB) (http://www.hmdb.ca/). Three main families of compounds were found, namely, amino acids, organic acids, and sugars/glycols.

### 2.4. Data Dimension Reduction Processing Result

**Figure 4:**
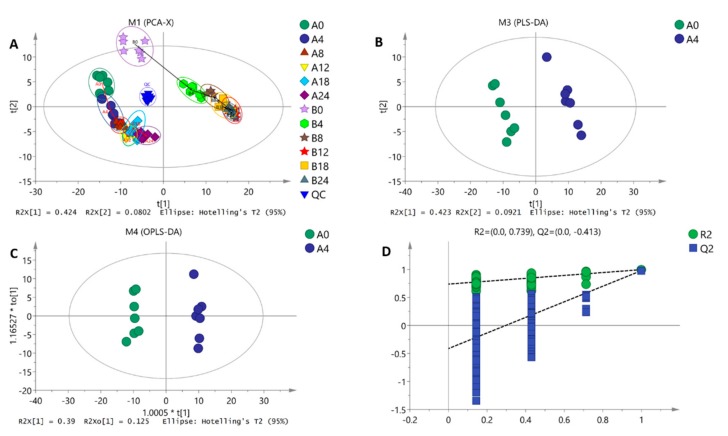
SIMCA software (V14) was used to perform multivariate variable pattern recognition for the normalized data. (**A**) Principal component analysis score plot of two strains treated at different times and quality control samples. (**B**,**C**) Score plot of partial least square discriminant analysis (PLS-DA) and orthogonal partial least square discriminant analysis (OPLS-DA) derived from the GC–MS profiles of serum samples obtained from the heat stress (4 h) group versus the normal control (0 h) group. (**D**) Response ordering test for the OPLS-DA model. (**B**–**D**) show strain 18 at 4 h versus 0 h as an example, and others are shown in Supplementary Materials.

Data dimension reduction was performed by principal component analysis (PCA), partial least square discriminant analysis (PLS-DA), orthogonal partial least square discriminant analysis (OPLS-DA), and a permutation test. We tried to apply PCA to give an overview of the distinctions between the A and B groups. The SMART method [23] was applied to remove the biological variation present among the pretreated groups. After UV scaling (mean-centered and then divided by the standard deviation), an unsupervised PCA model was built for each variety. The processing result is shown in Figure 4A. The blue triangle in the center represents quality control samples gathering together. It shows that the instrument has good stability and reliable detection data and can be used for subsequent tests. There are significant differences in PCA scores between strain 18 and strain 18N44. Both of them are far into the scoring map. The repeatability of each component is also good, within a 95% confidence interval. The PCA score map shows that the two groups of samples have a significant separation, which reflects the metabolic differences between the two groups. To more clearly visualize the temporal change of the metabolic statuses, trajectories based on the PCA scores were depicted where each point represents the average score of seven replicates. The metabolic trajectories of treated and control samples show very different shapes. The dynamic metabolomic alterations among untreated groups are associated with the growth of *L. edodes* during the 0−24 h period.

There are significant differences (spectral separation) in the PLS-DA score map between the two groups of samples (Figure 4B). The model interpretation rate R^2^Y and prediction rate Q^2^ are high. This shows that the PLS-DA model better explains and predicts the difference between the two groups of samples.

We performed a 200-fold response ranking test for the OPLS-DA model. That is, we fixed the X matrix, randomly arranging variables of the categorized Y matrix previously defined (e.g.; 0 or 1) *n* times (*n* = 200) to establish the corresponding OPLS-DA model for R^2^ and Q^2^ of the random model. After performing a linear regression of R^2^Y and Q^2^Y of the original model, the intercepts of the regression line and y axis are R^2^ and Q^2^, respectively, for measuring whether the model is overfitted. Taking strain 18 at 4 h versus 0 h as an example (others are shown in Supplementary Materials), it can be found from Figure 4C that, in the first dimension of predicted principal components, the scores of metabolites between the two groups are quite different, indicating that there is a great difference between the metabolites of the two components.

The quality of the model was examined by 7-fold cross verification. The model evaluation parameters obtained from R^2^X, R^2^Y, and Q^2^ were 0.515, 0.991, and 0.936, respectively. Among them, Q^2^ was close to 1, showing that the predictability of the model was strong. After 200 randomly repeated tests, the results shown in Figure 4D were obtained. All the points on the left are lower than the two original points of the same color on the right. At the same time, the permutation test intercept R^2^ = 0.789 and Q^2^ = −0.417, which can better reflect the robustness of the model. Therefore, the OPLS-DA model established in this paper can provide support for follow-up data analysis.

### 2.5. Screening of Differential Metabolites and Pathway Enrichment Analysis

Multidimensional analysis and one-dimensional analysis were combined to screen the different metabolites between groups. The screening was based on the VIP (variable importance in the projection) (threshold value > 1) of the first principle component of the OPLS-DA model and the *p* value (threshold value < 0.05) from the Student’s *t*-test. Differential metabolites were obtained among different treatments. We determined the change-multiplied FC (fold change) of differential metabolites among the two groups. The change multiple was the ratio of the average contents of the different metabolites among the two groups.

**Figure 5:**
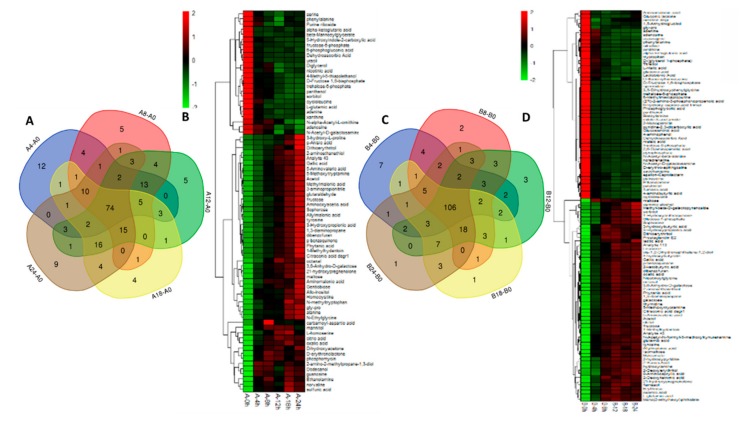
Venn diagram and heat map of different metabolites. (**A**,**C**) Venn diagram analysis of different metabolites from strains 18 and 18N44 under different heat stress durations. (**B**,**D**) Using Venn diagram analysis results, the heat map shows the significantly different metabolite changes of strains 18 and 18N44 under different heat stress times.

In strain 18, a total of 74 differential metabolites were obtained by intersecting differential metabolites under different treatments (Figure 5A). Four patterns were detected on the basis of the correlative analysis of changing trends in the metabolites (Figure 5B).

In strain 18N44, a total of 106 differential metabolites were obtained by intersecting differential metabolites under different treatments (Figure 5C). Metabolites were also divided into four groups according to the similarity of response patterns (Figure 5D).

Through the change in color gradation shown in Figure 5 and cluster analysis, the up- and downregulation of differential metabolites over time can be obtained. The comparison of the two strains under different treatments revealed 47 metabolites with great differences (Table 1). Among them, there are 11 amino acid metabolites, accounting for 23.4% of differential substances and five sugar metabolites, accounting for 11.9%.

**Figure 6:**
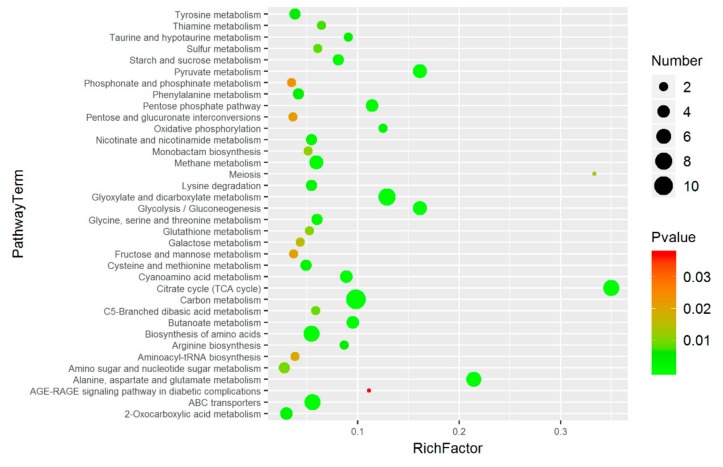
Pathway enrichment analysis of metabolites. A color-coded bar on the right represents the levels of significance. The green color indicates extremely significant levels at (*p* < 0.01), while red color indicates significant levels at (*p* < 0.05). The circles represent the number of metabolites involved or enriched in the pathway. Enrich factor refers to the ratio of the number of differential metabolites expressed in the corresponding pathway to the total number of metabolites annotated by the pathway. The greater the value the greater is the degree of the enrichment.

Pathway enrichment analysis of differential metabolites is helpful to understand the mechanism of metabolic pathway changes in different samples. The common pathway analysis is based on the KEGG-based metabolic pathway analysis (http://www.genome.jp/KEGG/pathway.html). Metabolic pathway maps containing two or more differential metabolites can be obtained by mapping differential metabolites to the KEGG database. To obtain metabolic pathways with more significant meaning, the enrichment results shown in Figure 6 were obtained by pathway enrichment analysis. It can be seen that the following were markedly changed: metabolism of alanine, aspartic acid, and glutamic acid; tricarboxylic acid cycle (TCA cycle); glutathione metabolism; glycine, serine, and threonine metabolism.

### 2.6. Pathway Analysis of Significantly Differential Metabolites

**Figure 7:**
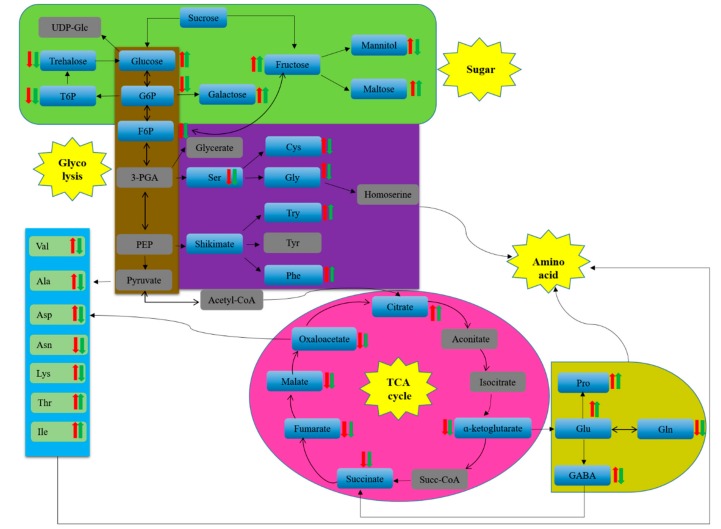
Schematic overview of some important metabolites and major metabolic pathways related to amino acid and energy metabolism in heat-stressed *L. edodes* mycelia. 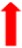 Strain 18 upregulation, 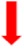 strain 18 downregulation. 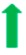 Strain 18N44 upregulation, 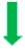 strain 18N44 downregulation. Gray indicates no significantly different metabolic.

With the pathway enrichment analysis results and the comparison of the relative contents of metabolites, the metabolic flow chart shown in Figure 7 was obtained. Comprehensive analysis shows that the contents of most amino acids in strain 18 were upregulated, while amino acids in strain 18N44 were downregulated. Intermediates in the glycolysis pathway and TCA cycle were downregulated. Glucose, maltose, galactose, and fructose were upregulated in glycometabolism. Mannitol increased significantly in strain 18, while mannitol decreased in strain 18N44, but trehalose decreased in two strains.

### 2.7. Targeted Metabolomic Analysis of Key Metabolites

**Figure 8:**
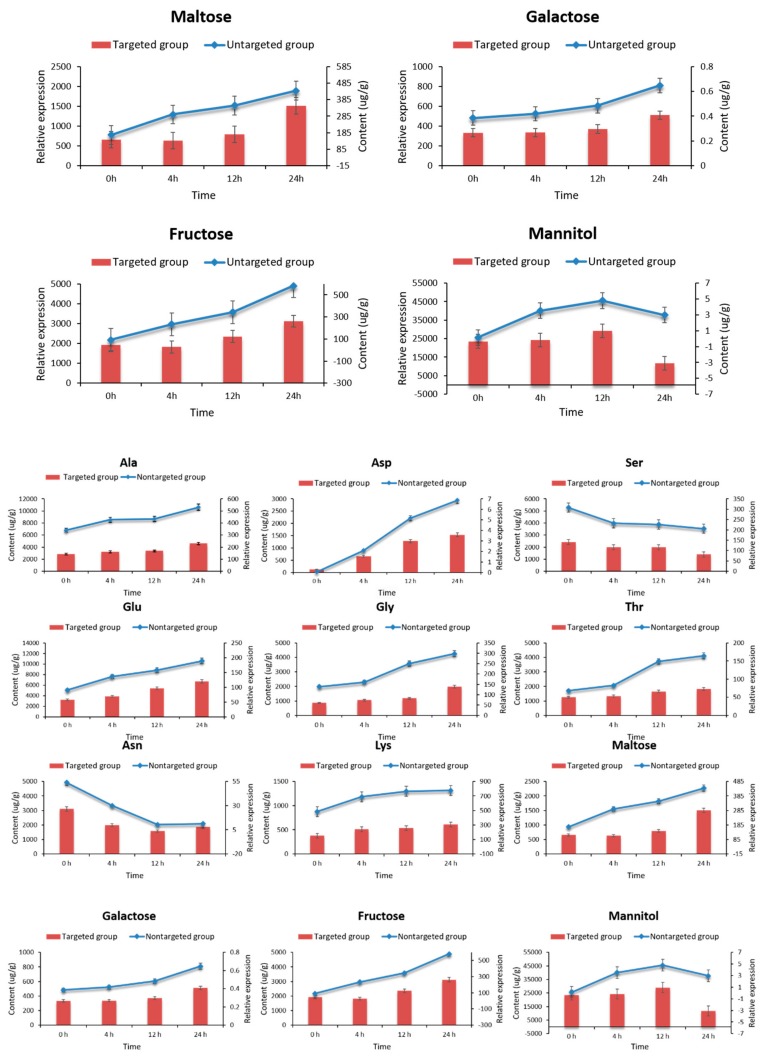
Verification of targeted metabolomics for the content changes of 12 metabolites. The targeted content determination result of 12 metabolites. The values are the means ± SD of three independent experiments. Note: Red bars with standard errors represent the content level determined by targeted GC–MS-based metabolomics (left Y-axis). Broken lines indicate the relative expression level determined by nontargeted GC–MS-based metabolomics (right Y-axis). All 12 metabolites were derived from strain 18.

Twelve amino acids and sugar alcohols with significant changes under high-temperature stress were selected for targeted verification. The results show that the expression trend of these metabolites was basically consistent with the overall trend of the targeted content determination (Figure 8). From this, the metabolomic analysis results accurately reflect the expression characteristics of *L. edodes* mycelia in response to high-temperature stress at the metabolic level.

## 3. Discussion

*L. edodes* is one of the variable-temperature fructifying mushrooms. Temperature is the primary environmental factor for its growth and development. It plays a decisive role in the growth, production, and quality of *L. edodes* [24]. High temperature is an important abiotic stress factor that leads to a decrease in the yield and quality of *L. edodes* [25]. High temperature can destroy the integrity of the cell wall and damage the cell membrane, thus altering the function of the plasma membrane structure, such as loss of selective permeability, resulting in increased fluidity and decreased permeability [3].

### 3.1. Physiological Study on Different Strains of *L. edodes* in Response to High-Temperature Stress

In this paper, the mycelial growth of *L. edodes* was significantly inhibited by high-temperature stress. Additionally, with the increase of high temperature duration, the inhibition was more obvious. It can be found from the physiological morphology that mycelia become sparse, forming a distinct heat stress circle. In addition, the mycelial branches of *L. edodes* were significantly decreased, and the recovery rate slowed down. The comparison of two strains showed that strain 18N44 responded first to high-temperature stress and was more sensitive to the stress. A change in conductivity can directly reflect damage to the cell membrane [26]. As mycelia undergo high-temperature stress, the cell membrane is destroyed, and the membrane permeability is increased, while the relative conductivity is increased due to electrolyte exudation. Therefore, the change in relative conductivity can directly reflect damage to the cell membrane. These changes can reflect damage to the cell membranes of the mycelial cells [27]. The study results show that high temperature can directly damage the cell membranes of the mycelia, leading to changes in cell permeability and electrolyte exudation, that is, an increase of relative conductivity. However, the relative conductivity of strain 18N44 was consistently higher than that of strain 18 during the entire process. This is also one of the reasons why strain 18N44 was the first to exhibit sensitivity to high-temperature stress. Moreover, free radicals are produced by the metabolism during aging and injury. However, scavenging capacity is decreased, resulting in the imbalance of reactive oxygen, which leads to membrane lipid peroxidation. Finally, malondialdehyde (MDA) is produced. The accumulation of MDA is an important indicator of senescence and damage to the cell membrane system [28,29]. MDA is also the product of membrane lipid peroxidation during the process of biological aging. It can be used as an indicator of membrane lipid peroxidation [30]. MDA is the most important product in the peroxidation process of polyunsaturated fatty acids and is often used as an indicator of oxidative stress caused by temperature [27]. The study results showed that the content of MDA in strain 18 increased with prolonged heat shock. This indicates that if the heat shock is longer, the oxidation of the cell membrane is more serious. For strain 18N44, the content of MDA decreased with prolonged heat shock. This is inconsistent with previous study results. This may be related to the characteristics of strain 18N44 itself and may also be related to the high-temperature resistance of mycelia. Further study is needed. The study results on the mycelial physiological indices show that mutant strain 18N44 was more sensitive to high-temperature stress than the starting strain 18. However, after removing high-temperature stress and restoring the normal growth temperature (25 °C), strain 18N44′s ability to regain growth was stronger than that of the sensitive strain 18. In addition, when the heat shock time exceeded 12 h, the growth rate was less affected. This may be related to the mechanism of self-response to high-temperature stress. Therefore, the ability of *L. edodes* mycelia to recover growth under heat stress can be used as an evaluation indicator of heat-resistant strains [31]. The mycelia branch is an important indicator to measure mycelial growth [32].

### 3.2. Metabonomic Study on Different Strains of *L. edodes* in Response to High-Temperature Stress

The metabolism can reflect living organisms and states. With the continuous improvement of detection and identification techniques for metabolites, metabolic regulation in response to abiotic stress has attracted widespread attention [33,34]. Metabonomics in the defense process under adverse situations has become a hot spot for extensive study, mainly involving some model plants, crop varieties, and yeasts in fungi [32,35,36,37,38]. In this paper, 297 metabolites are obtained using advanced GC–MS metabonomics at home and abroad. Seventy-four metabolites with significant differences in starting strain 18 were obtained by intersecting differential metabolites that were greatly changed in each treatment using a Venn diagram, while there were 106 important differential metabolites in the mutant strain 18N44. Finally, 47 key differential metabolites that changed greatly were obtained through comprehensive evaluation of the two strains. A series of metabolic pathway maps were obtained by KEGG mapping. Five distinct metabolic pathways were identified by pathway enrichment analysis. The main enriched substances included the following: amino acids, sugars or glycols, and intermediate products in glycolysis, and the TCA cycle.

Amino acids are a precursor of macromolecular proteins with biological functions and important nitrogen metabolites in organisms. Under environmental stress, free amino acids can make corresponding changes directly or indirectly to adapt to environmental changes [39]. This study shows that amino acid synthesis was almost upregulated after strain 18 underwent heat shock. It is suggested that amino acid synthesis may be a common response to heat shock. At the same time, most amino acids were downregulated in the mutant strain 18N44. This further shows that strain 18N44 is more sensitive than strain 18 to high-temperature stress.

Under high-temperature stress, glutamic acid (Glu) and aspartic acid (Asp) accumulate in strain 18, while Asp is constantly decomposed in strain 18N44. Liu Huaizhu (2015) et al believe that the accumulation of Glu and Asp can significantly improve the activities of superoxide dismutase (SOD) and ascorbate peroxidase (APX) in buckwheat leaves to eliminate the damaging effect of high-temperature stress on the activity of antioxidant enzymes and improve the heat resistance of buckwheat [40]. In addition, the increase of Glu and Asp can significantly reduce the plasma membrane permeability and MDA content of buckwheat leaves under high-temperature stress, which has a certain protective effect on the structure and function of the plasma membrane of buckwheat under high-temperature stress. Adverse situations (such as saline-alkali, drought, or cold-heat stress and mechanical damage) have a certain effect on the activity of glutamate decarboxylase (GAD) and the expression of glutamate decarboxylase and promote the synthesis of γ-aminobutyric acid (GABA) [29]. Some studies have shown that a large amount of GABA accumulates in plants under stress [41]. In this paper, affected by high temperature, GABA begins to accumulate in strain 18, while GABA begins to decompose in sensitive strain 18N44. At the same time, the contents of Ala and glycine (Gly) are significantly increased in strain 18, while both contents in sensitive strain 18N44 were decreased. Zhao Peng et al. (2015) found that, under different stresses (high temperature, heavy metals, H_2_O_2_, cold, UV), the regulatory protein Irr E affects the metabolism of amino acids in the mutant strain of *Deinococcus radiodurans* and participates in the regulation of alanine (Ala) metabolism, resulting in a real change in Ala content and affecting extreme stress resistance [42]. Gly plays a protective role in plants under adverse stress, especially in alleviating drought and low-temperature stress, or even has a bucking effect [43]. Gly’s response mechanism to stress is less studied. Based on Carini R et al.; it is inferred that Gly has a certain effect on the plasma membrane. Because its relative molecular mass is small, Gly can stabilize the membrane structure after binding with the cell membrane and prevent the loss of intracellular substances and the entry of unfavorable extracellular factors into the cell, which can alleviate the injury to the cell [44]. At high temperatures, most of the amino acids of *L. edodes* strains are upregulated. It is possible that some proteins denature and agglutinate after heat shock. To maintain normal life activities, it is necessary to synthesize new proteins continuously, which activates the amino acid synthesis pathway. However, the specific role of these amino acids in heat shock and the associated mechanism still needs further experimental verification.

Glycolysis and the TCA cycle are important components of *L. edodes* metabolism. The carbon skeleton they produce is essential for amino acid biosynthesis, energy metabolism, osmotic regulation, and cell balance. High-temperature stress disturbs the balance of carbon and nitrogen metabolism in *L. edodes*. High temperature hinders the normal flow of this pathway (Figure 7) and reduces the intermediate products of the glycolysis pathway. Contents of fructose-6-phosphoric acid, glucose-6-phosphoric acid, glucose-1-phosphoric acid, and pyruvic acid decreased. This indicates that the hexose pool shrinks at high temperature. These source products ultimately limit effective glycolysis, resulting in carbon starvation and energy deficiency of *L. edodes*. Metabolic map analysis of *L. edodes* mycelia shows that the TCA cycle of strains is slow at high temperature, and the level of organic acids involved in the TCA cycle decreases. This shows that the TCA cycle has been blocked, and energy metabolism has been reduced. Even respiratory function has been impaired, which hinders the input of mitochondrial proteins [45]. Although citric acids increase at high temperature, other organic acids decrease. TCA metabolism is lower than the control level at high temperature.

Under high-temperature stress, plants adapt to osmotic stress caused by high temperature through accumulating osmosis-regulating materials with low molecular weight [19]. Our study results show that, under high-temperature stress, various osmotic regulatory materials accumulate in *L. edodes* mycelia, such as sugar and sugar alcohol. The penetrating agent can stabilize the membrane structure and protect protein stability [46]. Trehalose is widely recognized as a protective sugar that protects cells from stress damage [47]. The trehalose content of *L. edodes* under high-temperature stress was determined. It is found that the trehalose content decreases in both strains 18 and 18N44. We made an analysis that trehalose is gradually reduced throughout the treatment at high temperatures, which may result from the combination of stress and transformation in the cell’s own growth phase. The stress reaction brought by the stress condition of the cell is not only reflected in the rapid accumulation of the trehalose but also reflected in the ability of cells to be dependent on the rapid degradation of trehalose under stress conditions. This is consistent with the results obtained by Li Lili et al. in studying the tolerance mechanism of *Saccharomyces cerevisiae* [48]. The relevant report shows that the decomposition of trehalose is to adjust the viscosity of the solution so as not to inhibit the enzyme activity and stabilize the protein structure, which can achieve the effect of protecting cells [49]. Trehalose metabolism is related to a variety of carbon metabolism. It can be decomposed into carbon sources for usage under long-term sustained stress [50]. Increasing at high temperature, galactose also acts as a protective membrane [51]. High temperature also promotes the accumulation of maltose to enhance its heat resistance [52]. Fructose and glucose are also increased significantly at high temperature. High temperature is accompanied by oxidative stress [53]. Polyols can stabilize macromolecules, effectively scavenge hydroxyl radicals, and prevent oxidative damage to membranes and enzymes. In this paper, at high temperature, glucose, galactose, fructose, and maltose accumulated in large quantities, mannitol increased significantly in strain 18, while mannitol decreased in strain 18N44, but trehalose decreased in the two strains. It also shows that strain 18 has strong osmotic-stress regulatory ability. However, excessive accumulation of sugar and polyols causes a large amount of carbon flow to enter its metabolic pathway. This weakens the central carbon metabolism and causes carbon starvation and energy metabolism deficiency [51].

In other words, the intermediate products of glycolysis and the TCA cycle decrease at high temperature, resulting in carbon starvation and insufficient energy metabolism, thus inhibiting the growth of *L. edodes*. High temperature results in aggravated protein degradation, greater energy consumption, and the accumulation of large amounts of free amino acids. This study shows that different species have different metabolic profiles in response to high-temperature stress, and different varieties of *L. edodes* have different metabolic profile expressions in response to high-temperature stress. These varieties often enhance their heat tolerance by accumulating a variety of amino acids and carbohydrates. Generally, the metabolites produced by different strains of *L. edodes* under high-temperature stress are basically the same, but different strains have species specificity. Therefore, the changes of metabolites involved in response to high-temperature stress have some differences. This is consistent with the study results for different species. Amino acids, intermediate metabolites in the TCA cycle, and many carbohydrate contents are affected by temperature stress [54,55,56,57,58,59].

Interestingly, it is found from the integration of the results of physiological studies and differential metabolomics that the mutant strain 18N44 of *L. edodes* is sensitive to high temperature, but the recovery ability of strain 18N44 is much higher than that of strain 18 after termination of the high-temperature stress. The possible reasons are as follows. When the external temperature rises, the cells respond quickly and are damaged in a short time. The normal metabolism of cells is out of control, and the internal structure of cells is disintegrated. Intracellular proteasomes undertake the tasks of degrading intracellular proteins, reducing their protein content and providing nutrients for the growth of their offspring. When the temperature becomes suitable for growth, the strain quickly resumes growth and resists the high-temperature environment, so it has the characteristics of high-temperature resistance. Strain 18 reacts slowly to high temperature. However, the continuous high temperature creates great damage to cells. When the temperature becomes suitable for growth, the growth is not easily resumed because of this damage. Therefore, temperature has a great influence at continuous high temperatures. To explain this phenomenon, further experimental verification is needed to determine the regulatory mechanism of the recovering strains.

## 4. Materials and Methods

### 4.1. Materials and Design

#### 4.1.1. Test Materials

Strain 18 used in this experiment is the main cultivar of *L. edodes*. Strain 18N44 was achieved with ultraviolet mutagenesis of strain 18 (starting strain) (Wang et al. 2014). Inter-simple sequence repeat (ISSR) was used to identify the two *L. edodes* strains mentioned above [60,61,62], to confirm that strain 18N44 is a new strain, with early production of mushrooms and fast growth. Thereafter, a pre-experiment of treatment temperature and treatment time under high-temperature stress was carried out to determine the optimal stress temperature and treatment time gradient [63]. Both strains were provided by the Institute of Edible Fungi, Shanghai Academy of Agricultural Sciences.

#### 4.1.2. Mycelial Culture and High-Temperature Stress Treatment

Figure 1A shows a schematic representation of the experimental design. The preserved *L. edodes* strain was transferred to a potato dextrose agar (medium) (PDA) plate (20 mL), which was incubated in a 25 °C thermostat for 5–7 d and then transferred to a new PDA plate. The activation treatment was repeated three to five times. The activated *L. edodes* strain was cultured for 15 d until the plate was full. Then, the strain and culture medium were transferred to a homogenizer together. After being broken, 10 mL of the substance was extracted by a pipette gun to a triangular bottle containing potato dextrose broth (PDB) (100 mL). After culturing at 25 °C at a speed of 150 r/min for 14 d, high-temperature stress was conducted. The cultured *L. edodes* mycelium was treated at 37 °C for 0 h, 4 h, 8 h, 12 h, 18 h, and 24 h [63]. Mycelia were collected rapidly on a super-clean workbench. The culture medium attached to the mycelium was washed with distilled water several times during collection. Seven repeated samples were taken for each treatment. Each sample was approximately 0.5 g, which was divided into seven pipes (seven biological repeats were needed, and split charging reduced the effects of repeated freezing and thawing). After being frozen in liquid nitrogen, samples were placed in an ultralow temperature refrigerator at −80 °C for later use.

### 4.2. Physiological Determination of Mycelia under High-Temperature Stress

#### 4.2.1. Morphology and Growth Rate of Mycelia

After 6 days of growth on PDA plates, the activated strains were treated by heat shock for different times (37 °C). Then, they were placed in a constant temperature box at 25 °C for 6 days of recovery culture. The growth of mycelia was recorded by regular photography and marking lines. Changes in mycelia were observed under fluorescent dye confocal microscopy.

#### 4.2.2. A Method for Determining the Conductivity of Mycelia Damaged by High Temperature

(1) The cultured mycelia were filtered with nonwoven fabric and then rinsed with 200 mL double-distilled water, and the mycelial balls were placed into a triangular bottle again.
(2) The cultured mycelial balls were treated by heat shock for 0 h, 4 h, 8 h, 12 h, 18 h, or 24 h. Then, the treated mycelial balls were put into 30 mL double-distilled water. At room temperature, the conductivity of the solution was determined to be E1.
(3) The centrifuge tube used in the conductivity measurements was sterilized at 121 °C for 20 min and shaken on a shaker at room temperature at 150 rpm for 24 h. The conductivity was measured at E2 at room temperature.
(4) Based on the following formula, the relative conductivity was determined as E% = (E1−E0)/(E2−E0), where E0 refers to the conductivity of double-distilled water [27].

#### 4.2.3. Determination of the Content of Malondialdehyde (MDA) in *L. edodes* Mycelia under High-Temperature Stress

To optimize Heath R L’s method [64], MDA content was measured by thiobarbituric acid chromatometry.

### 4.3. Chemicals

All chemicals and solvents were analytical or HPLC grade. Water, methanol, pyridine, n-hexane, methoxylamine hydrochloride (97%), and bis (trimethylsilyl) trifluoroacetamide (BSTFA) with 1% trimethylchlorosilane (TMCS) were purchased from CNW Technologies GmbH (Düsseldorf, Germany). Trichloromethane was obtained from Sinopharm Chemical Reagent Co.; Ltd. (Shanghai, China). L-2-chlorophenylalanine was obtained from Shanghai Hengchuang Biotechnology Co.; Ltd. (Shanghai, China).

### 4.4. Sample Preparation

A total of 120 mg of accurately weighed sample was transferred to a 1.5-mL Eppendorf tube. Two small steel balls were added to the tube. Then, 360 μL of cold methanol and 40 μL of 2-chloro-l-phenylalanine (0.3 mg/mL) dissolved in methanol as an internal standard were added to each sample, and the samples were placed at −80 °C for 2 min. Then, the samples were ground at 60 HZ for 2 min. The mixtures were ultrasonicated at ambient temperature for 30 min. Then, 200 μL of chloroform was added to the samples, the mixtures were vortexed, and 400 μL of water was added. Samples were vortexed again and then ultrasonicated at ambient temperature for 30 min. The samples were centrifuged at 12,000 rpm for 10 min at 4 °C. The quality control (QC) sample was prepared by mixing aliquots of all samples to form a pooled sample. An aliquot of 300 μL of supernatant was transferred to a glass sampling vial for vacuum drying at room temperature. Then, 80 μL of 15 mg/mL methoxylamine hydrochloride in pyridine was added. The resultant mixture was vortexed vigorously for 2 min and incubated at 37 °C for 90 min. Next, 80 μL of BSTFA (with 1% TMCS) and 20 μL n-hexane was added into the mixture, which was vortexed vigorously for 2 min and then derivatized at 70 °C for 60 min. The samples were placed at ambient temperature for 30 min before GC–MS analysis.

### 4.5. GC/MS Analysis

The derivatized samples were analyzed on an Agilent 7890B gas chromatography system coupled to an Agilent 5977A MSD system (Agilent Technologies Inc.; CA, USA). A DB-5 MS fused-silica capillary column (30 m × 0.25 mm × 0.25 μm, Agilent JW Scientific, Folsom, CA, USA) was utilized to separate the derivatives. Helium (>99.999%) was used as the carrier gas at a constant flow rate of 1 mL/min through the column. The injector temperature was maintained at 260 °C. The injection volume was 1 μL in split mode (split ratio is 4:1). The initial oven temperature was 60 °C, which was ramped to 125 °C at a rate of 8 °C/min, to 210 °C at a rate of 4 °C/min, to 270 °C at a rate of 5 °C/min, to 305 °C at a rate of 10 °C/min, and finally held at 305 °C for 3 min. The temperatures of quadrupole and ion source (electron impact) MS were set to 150 and 230 °C, respectively. The collision energy was 70 eV. Mass data were acquired in full-scan mode (m/z 50–500), and the solvent delay time was set to 5 min.

A QC sample was prepared by mixing the extracts of all samples in equal volume, and each QC had the same volume as the sample. The QCs were injected at regular intervals (every 10 samples) throughout the analytical run to provide a set of data from which repeatability could be assessed [65].

### 4.6. Data Preprocessing and Statistical Analysis

GC/MS original data (in D format) were converted to a universal format (in CDF format) by ChemStation (version E.02.02.1431, Agilent, CA, USA) analysis software. Then, Chroma TOF (version 4.34, LECO, St Joseph, MI, USA) was used to make pretreatment data, including peak extraction, denoising, deconvolution, and so on [66]. The National Institute of Standards and Technology (NIST) and Fiehn databases were used to characterize the metabolites. Finally, peak alignment was performed to derive the three-dimensional data matrix in CSV format (original data matrix). This three-dimensional matrix contains the following information: sample information, the names of peaks, retention time, mass-to-nucleus ratio, and mass spectrometry response intensity (peak area). An internal standard was used for data quality control. The internal standard peaks in the original data matrix and any known false positive peaks (including noise, column loss, and derivative reagent peaks) were removed, and the missing values were replaced by 0. In each sample, the signal intensity (peak area) of all peaks was normalized; that is, the signal intensity of each peak was converted into the relative intensity in the spectrum. After the data were normalized, they were multiplied by 10,000, and the data matrix was obtained by redundancy eliminating and peak merging.

SIMCA software (V14) was used to perform multivariate variable pattern recognition for the normalized data. After principal component analysis (PCA), partial least square discriminant analysis (PLS-DA) and orthogonal partial least square discriminant analysis (OPLS-DA) were performed. Then, the first and second principal components were modeled and analyzed. The quality of the model was checked by 7-fold cross-validation, and the corresponding Q^2^ value was obtained. By randomly changing the ranking order of the categorized variable y several times (*n* = 200), the corresponding intercept of a permutation test was obtained, and the validity of the model was further tested. By OPLS-DA analysis, irrelevant orthogonal signals were filtered to make differential metabolite detection more reliable. Mapping was performed in the Kyoto Encyclopedia of Genes and Genomes (KEGG) database to find the corresponding metabolic pathways and calculate the *p* value. Pathway enrichment analysis was performed by MetaboAnalyst 3.0. R language was used to make a heat map. For other drawings, Office series software and the data drawing tool Origin 9.1 were used.

### 4.7. Selection of Differential Metabolites

Differential metabolites were selected on the basis of the combination of a statistically significant threshold of variable influence on projection (VIP) values obtained from the OPLS-DA model and *p* values from a two-tailed Student’s *t*-test on the normalized peak areas, where metabolites with VIP values larger than 1 and *p* values less than 0.05 were included, respectively.

### 4.8. Determination of Targeted Metabolites in Key Heat Stress Substances

To verify the reliability of nontargeted metabonomics, 12 marker metabolite markers were selected from the screened differential metabolites and key pathways for targeted verification. The samples for nontargeted and targeted metabonomic experiments were cultured and collected in the same batch. Each sample was biologically repeated three times. Four key time points were selected for high-temperature stress treatment.

#### 4.8.1. Preparation of Standard Solution

The appropriate amount of standard sample was weighed precisely by an analytical balance and dissolved in solution A (water–acetonitrile, *v*/*v* = 98/2, containing 0.1% formic acid) to obtain the standard reserve solution. A mixed standard solution was prepared from the above standard sample with gradient dilutions of 200 µg/mL, 100 µg/mL, 50 µg/mL, 20 µg/mL, 10 µg/mL, 5 µg/mL, 2 µg/mL, 1 µg/mL, 0.5 µg/mL, and 0.1 µg/mL. These were put into 1.5 mL EP tubes, and 200 µL gradient concentration mixtures and solvent blanks were absorbed into derivative vials, respectively, before placement into a rapid centrifugal dryer for derivative treatment.

#### 4.8.2. Sample Treatment Shown in Section 4.4

#### 4.8.3. GC–MS Analysis Method Shown in Section 4.5

## 5. Conclusions

This paper focuses on different physiological characteristics of mutant strains and starting strains of *L. edodes* in response to high-temperature stress, explores the response mechanism to phenotypic changes by nontargeted metabonomics, and provides further validation by targeted metabonomics. High temperatures mainly affect amino acid metabolism, the glycolysis pathway, the TCA cycle, and the sugar metabolism of *L. edodes*. Most amino acids and carbohydrates enriched in these metabolic pathways are upregulated in strain 18, downregulated in strain 18N44, or those synthesized in strain 18 occur at higher levels than those in strain 18N44. Thus, the physiological phenotypic characteristics of strain 18N44 are more sensitive than those of the starting strain 18 to high temperature. At the same time, the intermediate products of glycolysis and the TCA cycle decrease under high temperature, resulting in carbon starvation and insufficient energy metabolism, thus inhibiting the growth of *L. edodes*. In addition, the results also show that the metabolites produced by different strains of *L. edodes* under high-temperature stress are basically the same, but different strains have species specificity. Therefore, the changes in the content of metabolites involved in response to high-temperature stress have some differences. This will provide a theoretical basis for further understanding the response mechanism of *L. edodes* to high temperature and can be used to establish an evaluation system of high-temperature-resistant strains and lay a foundation for molecular breeding of new high-temperature-resistant strains of *L. edodes*.

## Figures and Tables

**Table 1:** Identification of different metabolites.

Different Metabolites	VIP	*p*	Different Metabolites	VIP	*p*
Alanine	1.17812	4.88607 × 10^−4^	Gallic acid	1.30530	6.52562 × 10^−5^
Aspartate/aspartic acid	1.03574	3.17468 × 10^−7^	Ethanolamine	1.38919	4.47419 × 10^−10^
Glutamate/L-glutamic acid	1.35766	3.41897 × 10^−7^	2-aminoethanethiol	1.35147	1.89102 × 10^−6^
Proline	1.03591	2.860688 × 10^−3^	Dithioerythritol	1.38845	2.77433 × 10^−7^
Phenylalanine	1.07183	7.741247 × 10^−3^	Phytanic acid	1.40061	1.16715 × 10^−7^
Tyrosine	1.33479	4.72696 × 10^−4^	Adenine	1.38915	8.3489 × 10^−7^
Serine	1.46222	1.20382 × 10^−5^	Adenosine	1.16933	7.23195 × 10^−4^
L-glutamic acid	1.02125	7.3379 × 10^−5^	D-Fructose 1,6-bisphosphate	1.17656	5.699884 × 10^−3^
Glycine	1.22217	2.05583 × 10^−8^	Fructose-6-phosphate	1.36802	1.73526 × 10^−^^4^
Threonine	1.17647	2.305898 × 10^−3^	Alpha-ketoglutaric acid	1.38852	2.1916 × 10^−6^
Glutathione	1.25819	1.36582 × 10^−12^	1,3-diaminopropane	1.40000	4.07715 × 10^−8^
Citrate cycle	1.38953	6.38001 × 10^−10^	1-Methylhydantoin	1.34342	2.69444 × 10^−6^
Fructose	1.22170	5.66071 × 10^−8^	3,6-Anhydro-D-galactose	1.44528	7.65583 × 10^−12^
Maltose	1.24022	3.8289 × 10^−13^	5-Methoxytryptamine	1.42654	3.86987 × 10^−9^
Sophorose	1.23167	4.39968 × 10^−7^	Acetol	1.37941	4.87405 × 10^−8^
Trehalose	1.07189	2.184293 × 10^−3^	Allylmalonic acid	1.30494	2.91429 × 10^−5^
Sorbitol	1.32470	1.68173 × 10^−5^	Cycloleucine	1.36591	2.25922 × 10^−4^
3-Hydroxypropionic acid	1.41517	5.02333 × 10^−9^	Dibenzofuran	1.31198	5.32826 × 10^−6^
5-Aminovaleric acid	1.42903	2.50953 × 10^−9^	Gly-pro	1.40607	1.92801 × 10^−8^
Aminomalonic acid	1.33618	3.96316 × 10^−6^	N-Acetyl-D-galactosamine	1.03300	4.551565 × 10^−3^
Dehydroascorbic Acid	1.42405	5.98067 × 10^−6^	Octanal	1.39123	4.41212 × 10^−7^
Oxalic acid	1.27637	1.62664 × 10^−9^	Panthenol	1.25303	7.58015 × 10^−4^
Trehalose-6-phosphate	1.24712	4.56643 × 10^−6^	*p*-benzoquinone	1.35201	2.09886 × 10^−6^
Mannitol	1.22484	1.58142 × 10^−11^			

Note: The screening was based on the VIP (variable importance in the projection) (threshold value > 1) of the first principle component of the OPLS-DA model and the *p* value (threshold value < 0.05) from the Student’s *t*-test.

## Supplementary Materials

Supplementary Materials can be found at https://www.mdpi.com/1422-0067/20/9/2330/s1.

## References

[1] An J., Wang C., Liu G., Yang X., Zhang X., Li J. (2012). Analysis of volatile aroma components of fresh and dry *Lentinus edodes* with gas chromatography-mass spectrometry (GC-MS). Sci. Technol. Food Ind..

[2] Angelcheva L., Mishra Y., Antti H., Kjellsen T.D., Funk C., Strimbeck R.G., Schrã Der W.P. (2014). Metabolomic analysis of extreme freezing tolerance in Siberian spruce (Picea obovata). New Phytol..

[3] Ashraf M., Harris P.J.C. (2004). Potential biochemical indicators of salinity tolerance in plants. Plant Sci..

[4] Baldanzi G. (2007). Role of p38 map kinase in glycine-induced hepatocyte resistance to hypoxic injury. J. Hepatol..

[5] Bao Y., Yang N., Cang j., Feng M., lv Y., Peng G., Tian Y., Zhang D., Wang J., Meng J. (2017). Metabolomic Profiling of Winter Dongnongmai 1Grown at Different Temperatures. J. Triticeae Crop..

[6] Bowne J.B., Erwin T.A., Juttner J., Schnurbusch T., Langridge P., Bacic A., Roessner U. (2012). Drought Responses of Leaf Tissues from Wheat Cultivars of Differing Drought Tolerance at the Metabolite Level. Mol. Plant.

[7] Chen C. (2017). Degradation and Comparative Metabolomics of *Volvariella volvacea* Mycelium during Its Subculture. Ph.D. Thesis.

[8] Chen W., Yang Y., Li W., Jiang J., Yu H., Feng J., Li X., Liu K. (2016). Analysis of Volatile Components in *Lentinula edodes* by SPME-GC-MS and Establishment of Fingerprint. J. Food Sci. Biotechnol..

[9] Chu C.C., Chi T.H. (1986). Identification of sulfurous compounds of Shiitake mushroom (*Lentinus edodes* Sing). J. Agric. Food Chem..

[10] Cook D., Fowler S., Fiehn O., Thomashow M.F. (2004). A prominent role for the CBF cold response pathway in configuring the low-temperature metabolome of Arabidopsis. Proc. Natl. Acad. Sci. USA.

[11] Deng K., Zhang R. (1996). Determination of molecular weight of polymer by infrared spectroscopy. J. Hebei Univ..

[12] Fatma K., Joachim K., Yul S.D., Wei Z., Mick P., Ron P., Guy C.L. (2010). Transcript and metabolite profiling during cold acclimation of Arabidopsis reveals an intricate relationship of cold-regulated gene expression with modifications in metabolite content. Plant J. Cell Mol. Biol..

[13] Gray G.R., Heath D. (2010). A global reorganization of the metabolome in Arabidopsis during cold acclimation is revealed by metabolic fingerprinting. Physiol. Plant..

[14] Guy C., Kaplan F., Kopka J., Selbig J., Hincha D.K. (2010). Metabolomics of temperature stress. Physiol. Plant..

[15] Hannah M.A., Dana W., Susanne F., Oliver F., Heyer A.G., Hincha D.K. (2006). Natural genetic variation of freezing tolerance in Arabidopsis. Plant Physiol..

[16] Heath R.L., Packer L. (1968). Photoperoxidation in isolated chloroplasts: I. Kinetics and stoichiometry of fatty acid peroxidation. Arch. Biochem. Biophys..

[17] Hiraide M., Kato A., Nakashima T. (2010). The smell and odorous components of dried shiitake mushroom, Lentinula edodes V: Changes in lenthionine and lentinic acid contents during the drying process. J. Wood Sci..

[18] Hiraide M., Nakashima T., Fujiwara T. (2010). The smell and odorous components of dried shiitake mushroom, *Lentinula edodes* VI: Increase in odorous compounds of dried shiitake mushroom cultivated on bed logs. J. Wood Sci..

[19] Hodge S., Ward J.L., Beale M.H., Bennett M., Mansfield J.W., Powell G. (2013). Aphid-induced accumulation of trehalose in Arabidopsis thaliana is systemic and dependent upon aphid density. Planta.

[20] Huang J., Wu N., Song J., Zhang L., Jiang T., Li J. (2013). Effects of γ-Glutamyl-transpeptidase and Cysteine Sulfoxide Lyase on Endogenous Formaldehyde Production in Shiitake Mushroom. J. Chin. Inst. Food Sci. Technol..

[21] Huang N. (2010). Chinese Edible and Medicinal Bacteriology.

[22] Li W., Chen W., Yang Y., Zhang J., Feng J., Yu H. (2018). Volatile Flavor Components and Flavor Quality Evaluation of *Lentinula edodes* Harvested at Different Growth Stages. J. Nucl. Agric. Sci..

[23] Liu C. (2001). Distribution and biological characteristics of *Lentinus edodes*. For. By Prod. Spec. China.

[24] Liu H., Yang H. (2015). Physiological effects of glutamic acid and aspartic acid on buckwheat seedlings under high temperature stress. Jiangsu Agric. Sci..

[25] Long J., Wang X., Gao H., Liu Z., Liu C., Miao M., Liu J. (2006). Malonaldehyde acts as a mitochondrial toxin: Inhibitory effects on respiratory function and enzyme activities in isolated rat liver mitochondria. Life Sci..

[26] Lu L., Chen M., Xing Z., Chen G., Shao Y., Zhao X. (2013). Effect of Perforated Packaging on the Quality, Physiological and Biochemical Indexes of *Volvaria volvacea* Fruit Bodies during Storage. Acta Edulis Fungi.

[27] Keun H.C., Ebbels T.M., Bollard M.E., Beckonert O., Antti H., Holmes E., Lindon J.C., Nicholson J.K. (2004). Geometric trajectory analysis of metabolic responses to toxicity can define treatment specific profiles. Chem. Res. Toxicol..

[28] Ludmila R., Hongjian L., Joel S., Vladimir S., Sholpan D., Ron M. (2004). When defense pathways collide. The response of Arabidopsis to a combination of drought and heat stress. Plant Physiol..

[29] Mirzaei M., Yousefi M., Meskinfam M. (2012). Density functional studies of oxygen-terminations versus hydrogen-terminations in carbon and silicon nanotubes. Solid State Sci..

[30] Ni J., Yang X., Zhang H., Ni Y., Wu H., Wei Q. (2014). Metabolomics and Its Application to Plant Stress Research. World For. Res..

[31] Nicholson J.K., Lindon J.C., Holmes E. (1999). ‘Metabonomics’: Understanding the metabolic responses of living systems to pathophysiological stimuli via multivariate statistical analysis of biological NMR spectroscopic data. Xenobiotica.

[32] Nicolas B., Aaron F., David B., MoLler S.G., Hillel F. (2003). Mitochondrial succinic-semialdehyde dehydrogenase of the gamma-aminobutyrate shunt is required to restrict levels of reactive oxygen intermediates in plants. Proc. Natl. Acad. Sci. USA.

[33] Peng Z., Zhengfu Z., Wei Z., Min L., Ming C., Gehong W. (2015). Global transcriptional analysis of Escherichia coli expressing IrrE, a regulator from Deinococcus radiodurans, in response to NaCl shock. Mol. Biosyst..

[34] Pineau B., Bourge M., Marion J., Mauve C., Gilard F., Maneta-Peyret L., Moreau P., Satiat-Jeunemaitre B., Brown S.C., De Paepe R. (2013). The importance of cardiolipin synthase for mitochondrial ultrastructure, respiratory function, plant development, and stress responses in Arabidopsis. Plant Cell.

[35] Potters G., Pasternak T.P., Guisez Y., Palme K.J., Jansen M.A. (2007). Stress-induced morphogenic responses: Growing out of trouble?. Trends Plant Sci..

[36] Qin L.H., Song C.Y., Tan Q., Chen M., Pan Y. (2006). Use of ITS and ISSR markers to identify cultivated strains for Lentinula edodes. Mycosystema.

[37] Sampedro J.G., Uribe S. (2004). Trehalose-enzyme interactions result in structure stabilization and activity inhibition. The role of viscosity. Mol. Cell. Biochem..

[38] Shan X. (2016). Effect of Exogenous Spermidine on Carbon and Nitrogen Metabolism Mechanism in Tomato Seedlings under High Temperature Stress. Ph.D. Thesis.

[39] Shulaev V., Cortes D., Miller G., Mittler R. (2010). Metabolomics for plant stress response. Physiol. Plant..

[40] Wahid A., Close T.J. (2007). Expression of dehydrins under heat stress and their relationship with water relations of sugarcane leaves. Biol. Plant..

[41] Wang L., Zhao Y., Chen M. (2015). Monospore Cross-Breeding of Xianggu Mushroom (*Lentinula edodes*) ISSR Analysis of Thermo-Tolerant Hybrid. J. Microbiol..

[42] Wang L., Zhao Y., Zhang B., Chen M. (2014). Breeding thermo-tolerant strains of *Lentinula edodes* by UV mutagenesis. Sci. Technol. Food Ind..

[43] Want E.J., Wilson I.D., Helen G., Georgios T., Plumb R.S., John S., Elaine H., Nicholson J.K. (2010). Global metabolic profiling procedures for urine using UPLC-MS. Nat. Protoc..

[44] Warth B., Parich A., Bueschl C., Schoefbeck D., Neumann N.K.N., Kluger B., Schuster K., Krska R., Adam G., Lemmens M. (2015). GC–MS based targeted metabolic profiling identifies changes in the wheat metabolome following deoxynivalenol treatment. Metabolomics.

[45] Wei J. (2014). Analysis of Gene Expression Level and Activity of Key Antioxidant Enzymes from *Volvariella volvacea* in Response to Cold Stress. Ph.D. Thesis.

[46] Witt S., Galicia L., Lisec J., Cairns J., Tiessen A., Araus J.L., Palacios-Rojas N., Fernie A.R. (2012). Metabolic and phenotypic responses of greenhouse-grown maize hybrids to experimentally controlled drought stress. Mol. Plant.

[47] Wu G., Xie B., Jiang Y., Xiao K., Wang D., Peng C., Su Y. (2014). Effect of Different Packaging Regimes on the Quality of *Volvariella volvacea* Fruit Bodes. Acta Edulis Fungi.

[48] Xie F., Xie B., Lin Y., Yi H., Fu R. (2005). Effect of ^60^Co-γ Ray Irradiation on the Physiological and Biochemical Indexes and the Fresh-keeping of *V. volvacea*. Acta Edulis Fungi.

[49] Xie H.L., Pei-Wu L.I., Wang X.P., Zhang Q., Zhang L.X., Wang T., Zhang W., Wang X.F. (2017). Study on Effects of Temperature on Metabolism of Aspergillus Flavus Based on Untargeted Metabolomics. J. Instrum. Anal..

[50] Xin M. (2016). The Gene Expression and Function Study of Hydrophobin and Heat Shock Proteins from *Lentinula edodes*. Ph.D. Thesis.

[51] Xin M., Zhao Y., Huang J., Song C., Chen M. (2016). Expression and Bioinformatic Analysis of Hydrophobin Protein Gene (hyd1) in *Lentinula edodes* under High Temperature Stress. Mol. Plant Breed..

[52] Xu Z., Song X., Li Y., Yu C., Yan Z., Ming G., Shen X., Chen M. (2018). Gene expression related to trehalose metabolism and its effect on *Volvariella volvacea* under low temperature stress. Sci. Rep..

[53] Yang N., Wang C.L., He W.P., Qu Y.Z., Li Y.S. (2016). Photosynthetic characteristics and effects of exogenous glycine of Chorispora bungeana under drought stress. Photosynthetica.

[54] Ye Y., Zhu Y., Pan L., Li L., Wang X., Lin Y. (2009). Gaining insight into the response logic of Saccharomyces cerevisiae to heat shock by combining expression profiles with metabolic pathways. Biochem. Biophys. Res. Commun..

[55] Yuqing W. (2009). Identification of Mushroom of Germplasm Resources and the Preservation Methods. Ph.D. Thesis.

[56] Zhang J., Xie X., Dong Z. (2007). An evaluation on the heat tolerance of coolseason turf grasses under field heat stress. Pratacultural Sci..

[57] Zhao W., Guy C.L. (2004). Exploring the Temperature-Stress Metabolome of Arabidopsis. Plant Physiol..

[58] Zheng S., Zhang G., Zhang X., Cui L., Wang H. (2008). Effect of Different Temperature on the Storage of Different Water Content of Edible Mushrooms. Edible Fungi China.

[59] Zhou L., Li Y., Wang W., Zhong S. (2017). Research Progress in the Metabolomics for Plants Response to Temperature Stress. J. Shanxi Agric. Sci..

[60] Jia Z., Lei Z., Yuwei C., Xin L., Zhen Z., Guowang X. (2012). Alteration of leaf metabolism in Bt-transgenic rice (*Oryza sativa* L.) and its wild type under insecticide stress. J. Proteome Res..

[61] Jianrong L., Ju H., Jie Y., Ning W., Jun S., Lei Z., Tianjia J. (2012). Rapid purification and characterization of γ-glutamyl-transpeptidase from shiitake mushroom (*Lentinus edodes*). J. Food Sci..

[62] Jing-Yu L., Zheng-He Y., Fang L., Xin-Rui L., Bao-Gui X. (2012). Evaluation of the use of SCAR markers for screening genetic diversity of Lentinula edodes strains. Curr. Microbiol..

[63] Kantvilas G., Kirk P.M., Cannon P.F., David J.C., Stalpers J.A. (2001). Ainsworth Bisby’s Dictionary of the Fungi.

[64] Kind T., Wohlgemuth G., Lee D.Y., Lu Y., Palazoglu M., Shahbaz S., Fiehn O. (2009). FiehnLib: Mass spectral and retention index libraries for metabolomics based on quadrupole and time-of-flight gas chromatography/mass spectrometry. Anal. Chem..

[65] Kong H., Dai W., Xu G. (2014). Advances of metabolite identification in liquid chromatography-mass spectrometry based metabolomics. Chin. J. Chromatogr..

[66] Li L., Ye Y., Pan L., Zhu Y., Zheng S., Lin Y. (2009). The induction of trehalose and glycerol in Saccharomyces cerevisiae in response to various stresses. Biochem. Biophys. Res. Commun..

