# Supplementary material for: GC–MS-Based Nontargeted and Targeted Metabolic Profiling Identifies Changes in the Lentinula edodes Mycelial Metabolome under High-Temperature Stress
Source: Int J Mol Sci. 2019 May 10;20(9):2330. doi: 10.3390/ijms20092330 (PMC6539000; doi:10.3390/ijms20092330)
Supplement: Supplementary file 1 [file ijms-20-02330-s001.zip › supplementary material/2、Multivariate statistical analysis/Model parameters.docx]

| **No.** | **Model** | **Type** | **A** | **N** | **R2X(cum)** | **R2Y(cum)** | **Q2(cum)** | **R2** | **Q2** |
| --- | --- | --- | --- | --- | --- | --- | --- | --- | --- |
| all | M1 | PCA-X | 6 | 91 | 0.653 |  | 0.566 |  |  |
| A4-A0 | M2 | PCA-X | 2 | 14 | 0.538 |  | 0.32 |  |  |
| 3 | M3 | PLS-DA | 2 | 14 | 0.515 | 0.991 | 0.936 |  |  |
| 4 | M4 | OPLS-DA | 1+1+0 | 14 | 0.515 | 0.991 | 0.926 | 0.789 | -0.417 |
| A8-A0 | M5 | PCA-X | 2 | 14 | 0.6 |  | 0.388 |  |  |
| 6 | M6 | PLS-DA | 2 | 14 | 0.588 | 0.996 | 0.978 |  |  |
| 7 | M7 | OPLS-DA | 1+1+0 | 14 | 0.588 | 0.996 | 0.974 | 0.755 | -0.401 |
| A12-A0 | M8 | PCA-X | 2 | 14 | 0.606 |  | 0.411 |  |  |
| 9 | M9 | PLS-DA | 2 | 14 | 0.588 | 0.997 | 0.981 |  |  |
| 10 | M10 | OPLS-DA | 1+1+0 | 14 | 0.588 | 0.997 | 0.979 | 0.739 | -0.413 |
| A18-A0 | M11 | PCA-X | 2 | 14 | 0.615 |  | 0.432 |  |  |
| 12 | M12 | PLS-DA | 2 | 14 | 0.601 | 0.998 | 0.988 |  |  |
| 13 | M13 | OPLS-DA | 1+1+0 | 14 | 0.601 | 0.998 | 0.986 | 0.648 | -0.506 |
| A24-A0 | M14 | PCA-X | 2 | 14 | 0.63 |  | 0.45 |  |  |
| 15 | M15 | PLS-DA | 2 | 14 | 0.62 | 0.998 | 0.991 |  |  |
| 16 | M16 | OPLS-DA | 1+1+0 | 14 | 0.62 | 0.998 | 0.988 | 0.642 | -0.502 |
| B4-B0 | M17 | PCA-X | 2 | 14 | 0.642 |  | 0.409 |  |  |
| 18 | M18 | PLS-DA | 2 | 14 | 0.632 | 0.995 | 0.977 |  |  |
| 19 | M19 | OPLS-DA | 1+1+0 | 14 | 0.632 | 0.995 | 0.969 | 0.687 | -0.471 |
| B8-B0 | M20 | PCA-X | 2 | 14 | 0.679 |  | 0.511 |  |  |
| 21 | M21 | PLS-DA | 2 | 14 | 0.652 | 0.997 | 0.978 |  |  |
| 22 | M22 | OPLS-DA | 1+1+0 | 14 | 0.652 | 0.997 | 0.971 | 0.763 | -0.363 |
| B12-B0 | M23 | PCA-X | 2 | 14 | 0.706 |  | 0.521 |  |  |
| 24 | M24 | PLS-DA | 2 | 14 | 0.697 | 0.998 | 0.991 |  |  |
| 25 | M25 | OPLS-DA | 1+1+0 | 14 | 0.697 | 0.998 | 0.989 | 0.759 | -0.35 |
| B18-B0 | M26 | PCA-X | 2 | 14 | 0.689 |  | 0.496 |  |  |
| 27 | M27 | PLS-DA | 2 | 14 | 0.676 | 0.998 | 0.99 |  |  |
| 28 | M28 | OPLS-DA | 1+1+0 | 14 | 0.676 | 0.998 | 0.987 | 0.746 | -0.369 |
| B24-B0 | M29 | PCA-X | 2 | 14 | 0.691 |  | 0.503 |  |  |
| 30 | M30 | PLS-DA | 2 | 14 | 0.68 | 0.999 | 0.992 |  |  |
| 31 | M31 | OPLS-DA | 1+1+0 | 14 | 0.68 | 0.999 | 0.988 | 0.793 | -0.371 |
| B0-A0 | M32 | PCA-X | 2 | 14 | 0.579 |  | 0.363 |  |  |
| 33 | M33 | PLS-DA | 2 | 14 | 0.578 | 0.999 | 0.99 |  |  |
| 34 | M34 | OPLS-DA | 1+1+0 | 14 | 0.578 | 0.999 | 0.985 | 0.763 | -0.405 |
| B4-A4 | M35 | PCA-X | 2 | 14 | 0.707 |  | 0.555 |  |  |
| 36 | M36 | PLS-DA | 2 | 14 | 0.705 | 0.999 | 0.995 |  |  |
| 37 | M37 | OPLS-DA | 1+1+0 | 14 | 0.705 | 0.999 | 0.993 | 0.757 | -0.416 |
| B8-A8 | M38 | PCA-X | 2 | 14 | 0.748 |  | 0.634 |  |  |
| 39 | M39 | PLS-DA | 2 | 14 | 0.747 | 0.999 | 0.995 |  |  |
| 40 | M40 | OPLS-DA | 1+1+0 | 14 | 0.747 | 0.999 | 0.992 | 0.722 | -0.451 |
| B12-A12 | M41 | PCA-X | 2 | 14 | 0.726 |  | 0.59 |  |  |
| 42 | M42 | PLS-DA | 2 | 14 | 0.72 | 1 | 0.996 |  |  |
| 43 | M43 | OPLS-DA | 1+1+0 | 14 | 0.72 | 1 | 0.994 | 0.783 | -0.367 |
| B18-A18 | M44 | PCA-X | 2 | 14 | 0.73 |  | 0.612 |  |  |
| 45 | M45 | PLS-DA | 2 | 14 | 0.714 | 1 | 0.995 |  |  |
| 46 | M46 | OPLS-DA | 1+1+0 | 14 | 0.714 | 1 | 0.994 | 0.723 | -0.423 |
| B24-A24 | M47 | PCA-X | 2 | 14 | 0.702 |  | 0.568 |  |  |
| 48 | M48 | PLS-DA | 2 | 14 | 0.692 | 0.999 | 0.994 |  |  |
| 49 | M49 | OPLS-DA | 1+1+0 | 14 | 0.692 | 0.999 | 0.992 | 0.69 | -0.537 |
