# Supplementary figures and images for: GC–MS-Based Nontargeted and Targeted Metabolic Profiling Identifies Changes in the Lentinula edodes Mycelial Metabolome under High-Temperature Stress
Source: Int J Mol Sci. 2019 May 10;20(9):2330. doi: 10.3390/ijms20092330 (PMC6539000; doi:10.3390/ijms20092330)

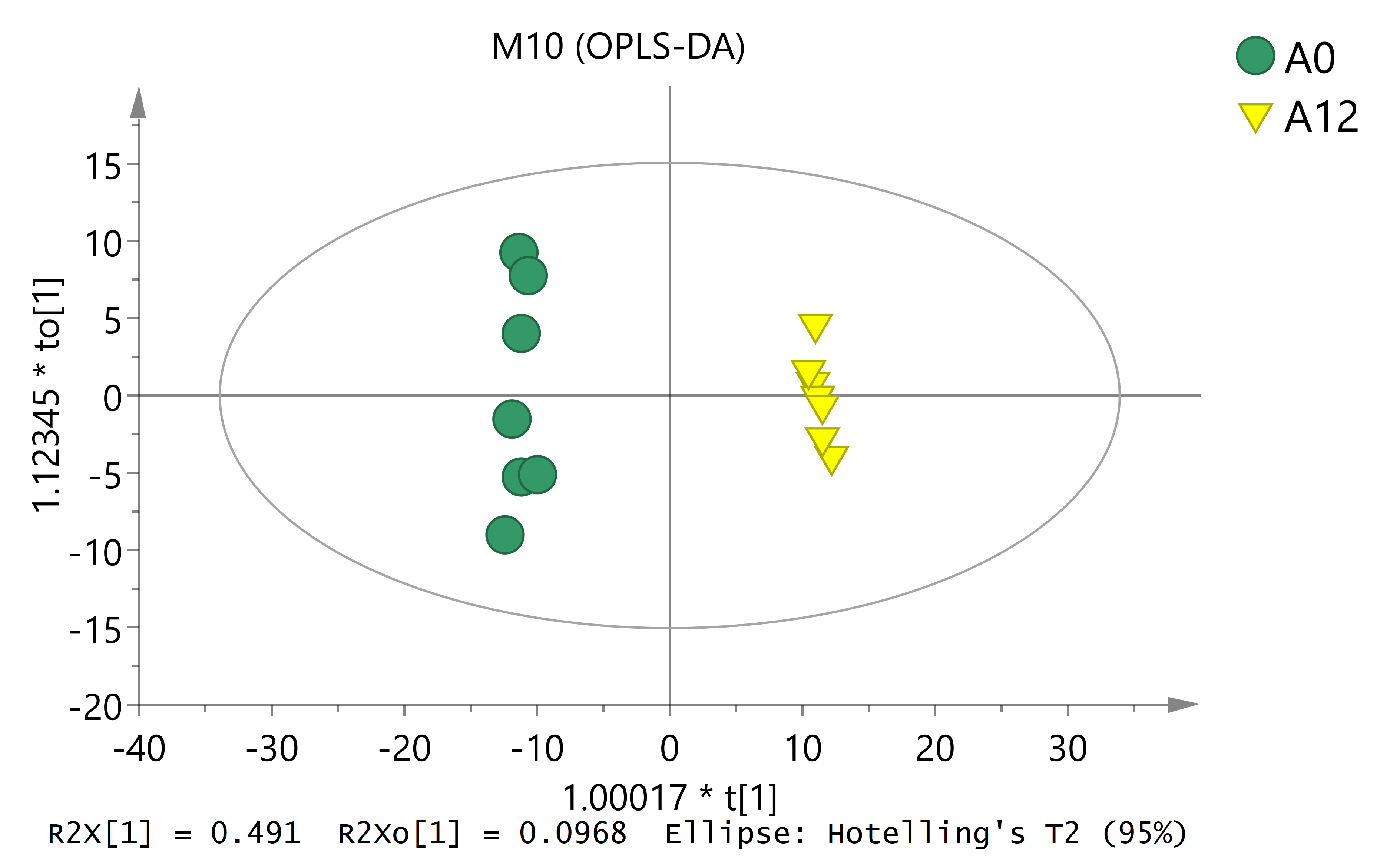

Supplement: Supplementary file 1 [file ijms-20-02330-s001.zip › supplementary material/2、Multivariate statistical analysis/opls(A0-12).tif]

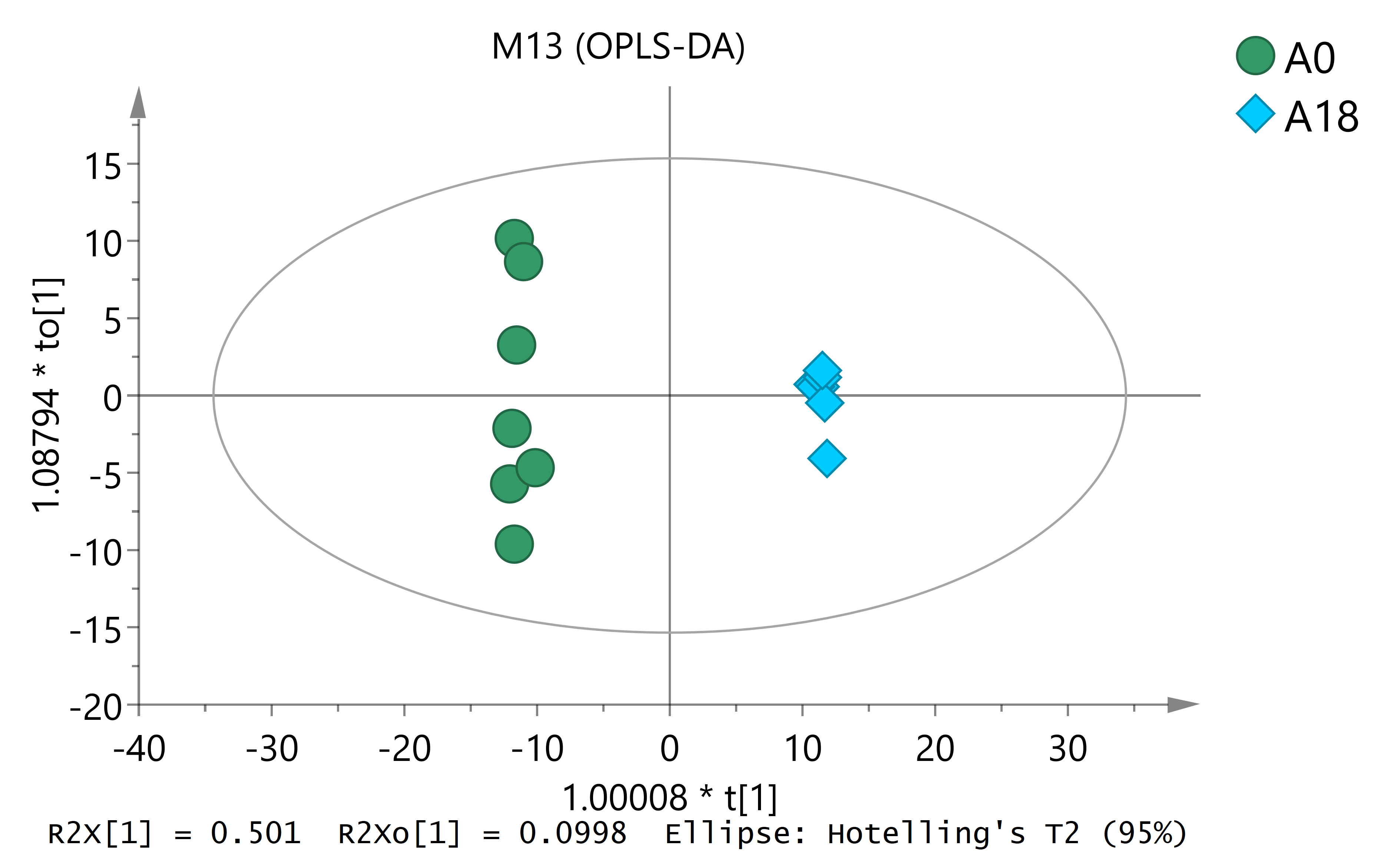

Supplement: Supplementary file 1 [file ijms-20-02330-s001.zip › supplementary material/2、Multivariate statistical analysis/opls(A0-18).tif]

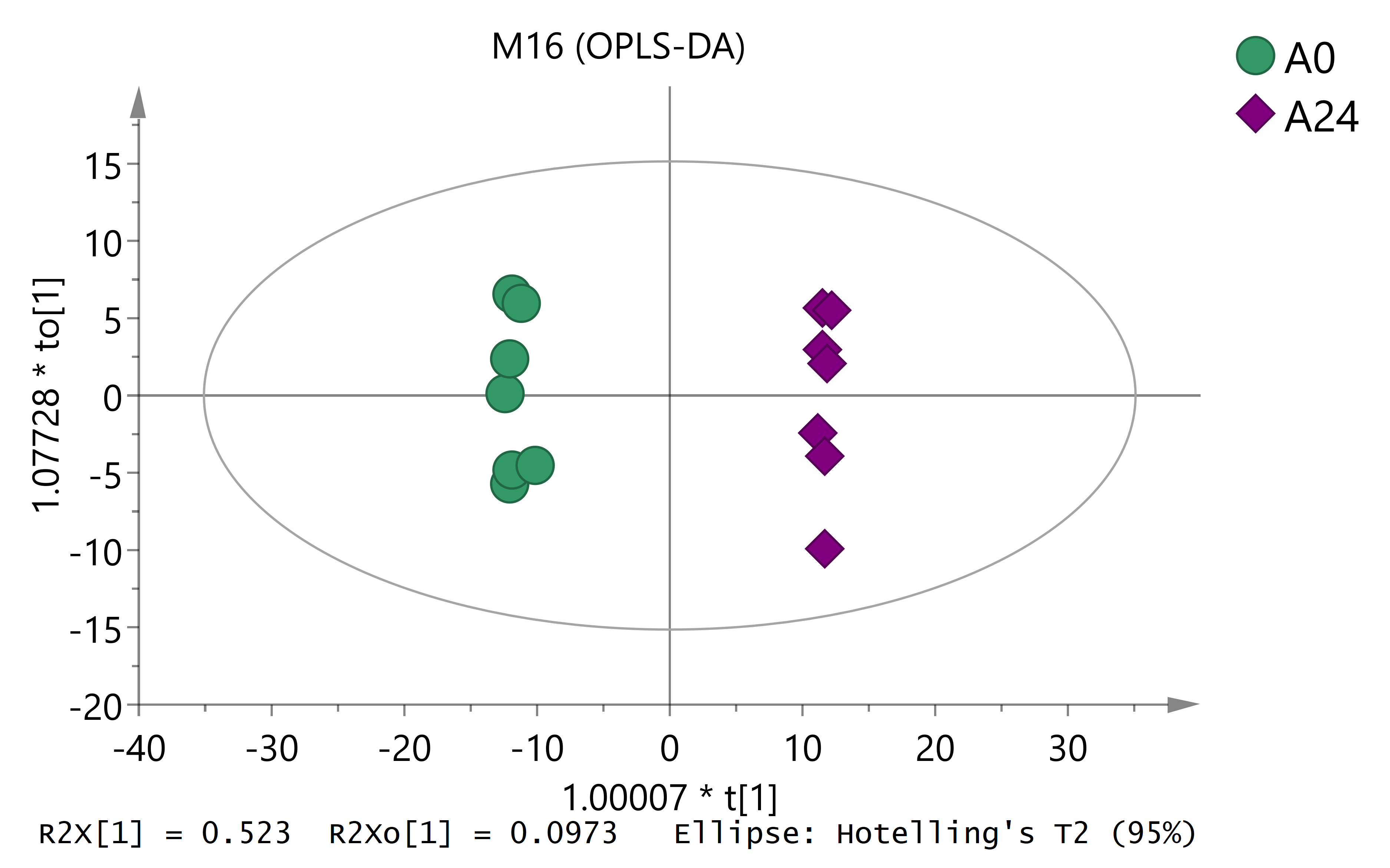

Supplement: Supplementary file 1 [file ijms-20-02330-s001.zip › supplementary material/2、Multivariate statistical analysis/opls(A0-24).tif]

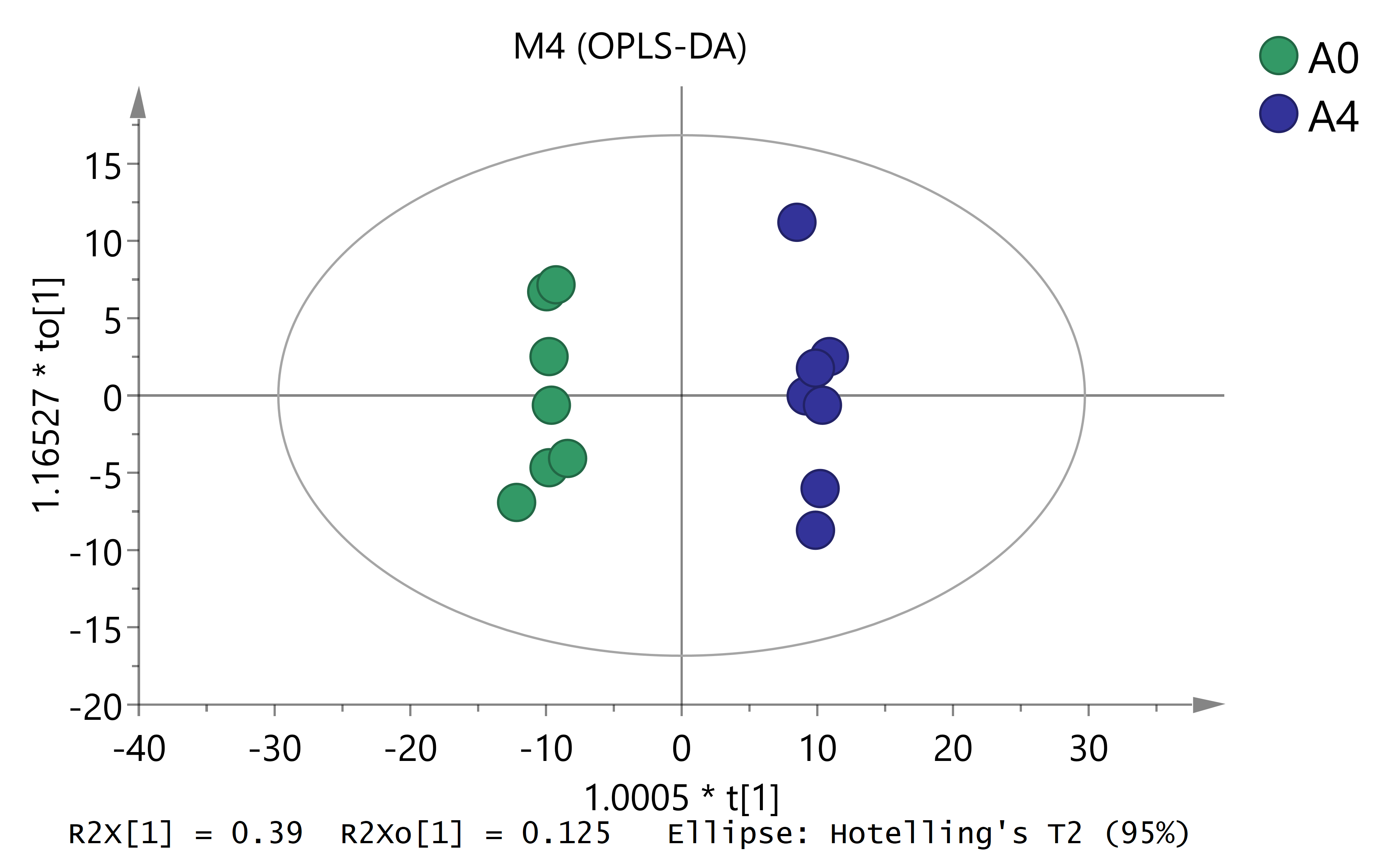

Supplement: Supplementary file 1 [file ijms-20-02330-s001.zip › supplementary material/2、Multivariate statistical analysis/opls(A0-4).tif]

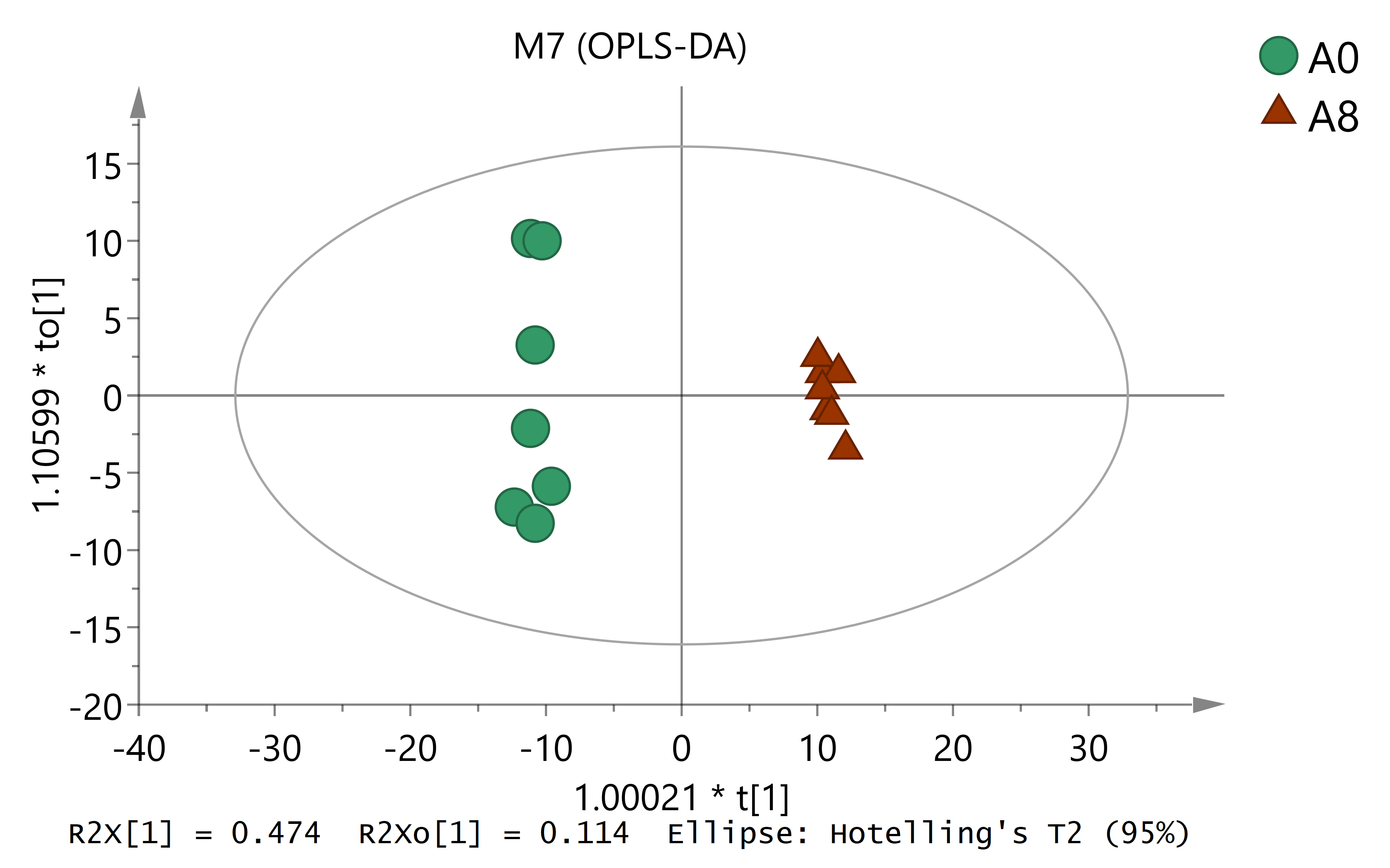

Supplement: Supplementary file 1 [file ijms-20-02330-s001.zip › supplementary material/2、Multivariate statistical analysis/opls(A0-8).tif]

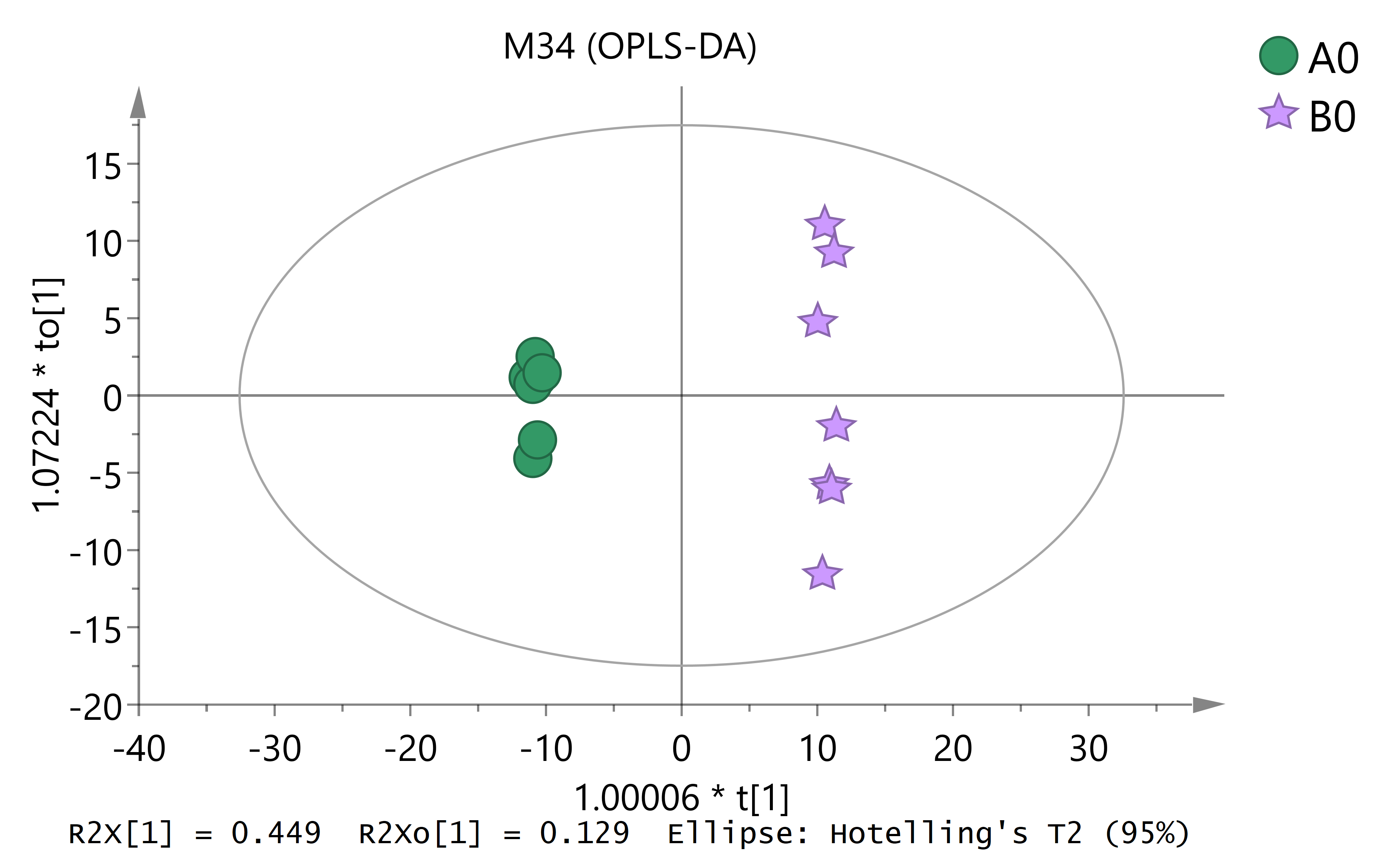

Supplement: Supplementary file 1 [file ijms-20-02330-s001.zip › supplementary material/2、Multivariate statistical analysis/opls(A0-B0).tif]

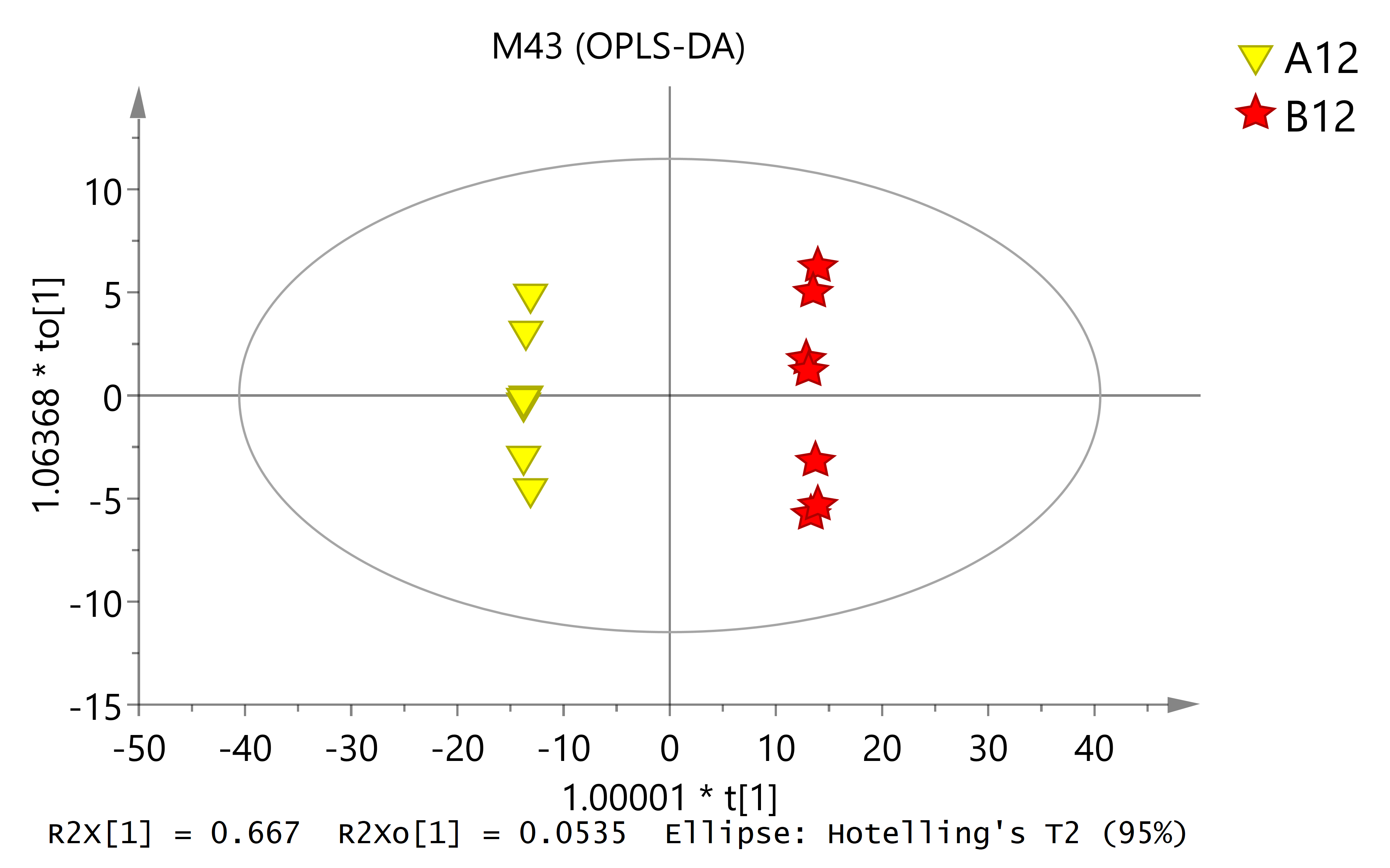

Supplement: Supplementary file 1 [file ijms-20-02330-s001.zip › supplementary material/2、Multivariate statistical analysis/opls(A12-B12).tif]

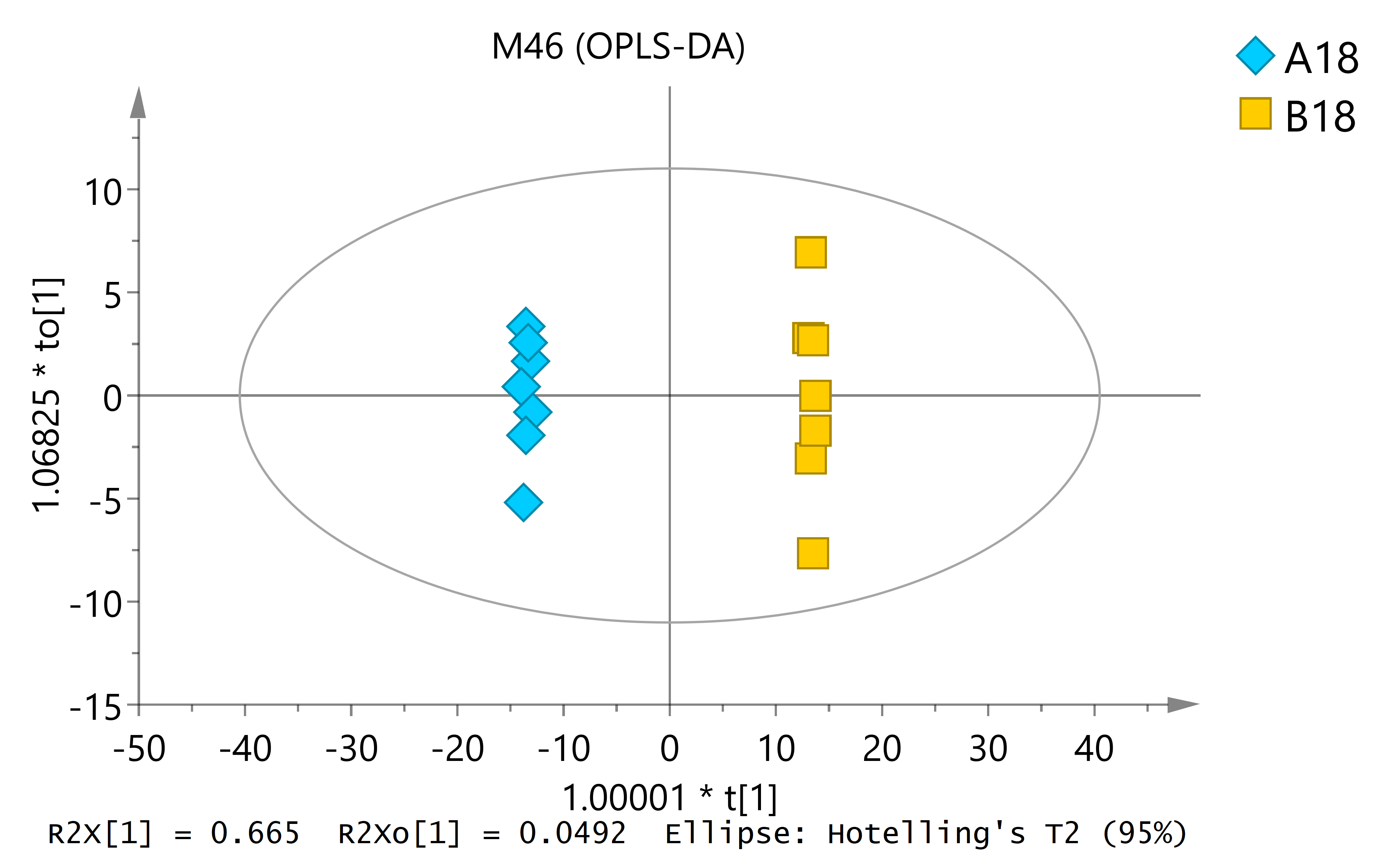

Supplement: Supplementary file 1 [file ijms-20-02330-s001.zip › supplementary material/2、Multivariate statistical analysis/opls(A18-B18).tif]

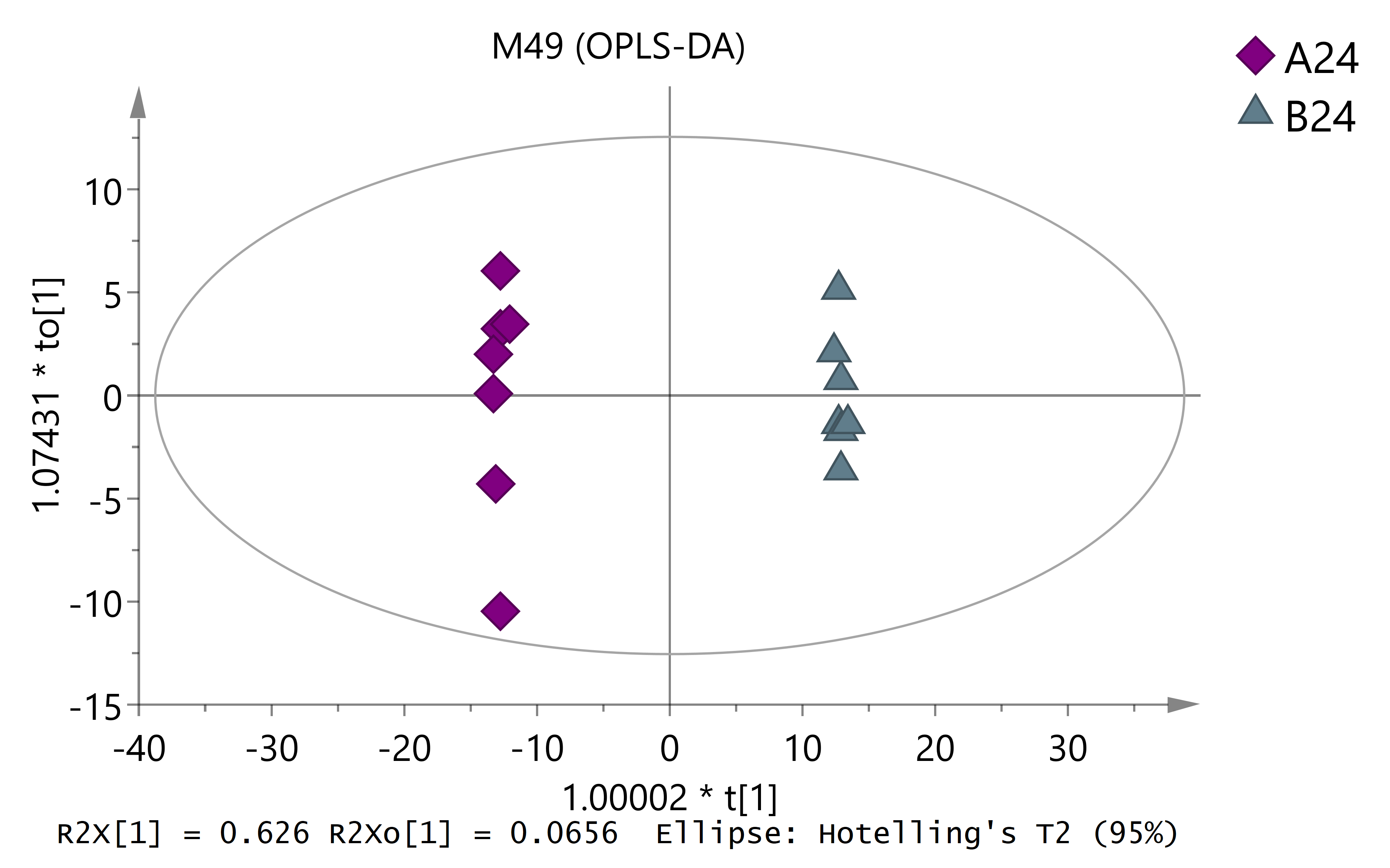

Supplement: Supplementary file 1 [file ijms-20-02330-s001.zip › supplementary material/2、Multivariate statistical analysis/opls(A24-B24).tif]

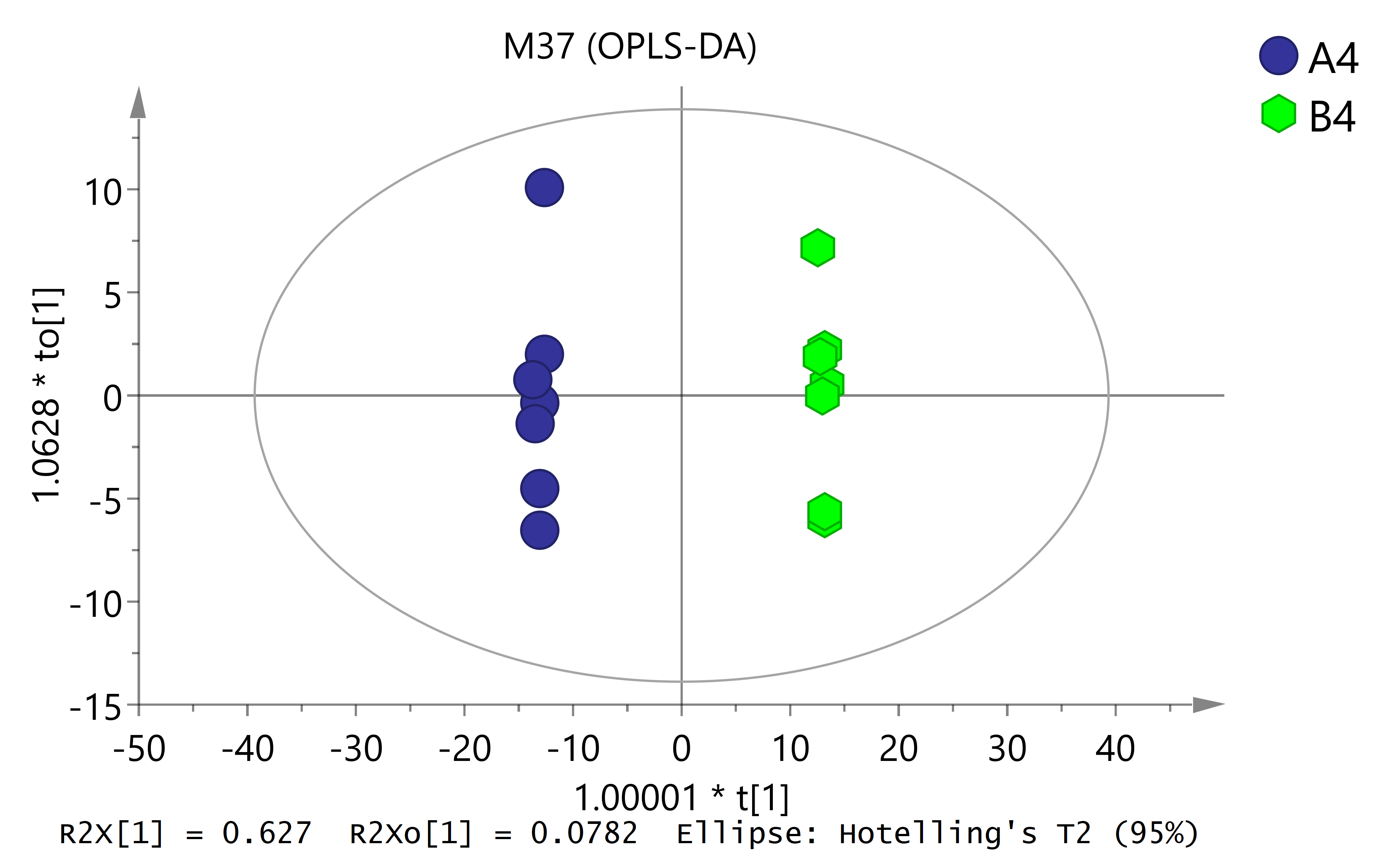

Supplement: Supplementary file 1 [file ijms-20-02330-s001.zip › supplementary material/2、Multivariate statistical analysis/opls(A4-B4).tif]

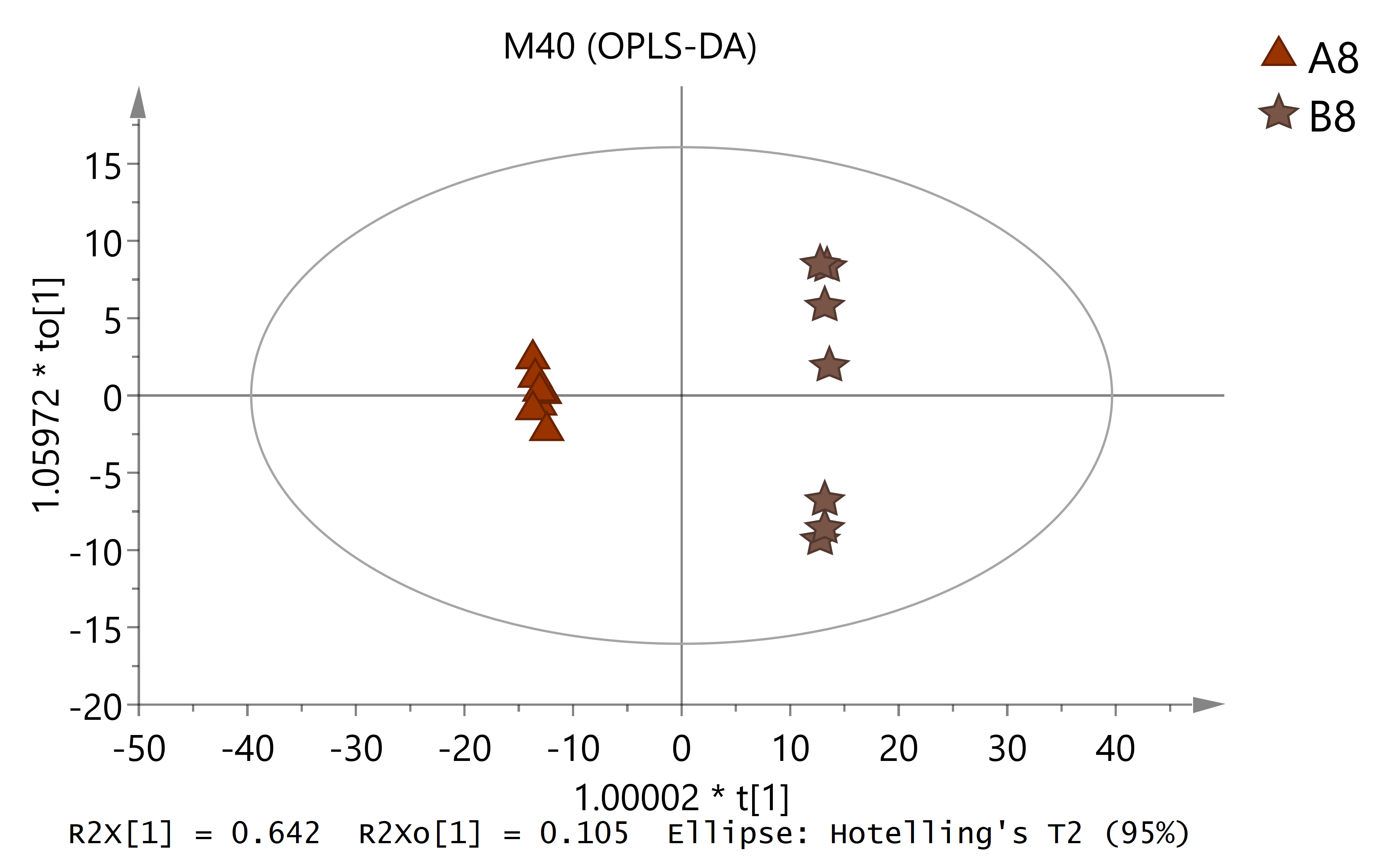

Supplement: Supplementary file 1 [file ijms-20-02330-s001.zip › supplementary material/2、Multivariate statistical analysis/opls(A8-B8).tif]

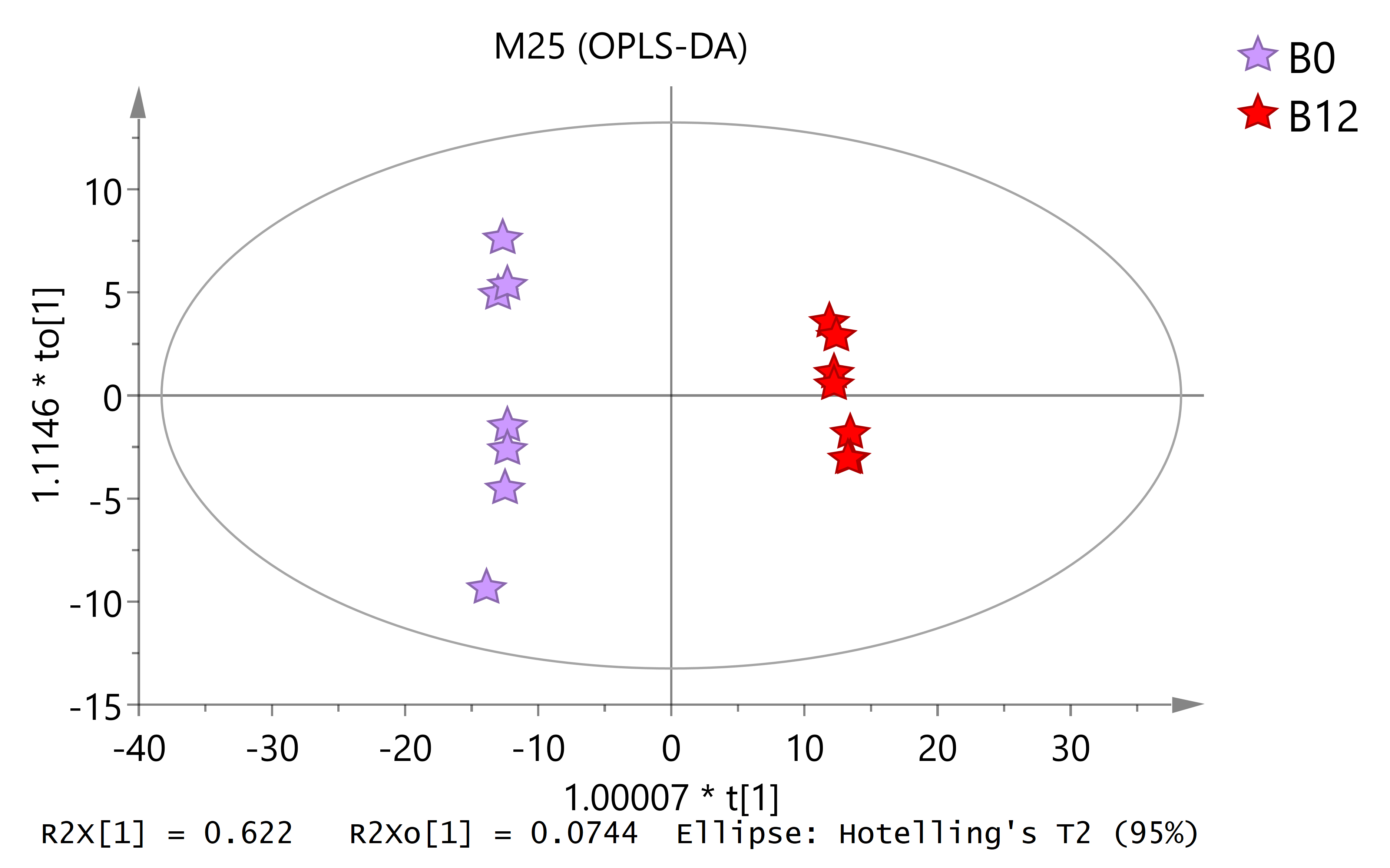

Supplement: Supplementary file 1 [file ijms-20-02330-s001.zip › supplementary material/2、Multivariate statistical analysis/opls(B0-12).tif]

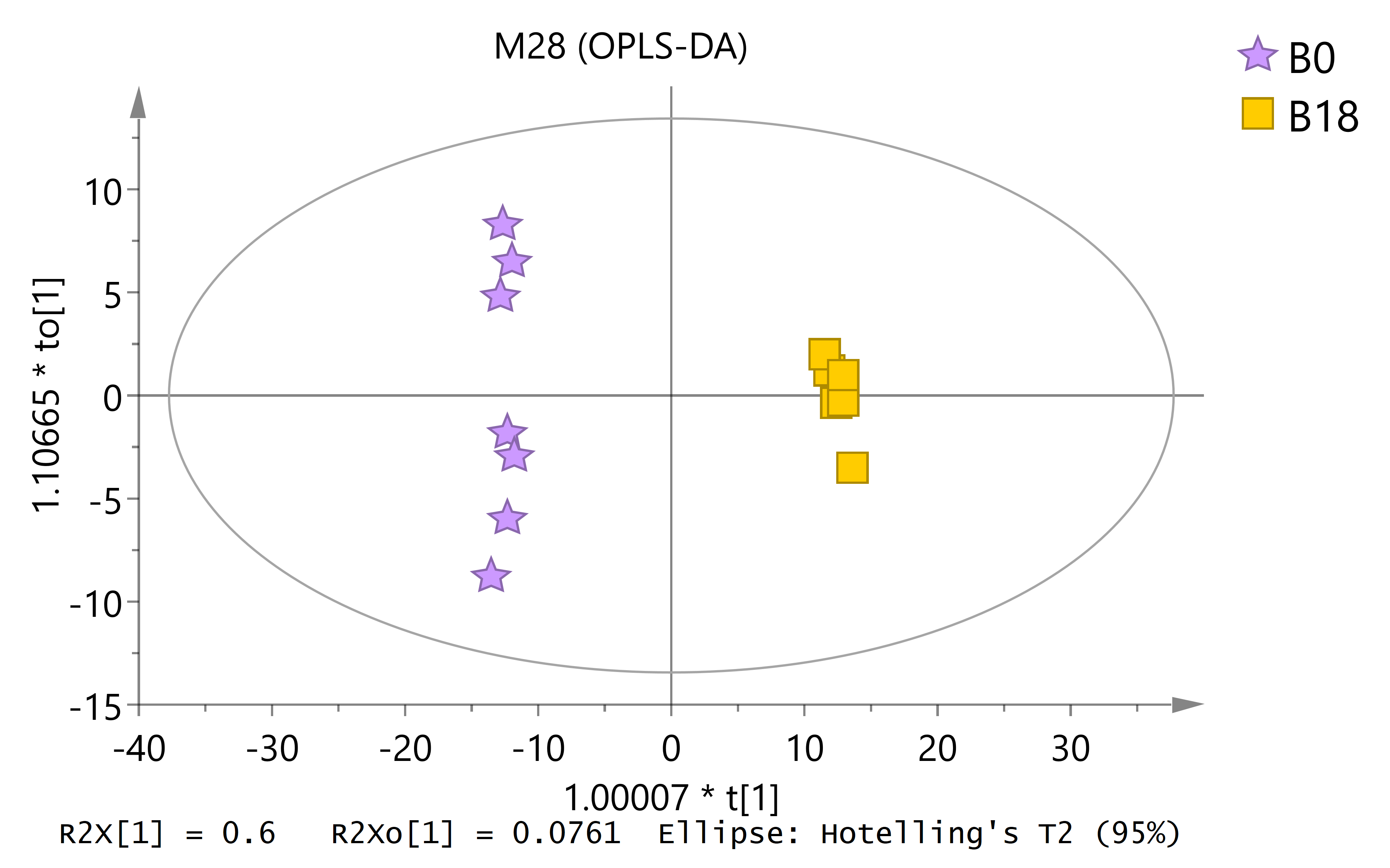

Supplement: Supplementary file 1 [file ijms-20-02330-s001.zip › supplementary material/2、Multivariate statistical analysis/opls(B0-18).tif]

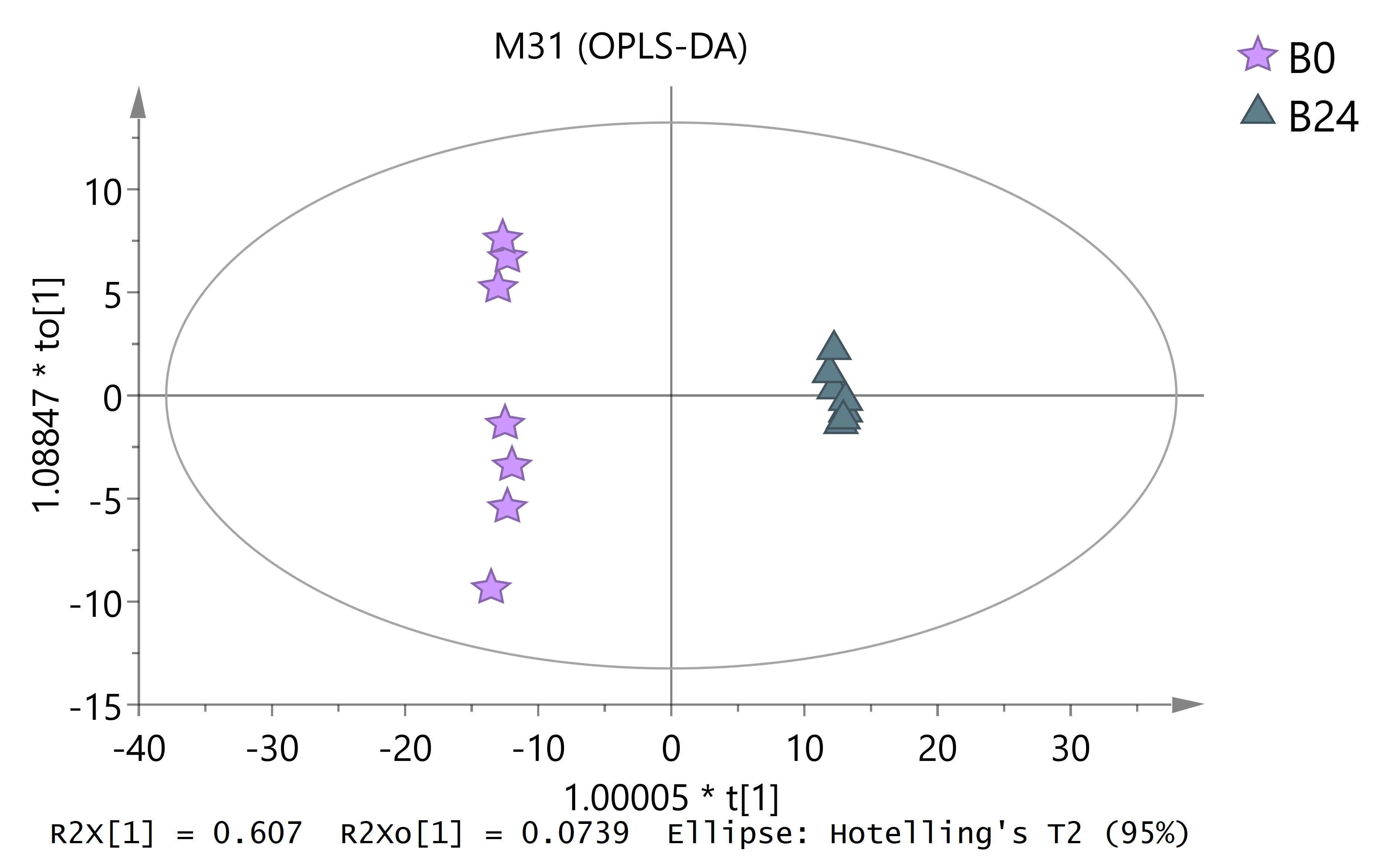

Supplement: Supplementary file 1 [file ijms-20-02330-s001.zip › supplementary material/2、Multivariate statistical analysis/opls(B0-24).tif]

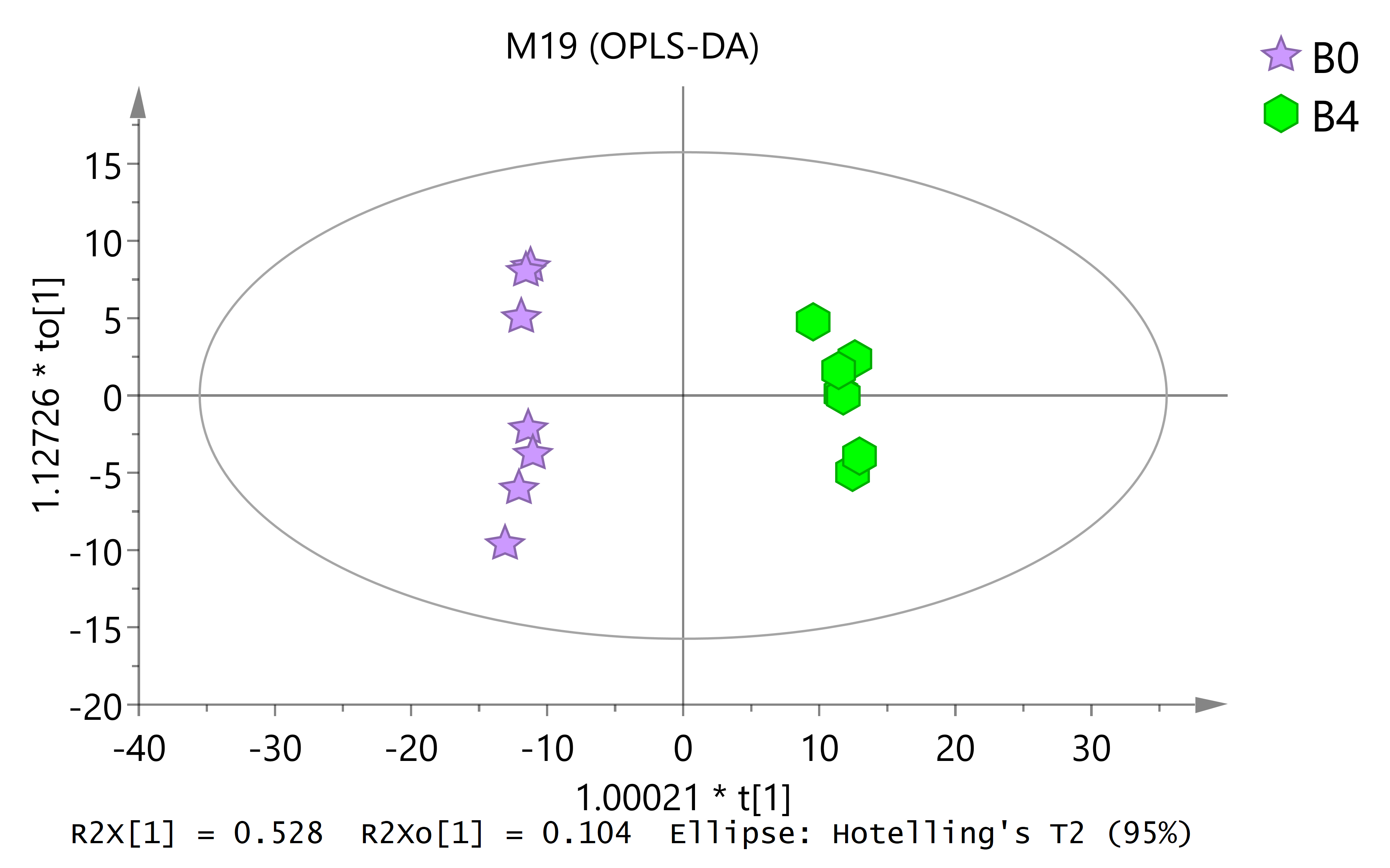

Supplement: Supplementary file 1 [file ijms-20-02330-s001.zip › supplementary material/2、Multivariate statistical analysis/opls(B0-4).tif]

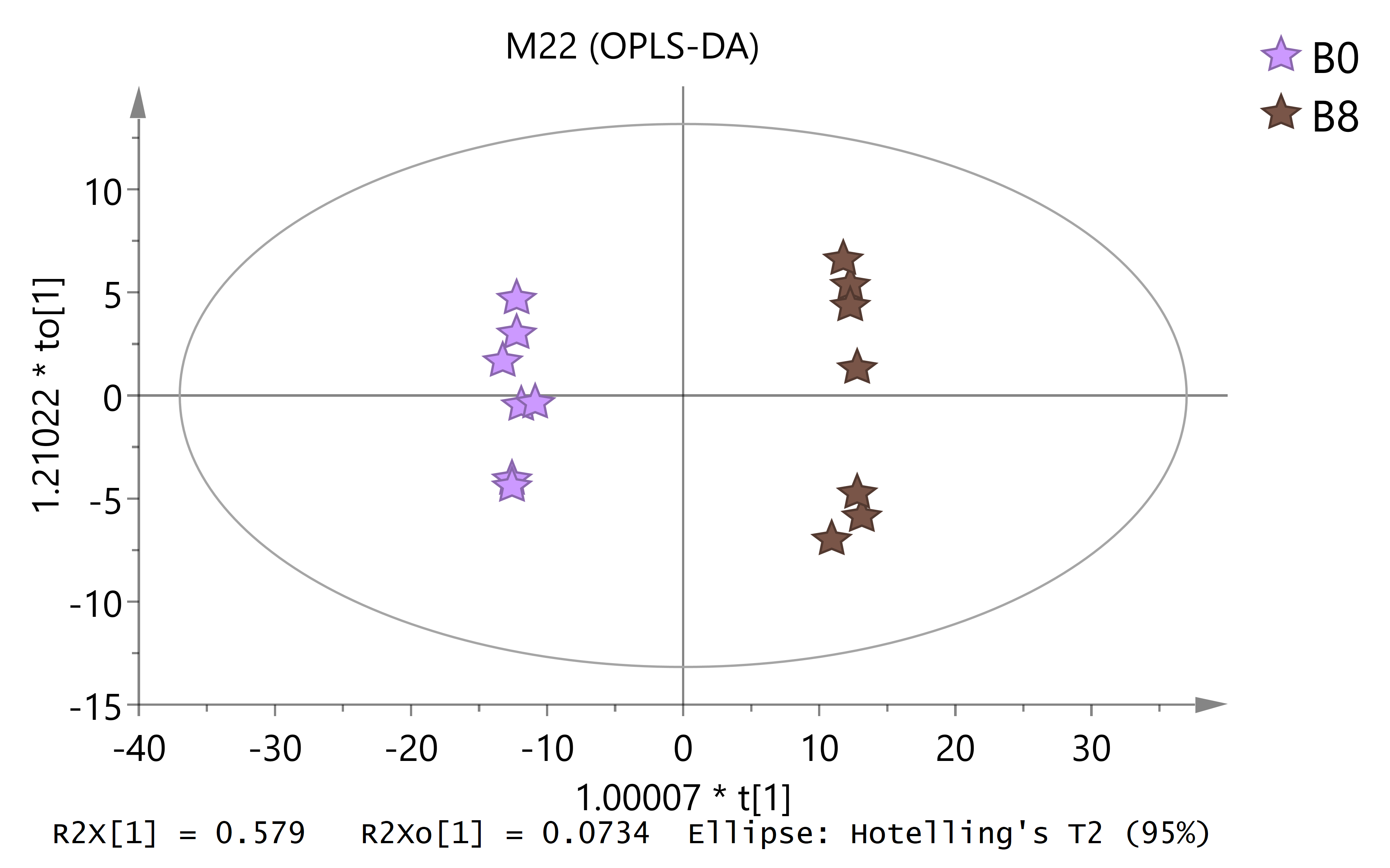

Supplement: Supplementary file 1 [file ijms-20-02330-s001.zip › supplementary material/2、Multivariate statistical analysis/opls(B0-8).tif]

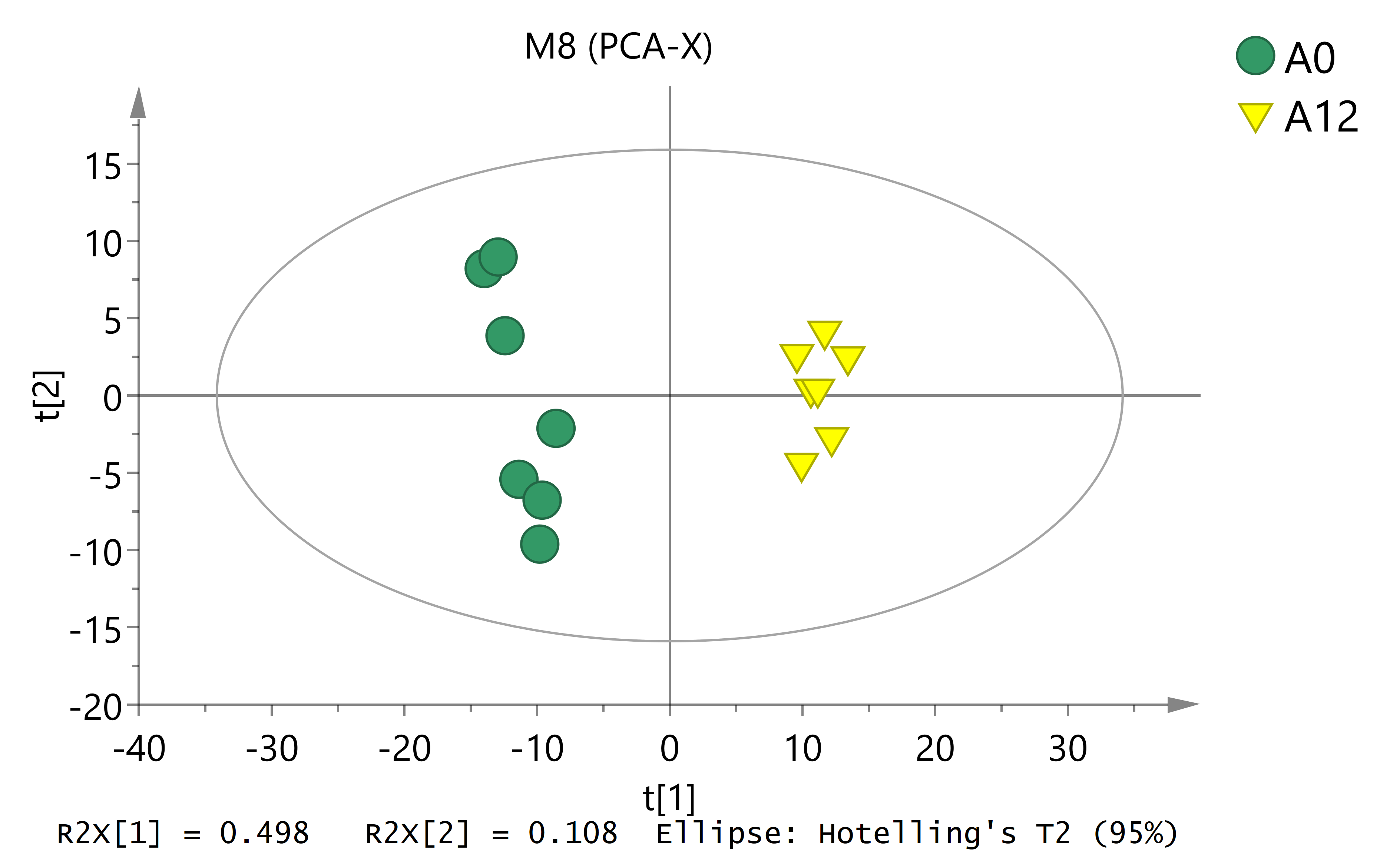

Supplement: Supplementary file 1 [file ijms-20-02330-s001.zip › supplementary material/2、Multivariate statistical analysis/pca(A0-12).tif]

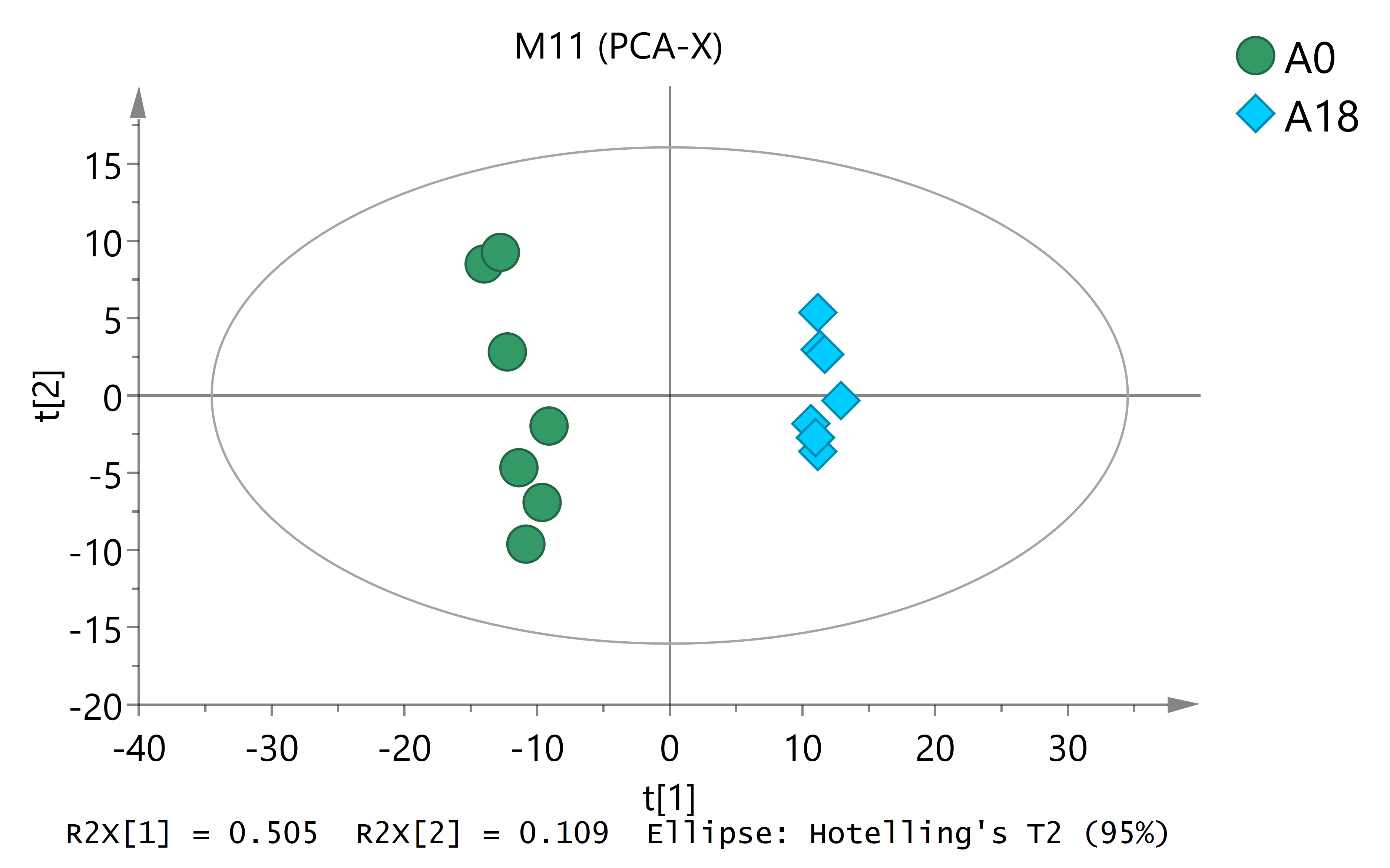

Supplement: Supplementary file 1 [file ijms-20-02330-s001.zip › supplementary material/2、Multivariate statistical analysis/pca(A0-18).tif]

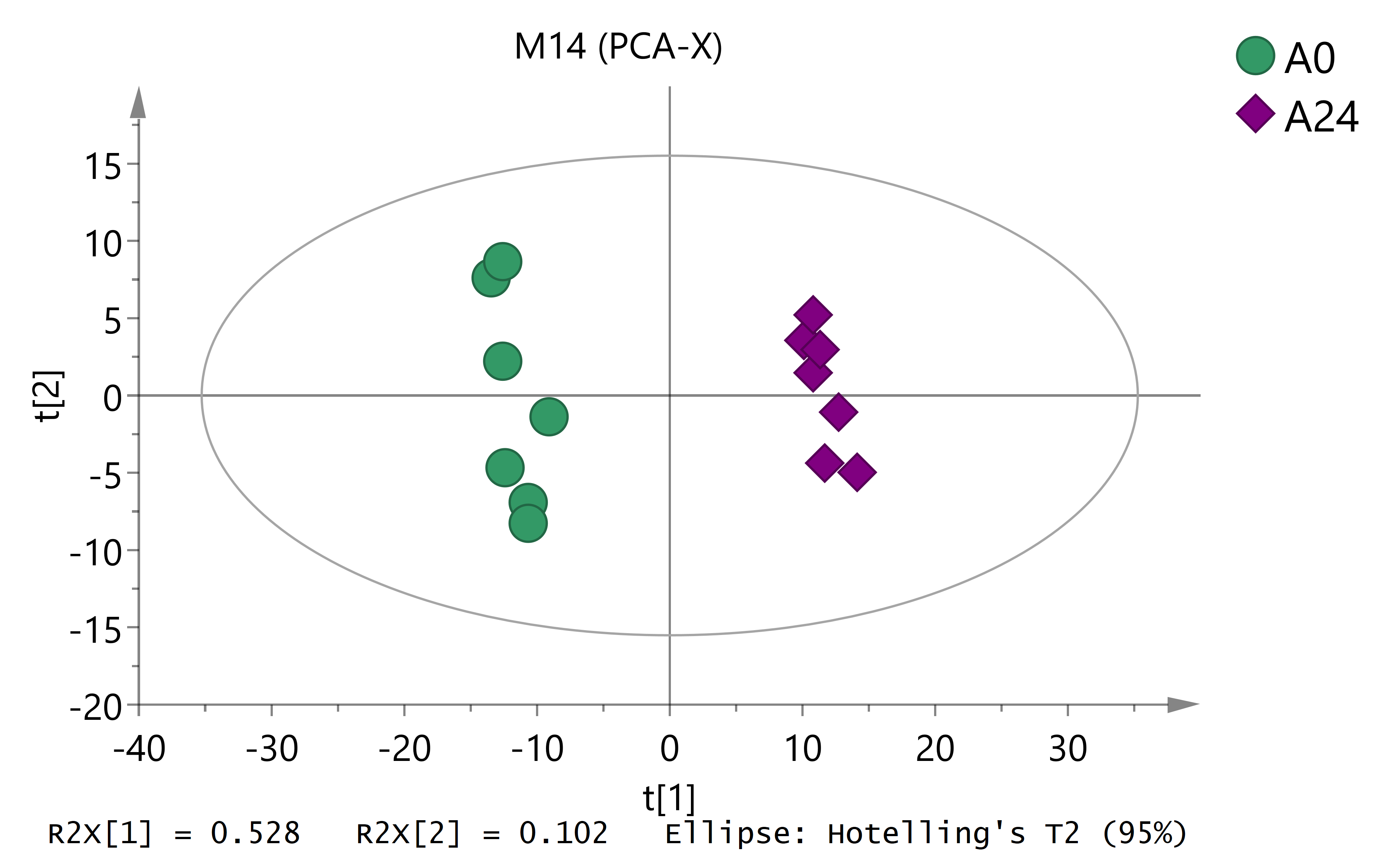

Supplement: Supplementary file 1 [file ijms-20-02330-s001.zip › supplementary material/2、Multivariate statistical analysis/pca(A0-24).tif]

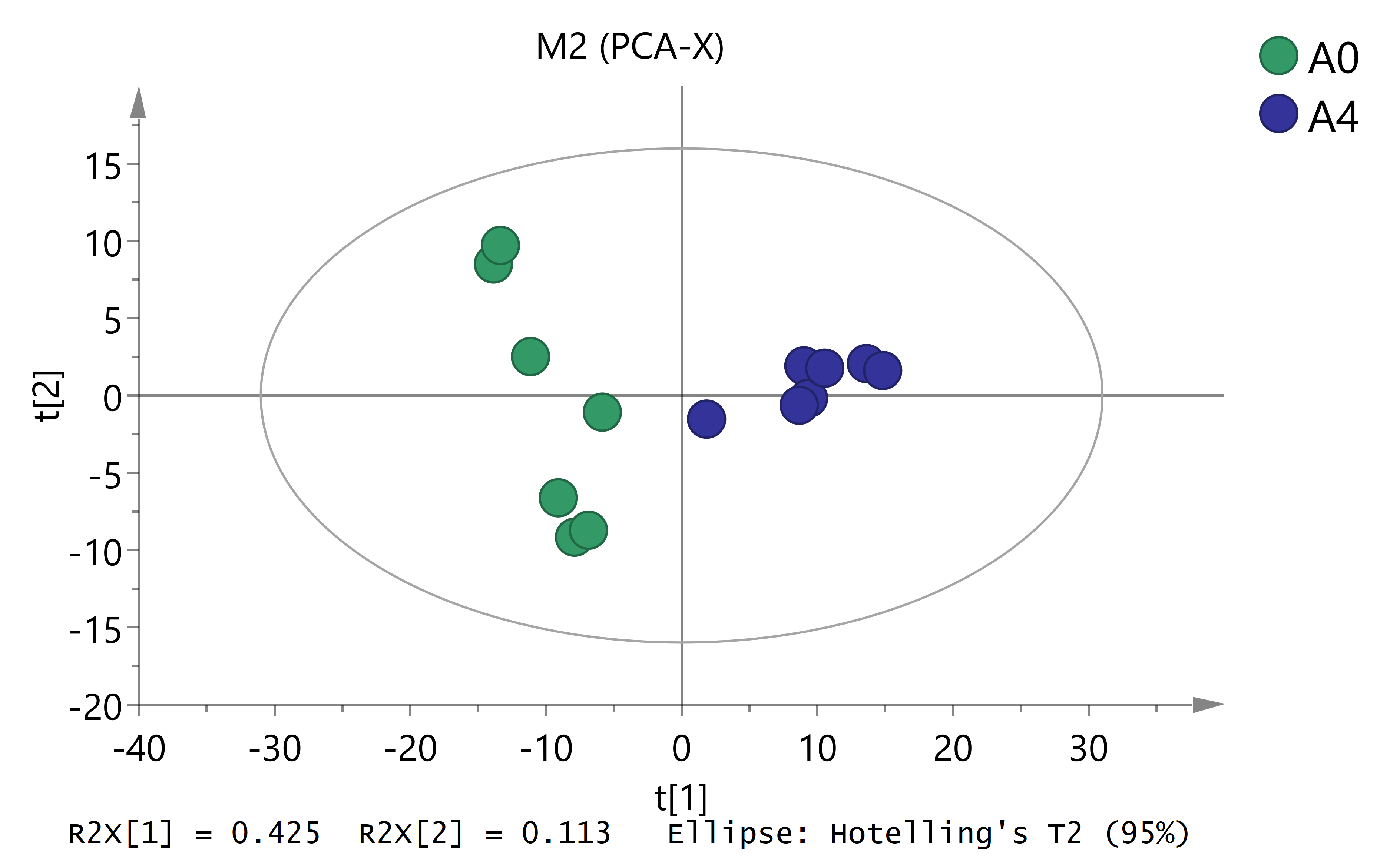

Supplement: Supplementary file 1 [file ijms-20-02330-s001.zip › supplementary material/2、Multivariate statistical analysis/pca(A0-4).tif]

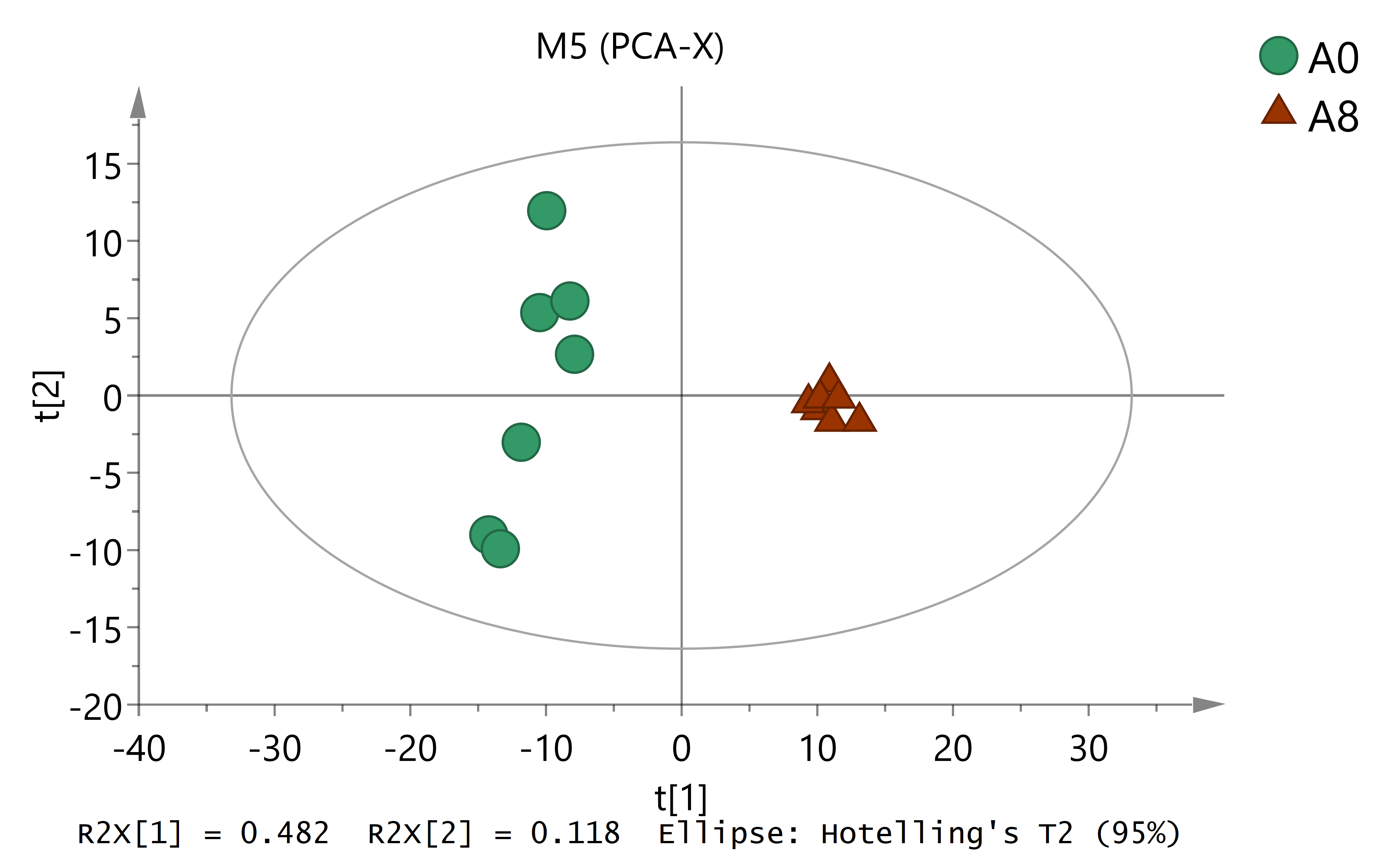

Supplement: Supplementary file 1 [file ijms-20-02330-s001.zip › supplementary material/2、Multivariate statistical analysis/pca(A0-8).tif]

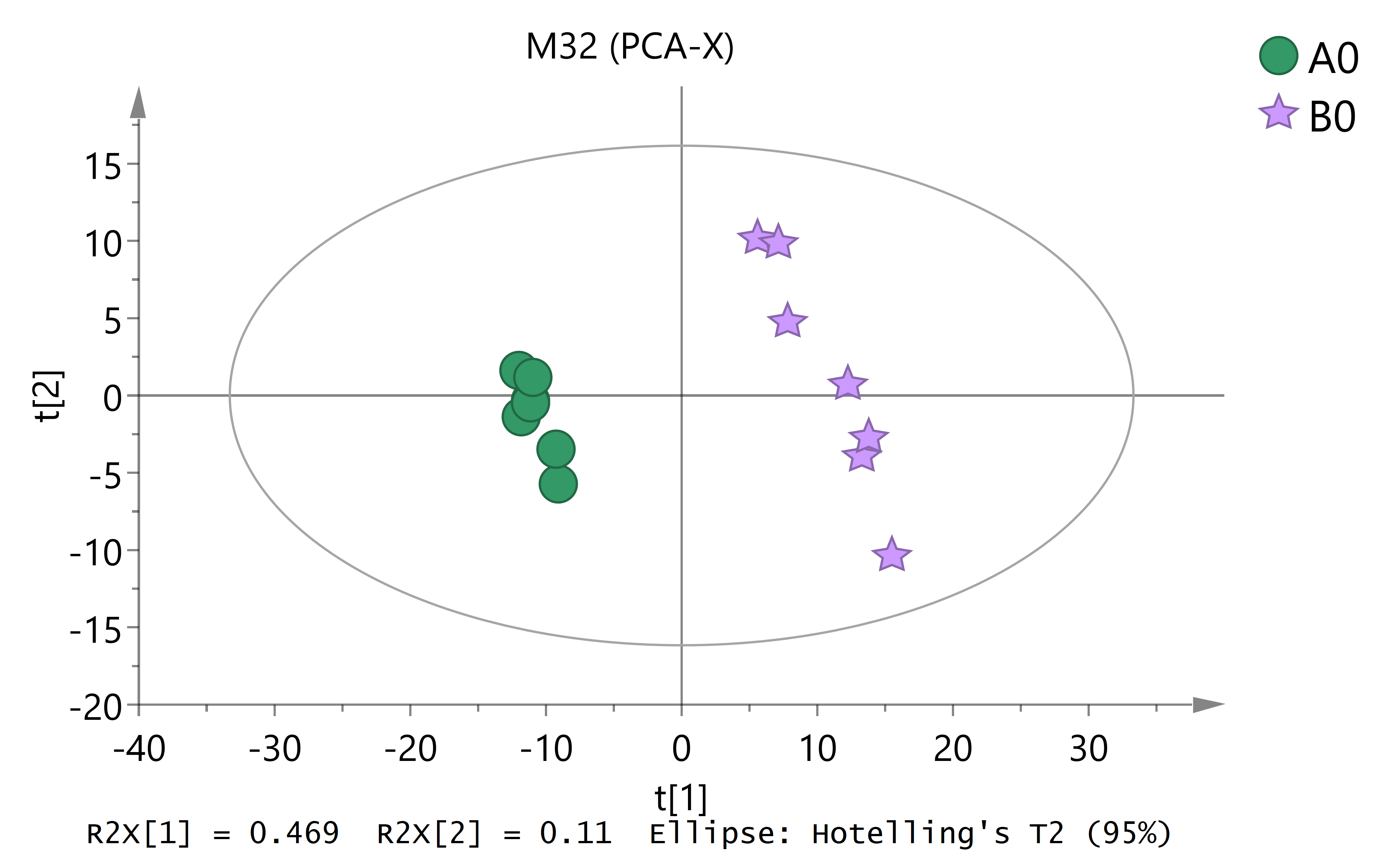

Supplement: Supplementary file 1 [file ijms-20-02330-s001.zip › supplementary material/2、Multivariate statistical analysis/pca(A0-B0).tif]

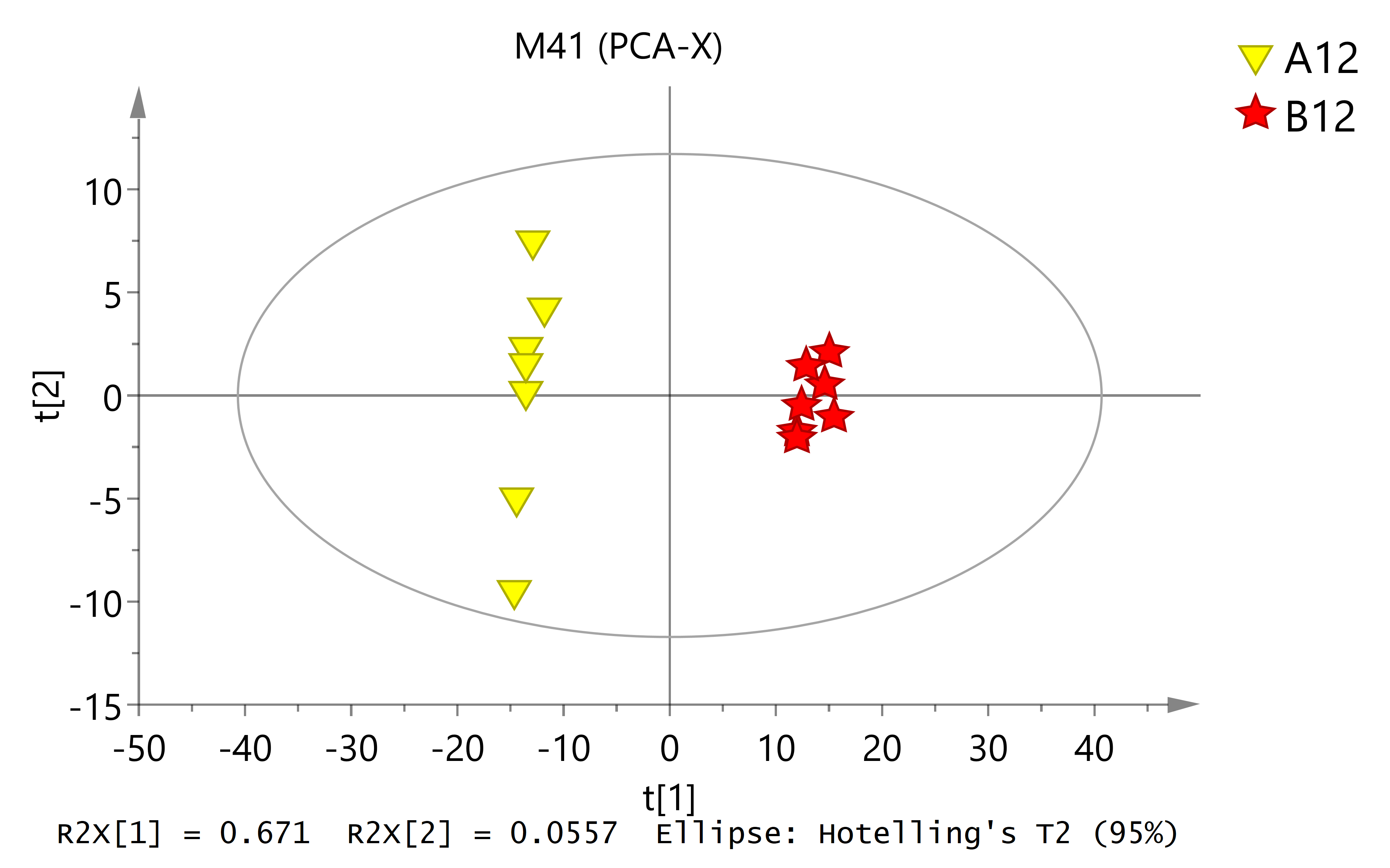

Supplement: Supplementary file 1 [file ijms-20-02330-s001.zip › supplementary material/2、Multivariate statistical analysis/pca(A12-B12).tif]

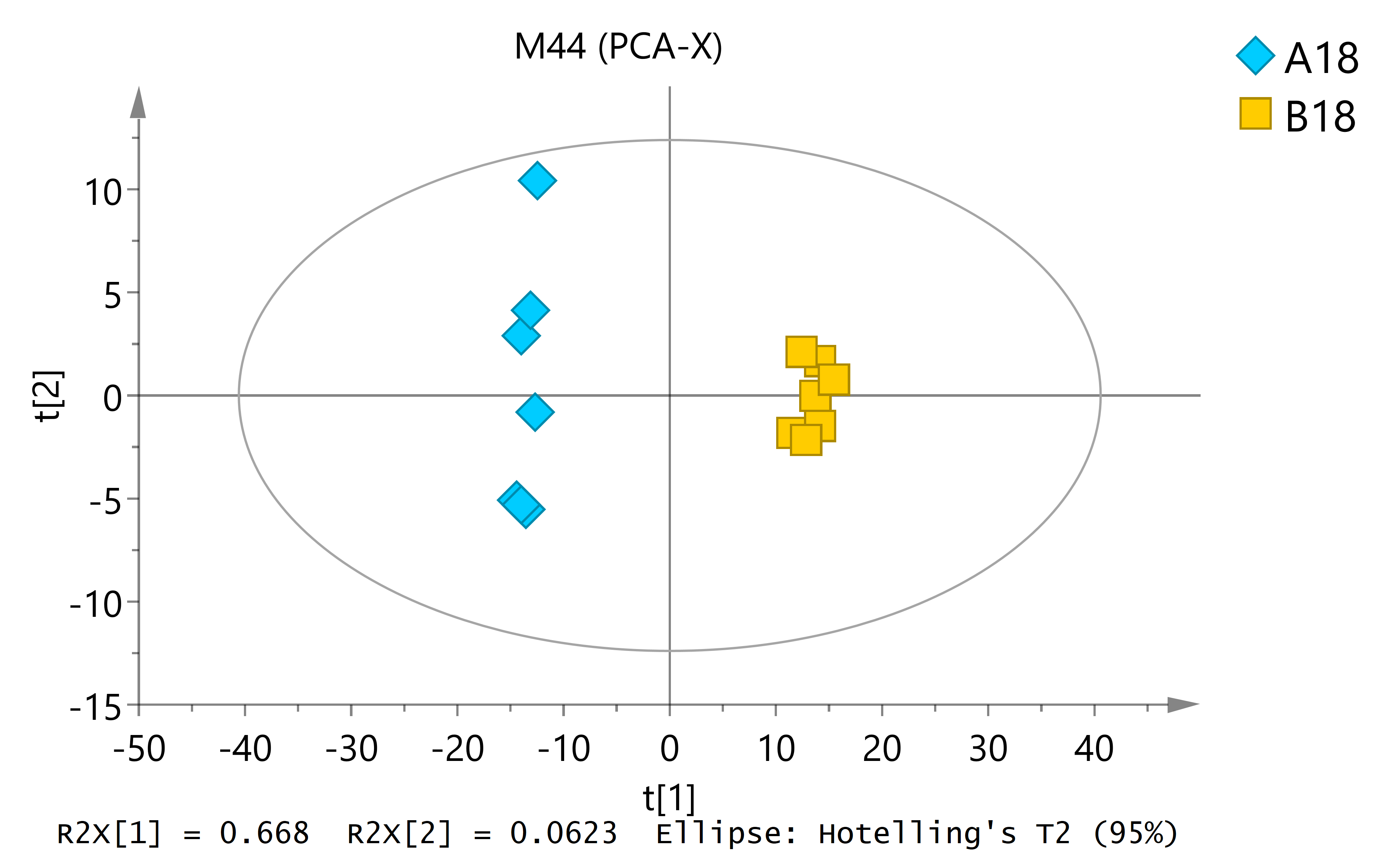

Supplement: Supplementary file 1 [file ijms-20-02330-s001.zip › supplementary material/2、Multivariate statistical analysis/pca(A18-B18).tif]

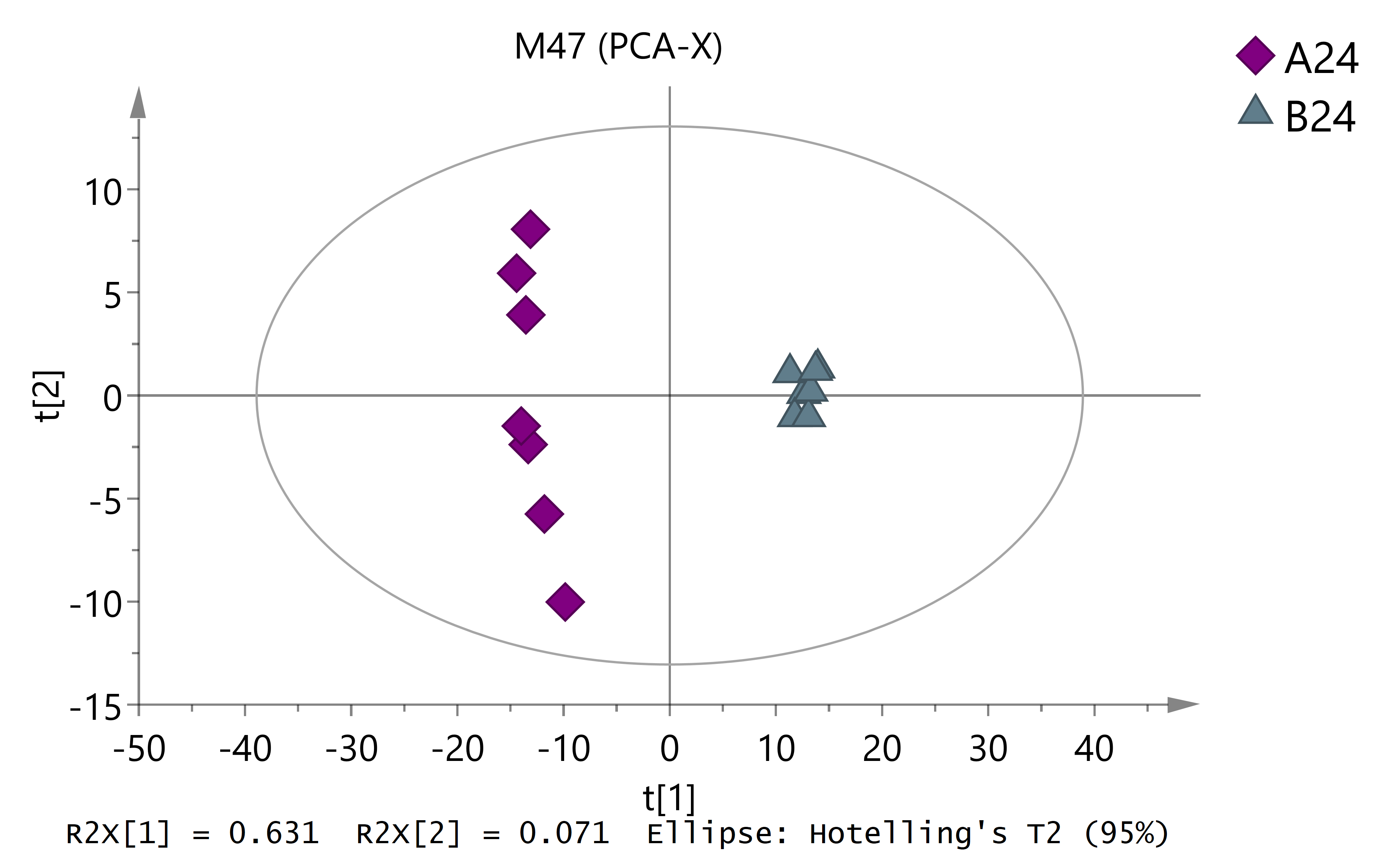

Supplement: Supplementary file 1 [file ijms-20-02330-s001.zip › supplementary material/2、Multivariate statistical analysis/pca(A24-B24).tif]

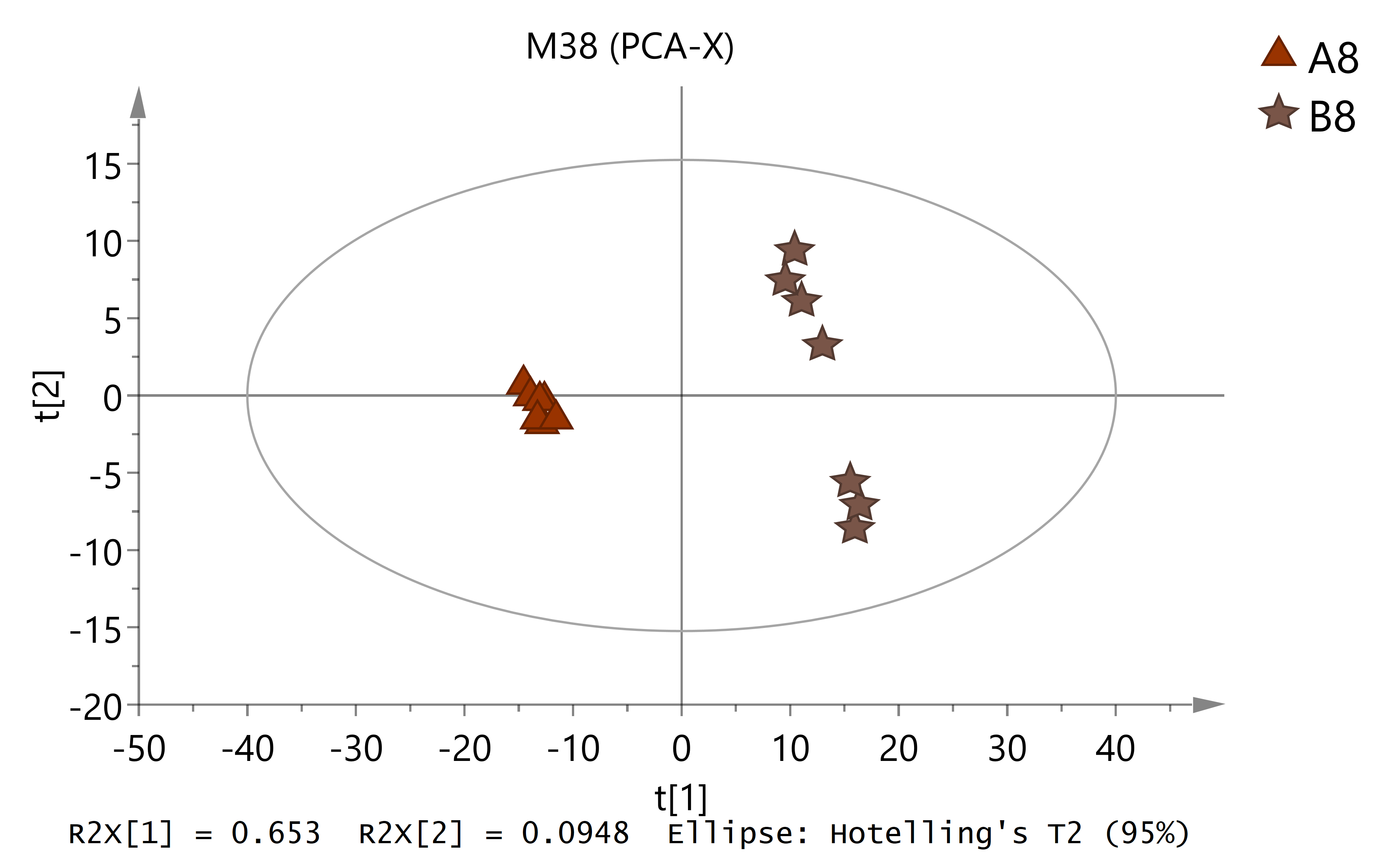

Supplement: Supplementary file 1 [file ijms-20-02330-s001.zip › supplementary material/2、Multivariate statistical analysis/pca(A8-B8).tif]

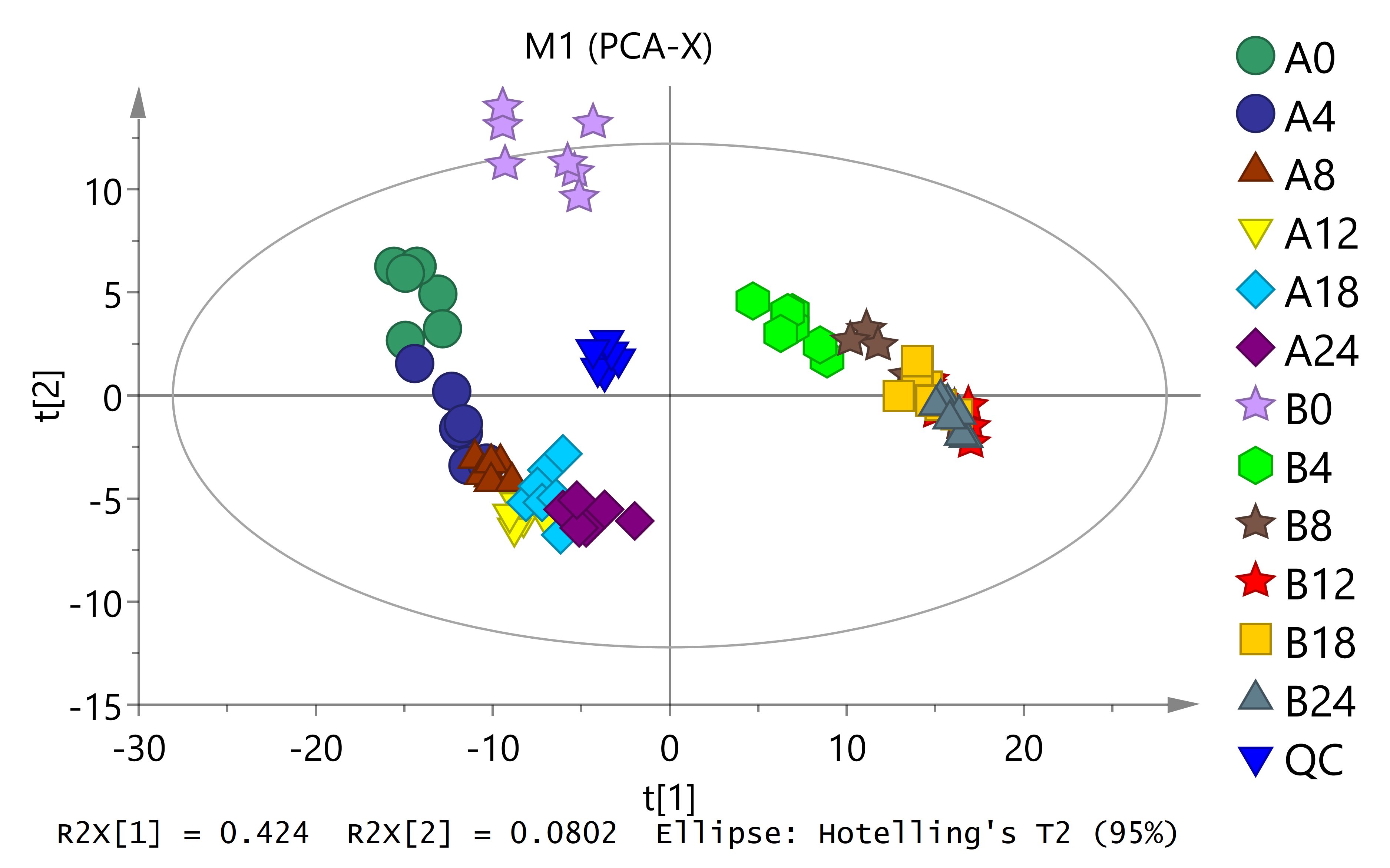

Supplement: Supplementary file 1 [file ijms-20-02330-s001.zip › supplementary material/2、Multivariate statistical analysis/pca(all).tif]

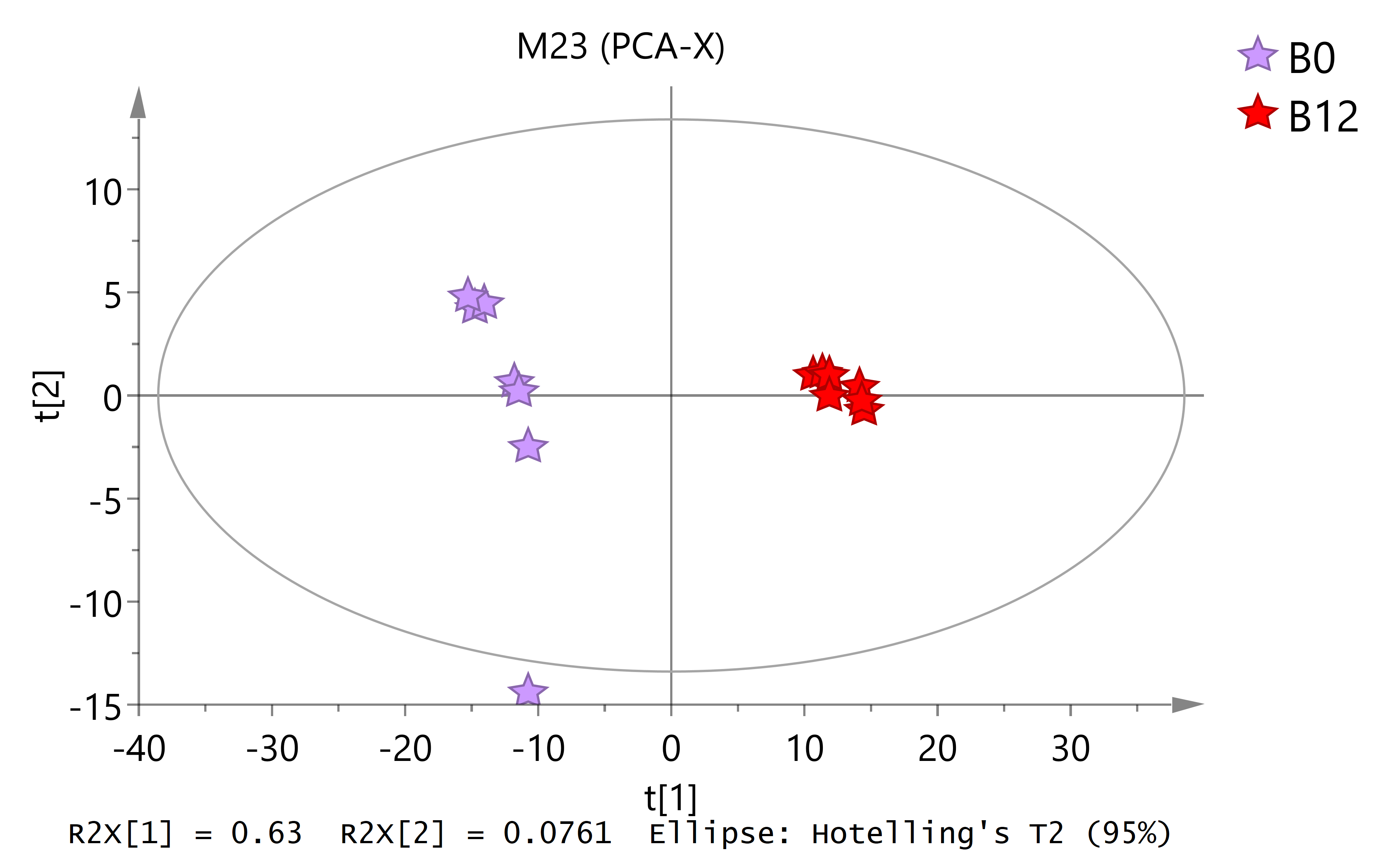

Supplement: Supplementary file 1 [file ijms-20-02330-s001.zip › supplementary material/2、Multivariate statistical analysis/pca(B0-12).tif]

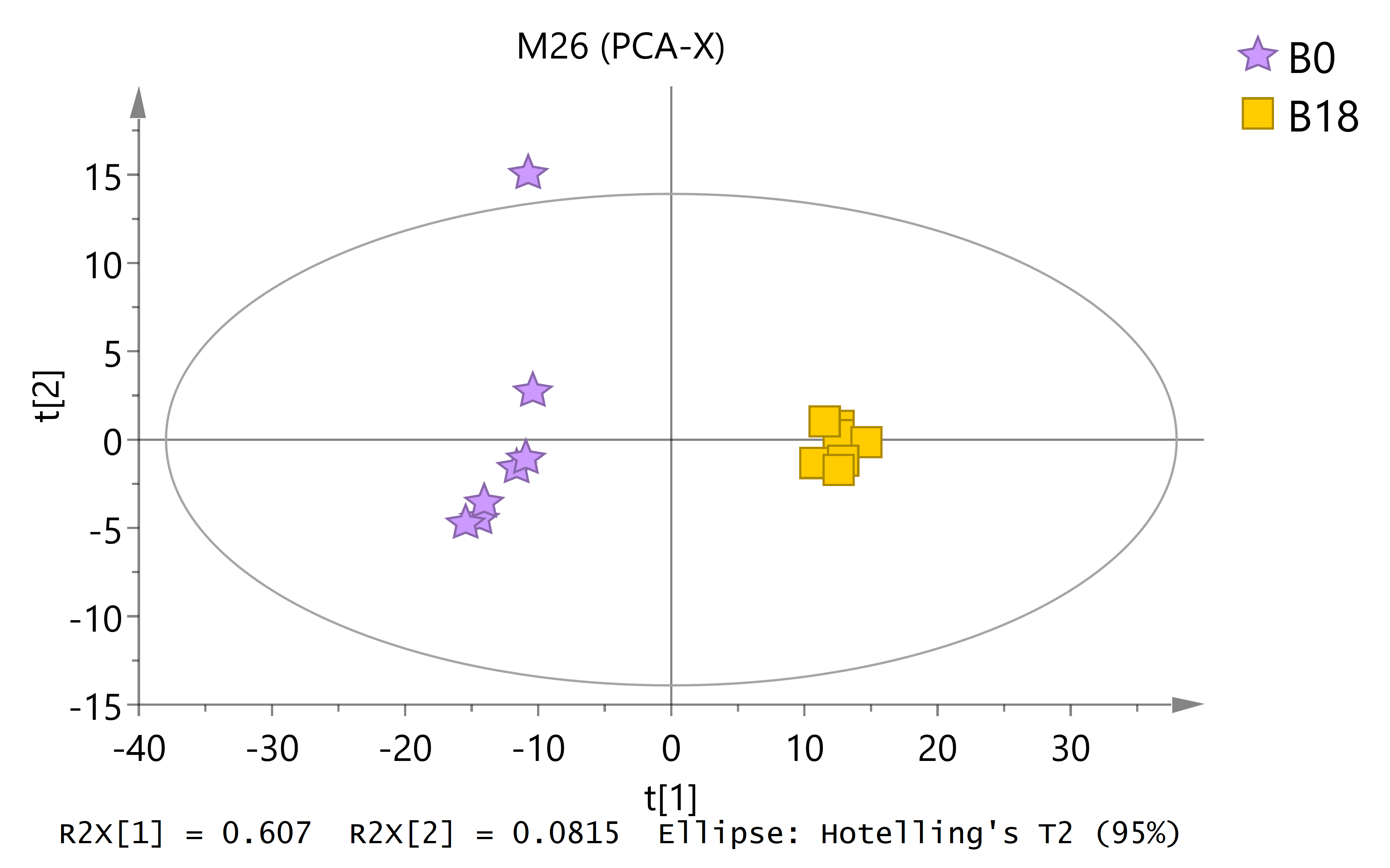

Supplement: Supplementary file 1 [file ijms-20-02330-s001.zip › supplementary material/2、Multivariate statistical analysis/pca(B0-18).tif]

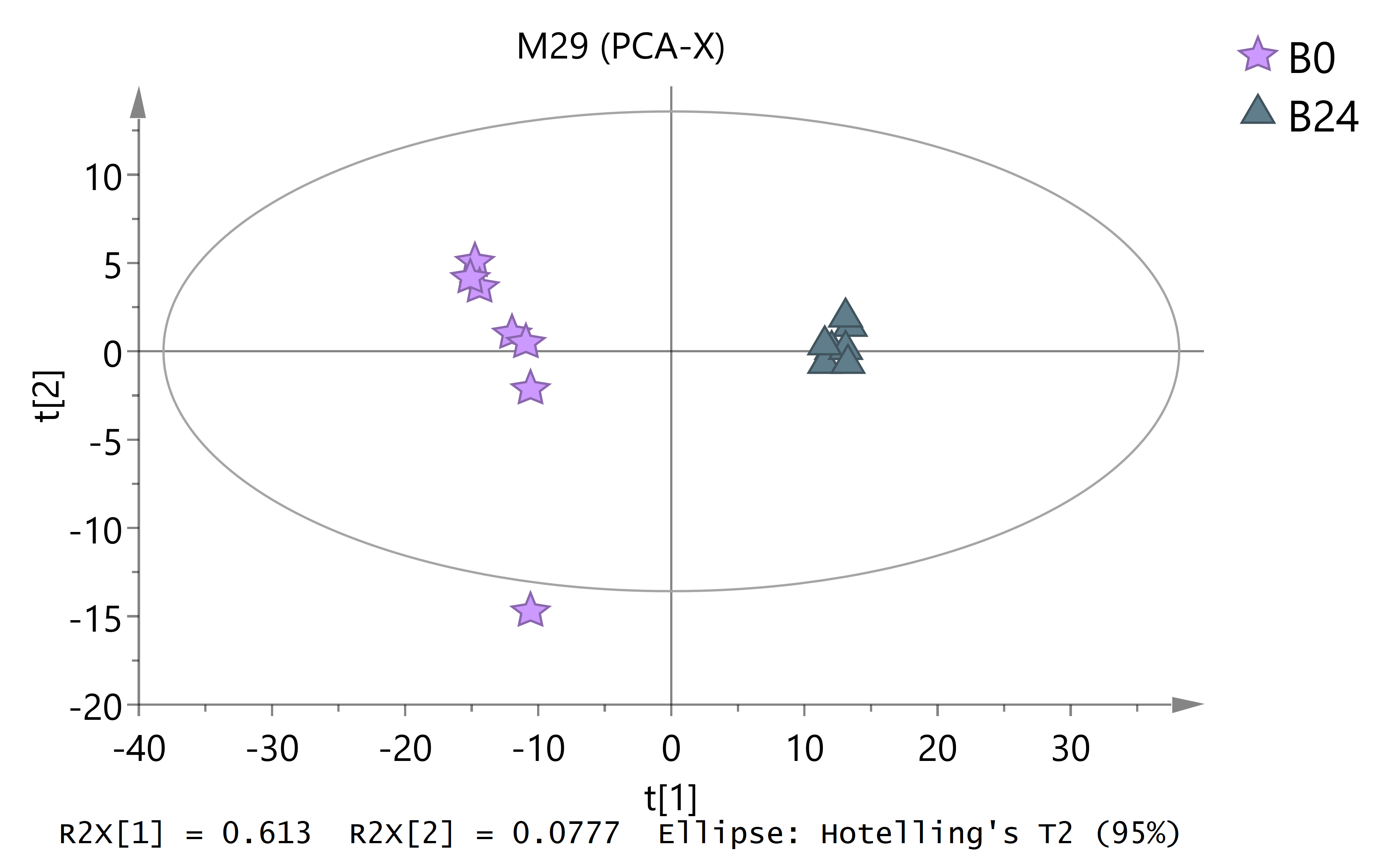

Supplement: Supplementary file 1 [file ijms-20-02330-s001.zip › supplementary material/2、Multivariate statistical analysis/pca(B0-24).tif]

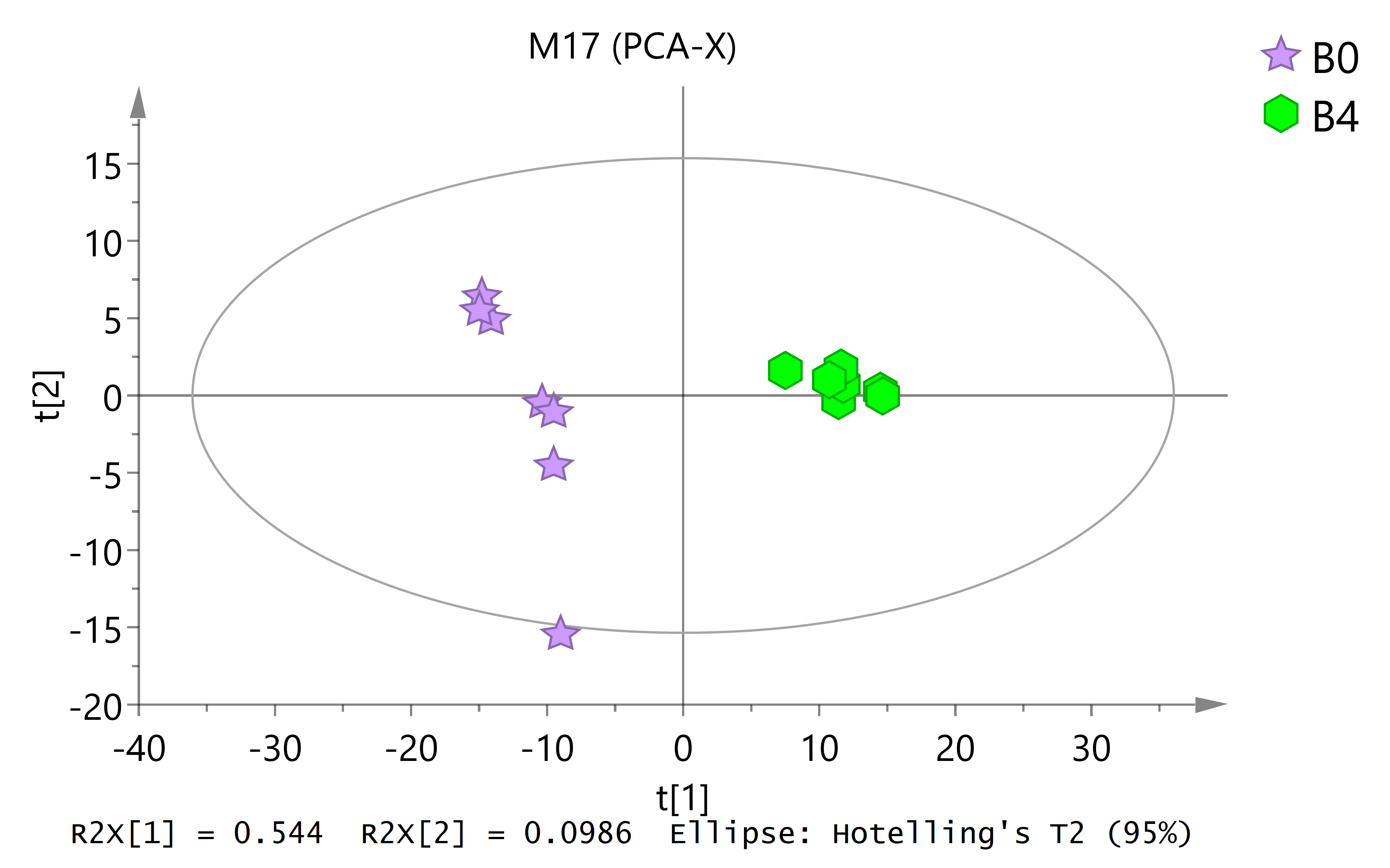

Supplement: Supplementary file 1 [file ijms-20-02330-s001.zip › supplementary material/2、Multivariate statistical analysis/pca(B0-4).tif]

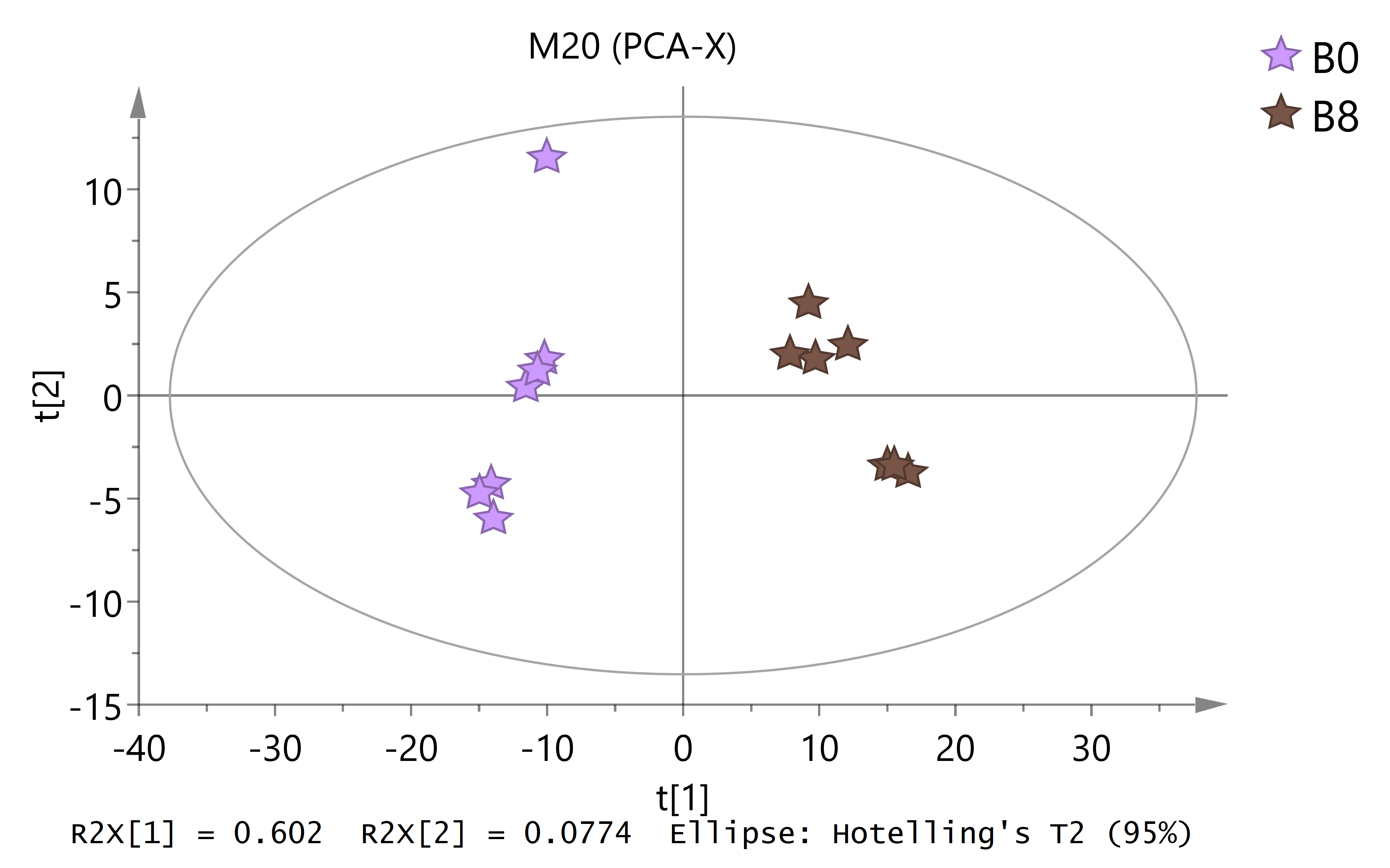

Supplement: Supplementary file 1 [file ijms-20-02330-s001.zip › supplementary material/2、Multivariate statistical analysis/pca(B0-8).tif]

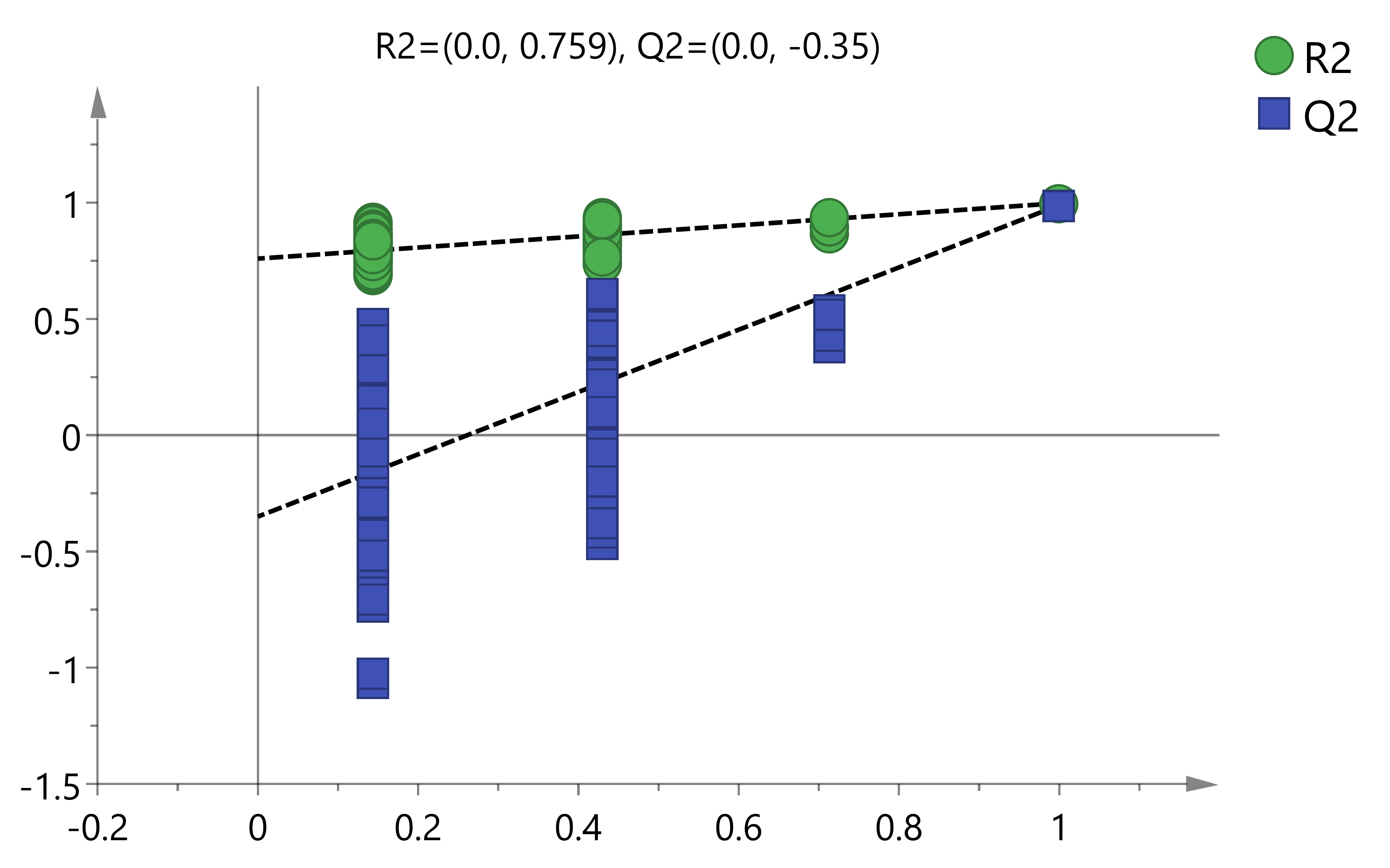

Supplement: Supplementary file 1 [file ijms-20-02330-s001.zip › supplementary material/2、Multivariate statistical analysis/Permutation(0-12).tif]

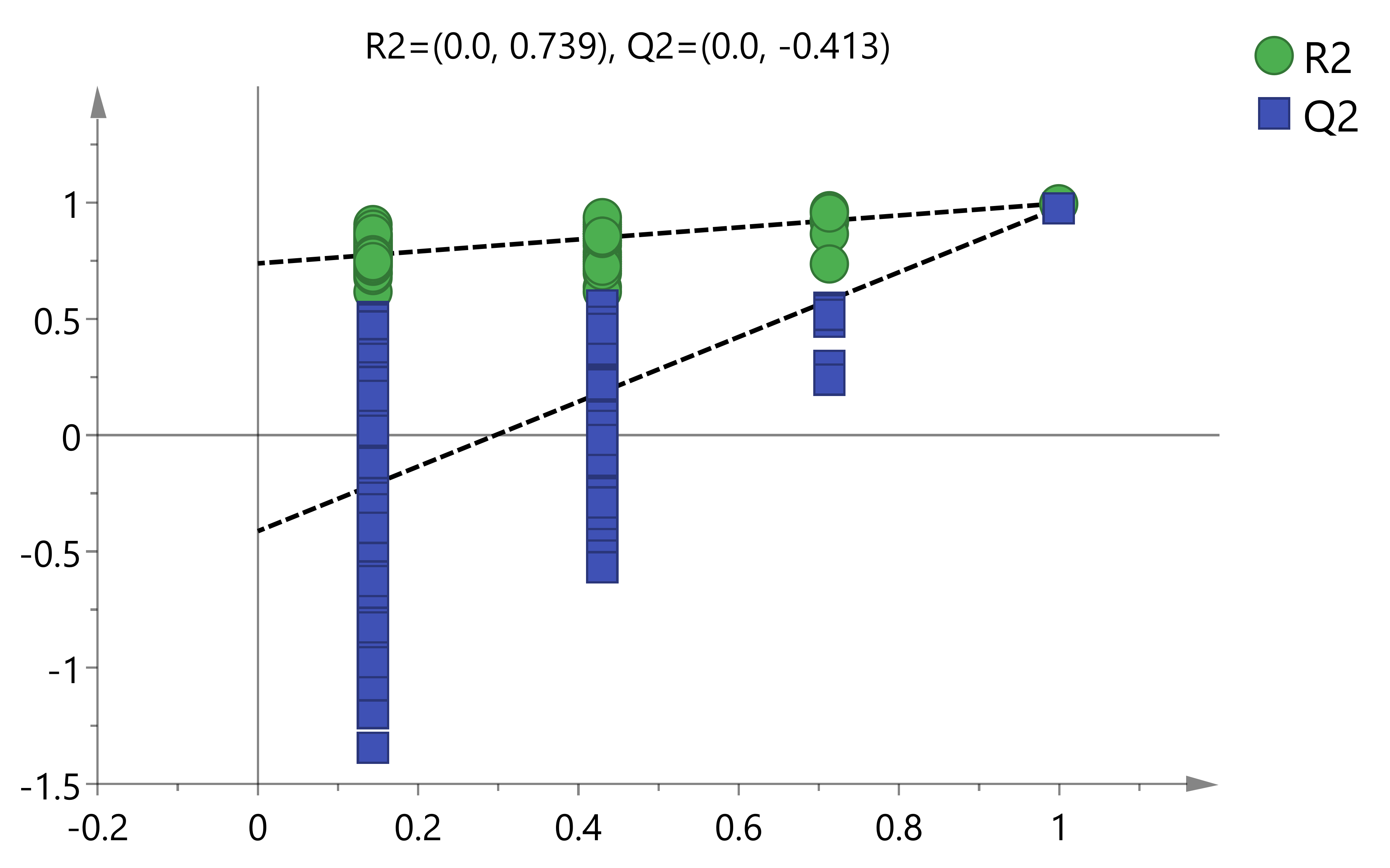

Supplement: Supplementary file 1 [file ijms-20-02330-s001.zip › supplementary material/2、Multivariate statistical analysis/Permutation(A0-12).tif]

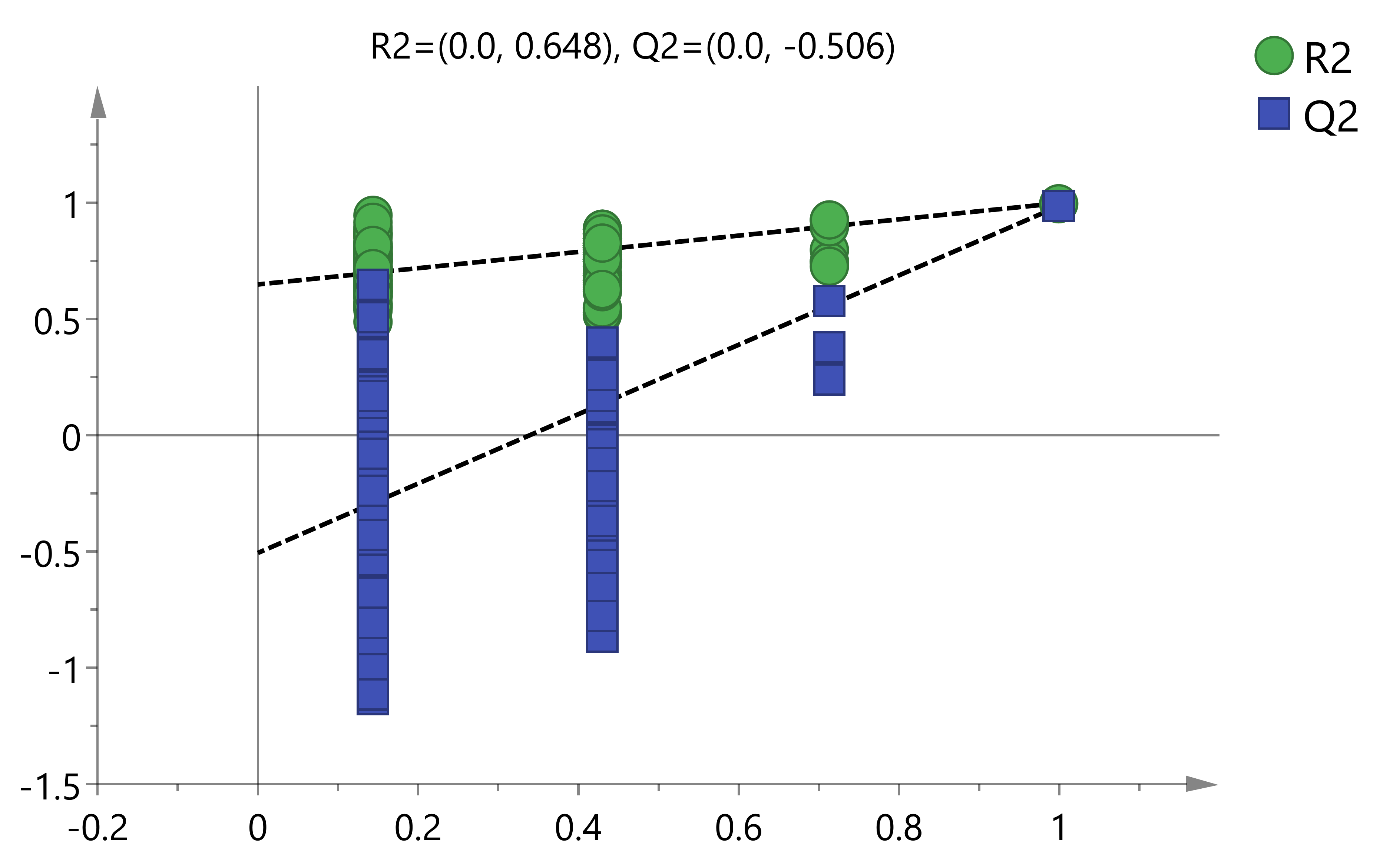

Supplement: Supplementary file 1 [file ijms-20-02330-s001.zip › supplementary material/2、Multivariate statistical analysis/Permutation(A0-18).tif]

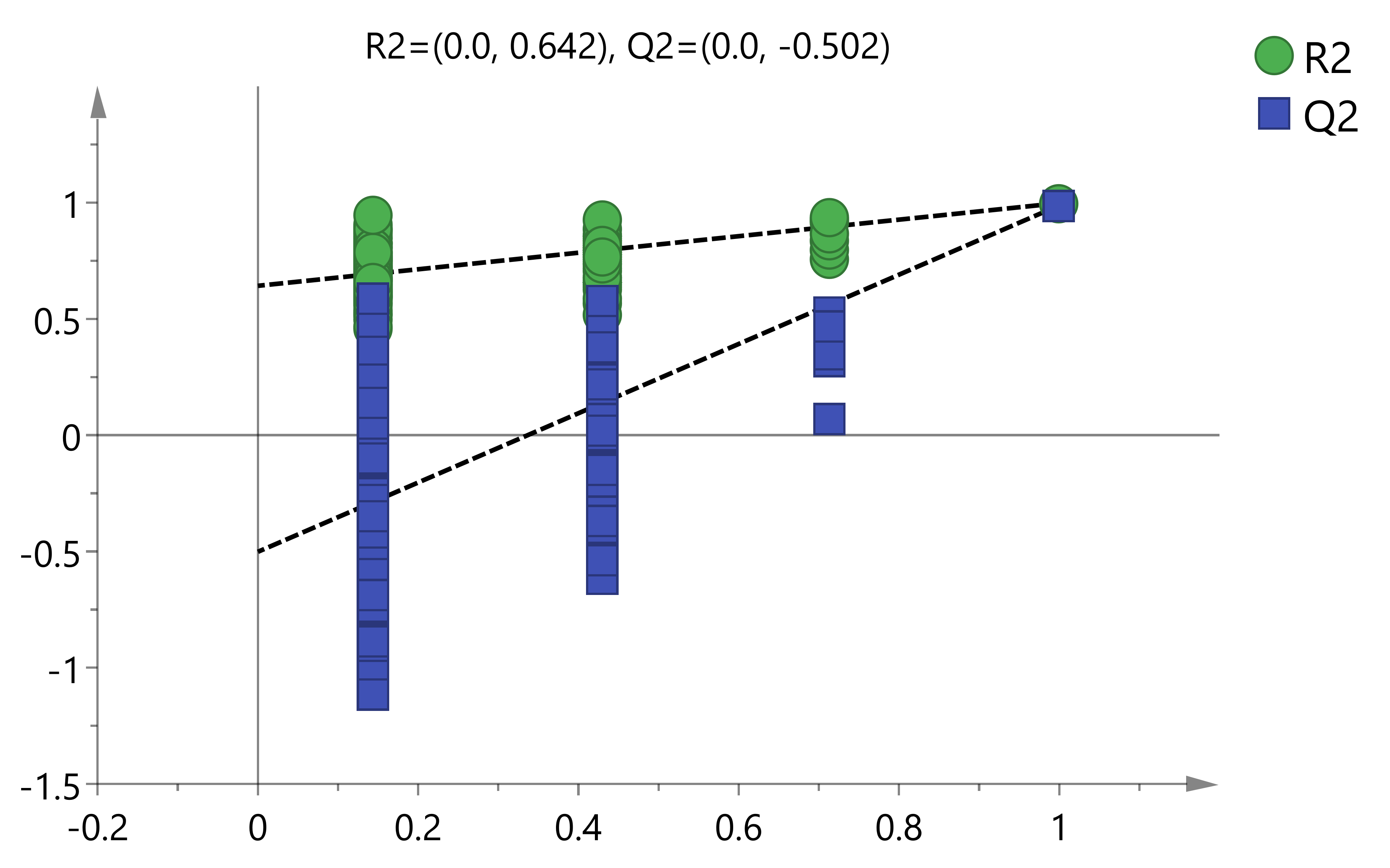

Supplement: Supplementary file 1 [file ijms-20-02330-s001.zip › supplementary material/2、Multivariate statistical analysis/Permutation(A0-24).tif]

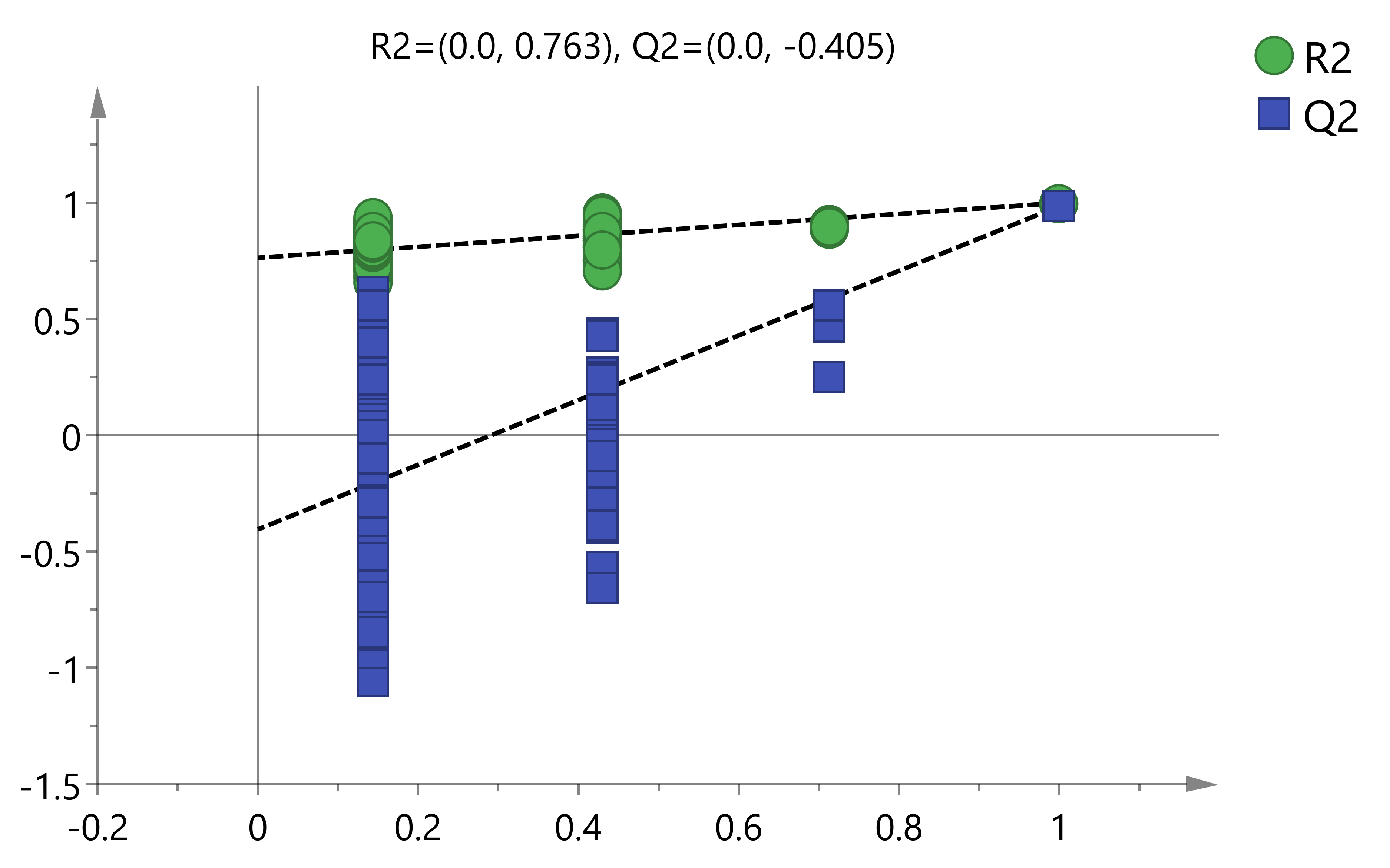

Supplement: Supplementary file 1 [file ijms-20-02330-s001.zip › supplementary material/2、Multivariate statistical analysis/Permutation(A0-B0).tif]

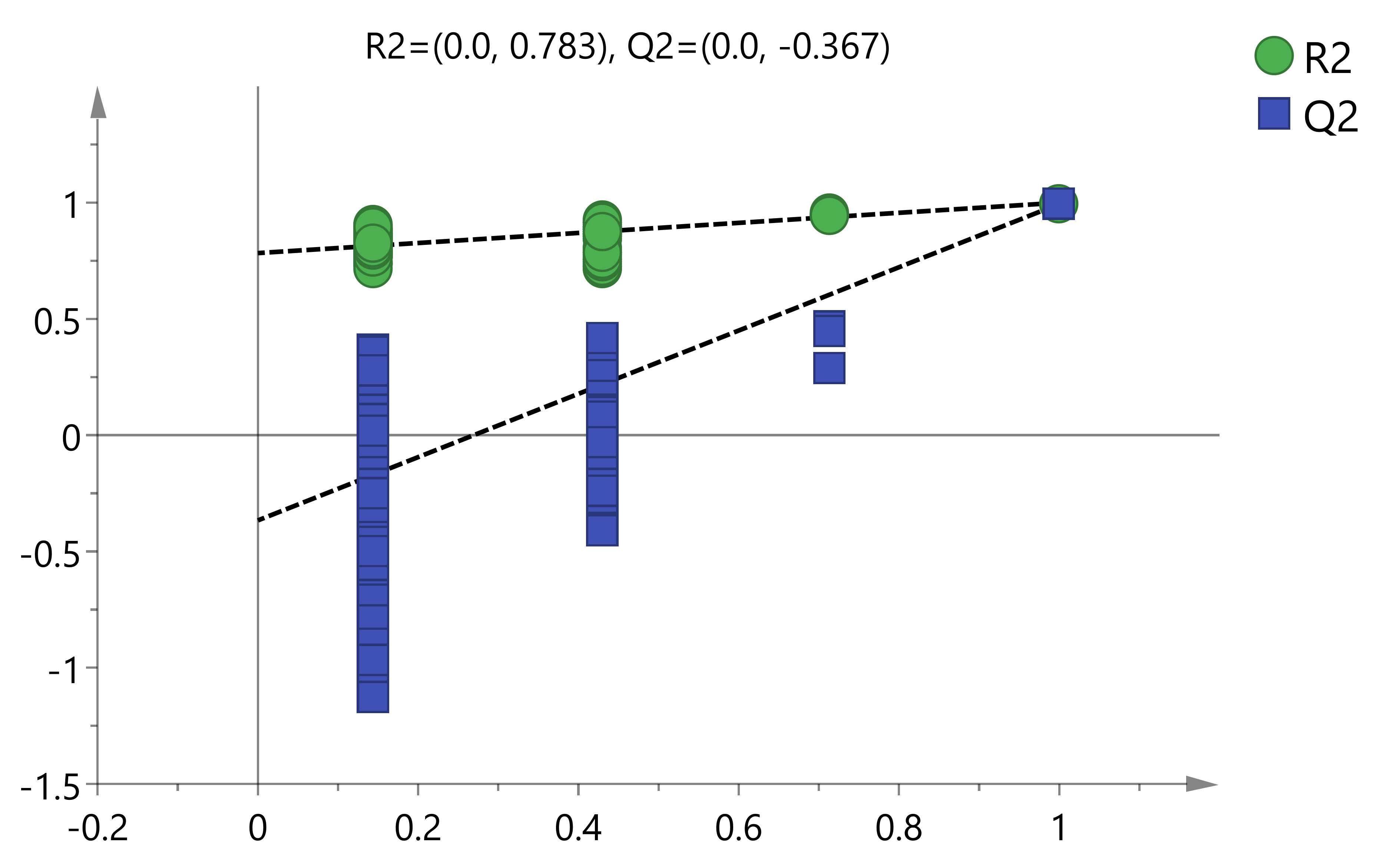

Supplement: Supplementary file 1 [file ijms-20-02330-s001.zip › supplementary material/2、Multivariate statistical analysis/Permutation(A12-B12).tif]

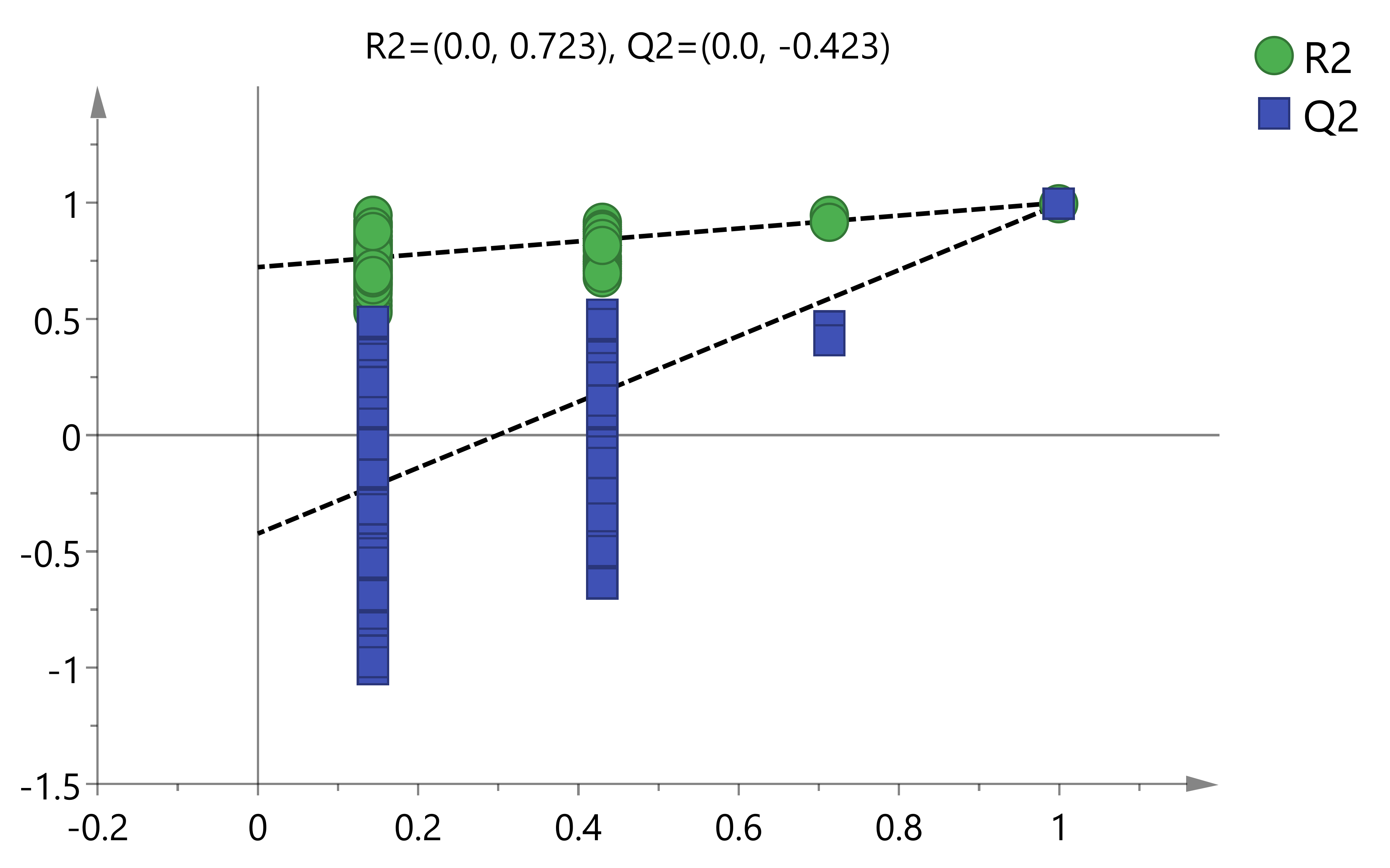

Supplement: Supplementary file 1 [file ijms-20-02330-s001.zip › supplementary material/2、Multivariate statistical analysis/Permutation(A18-B18).tif]

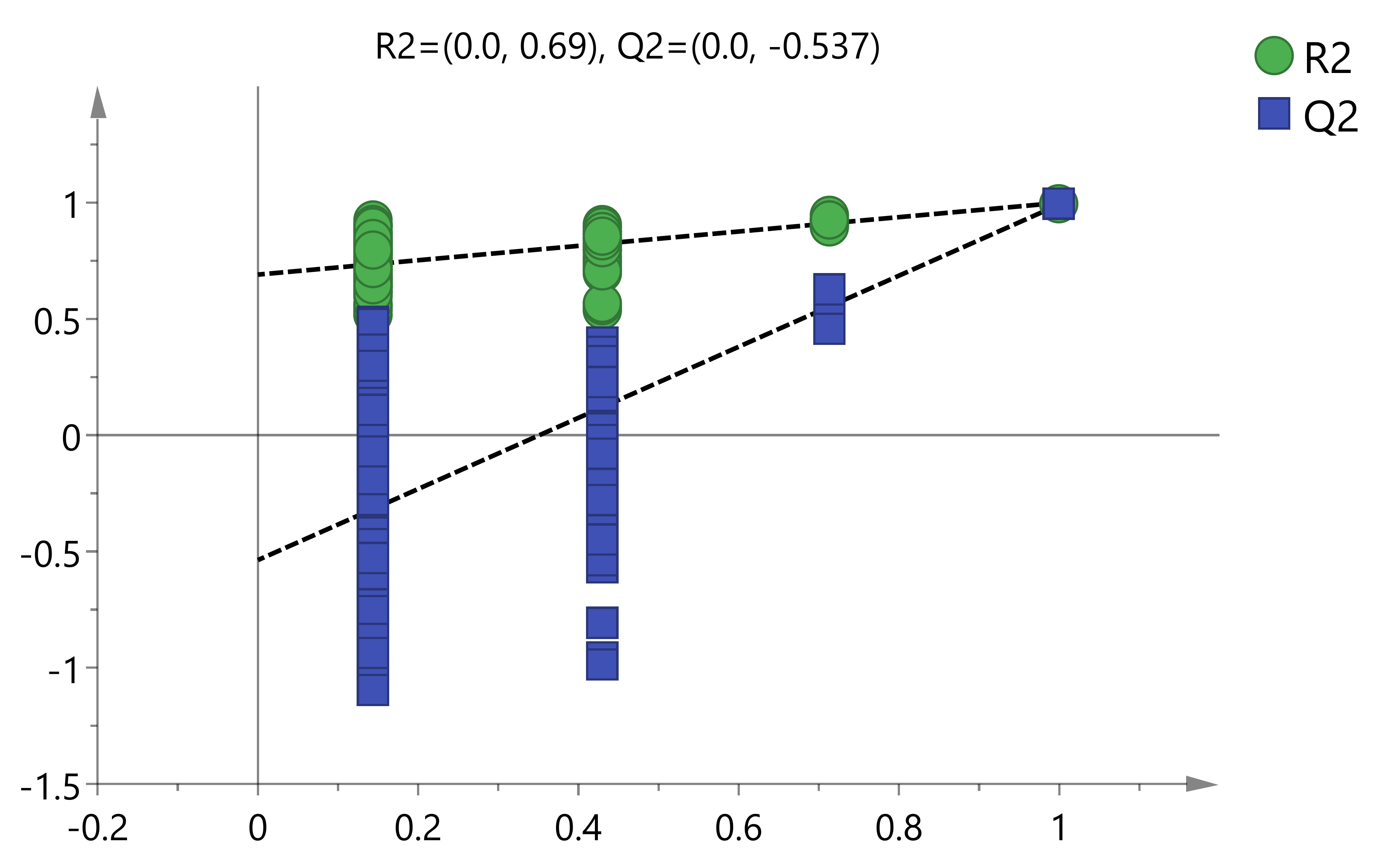

Supplement: Supplementary file 1 [file ijms-20-02330-s001.zip › supplementary material/2、Multivariate statistical analysis/Permutation(A24-B24).tif]

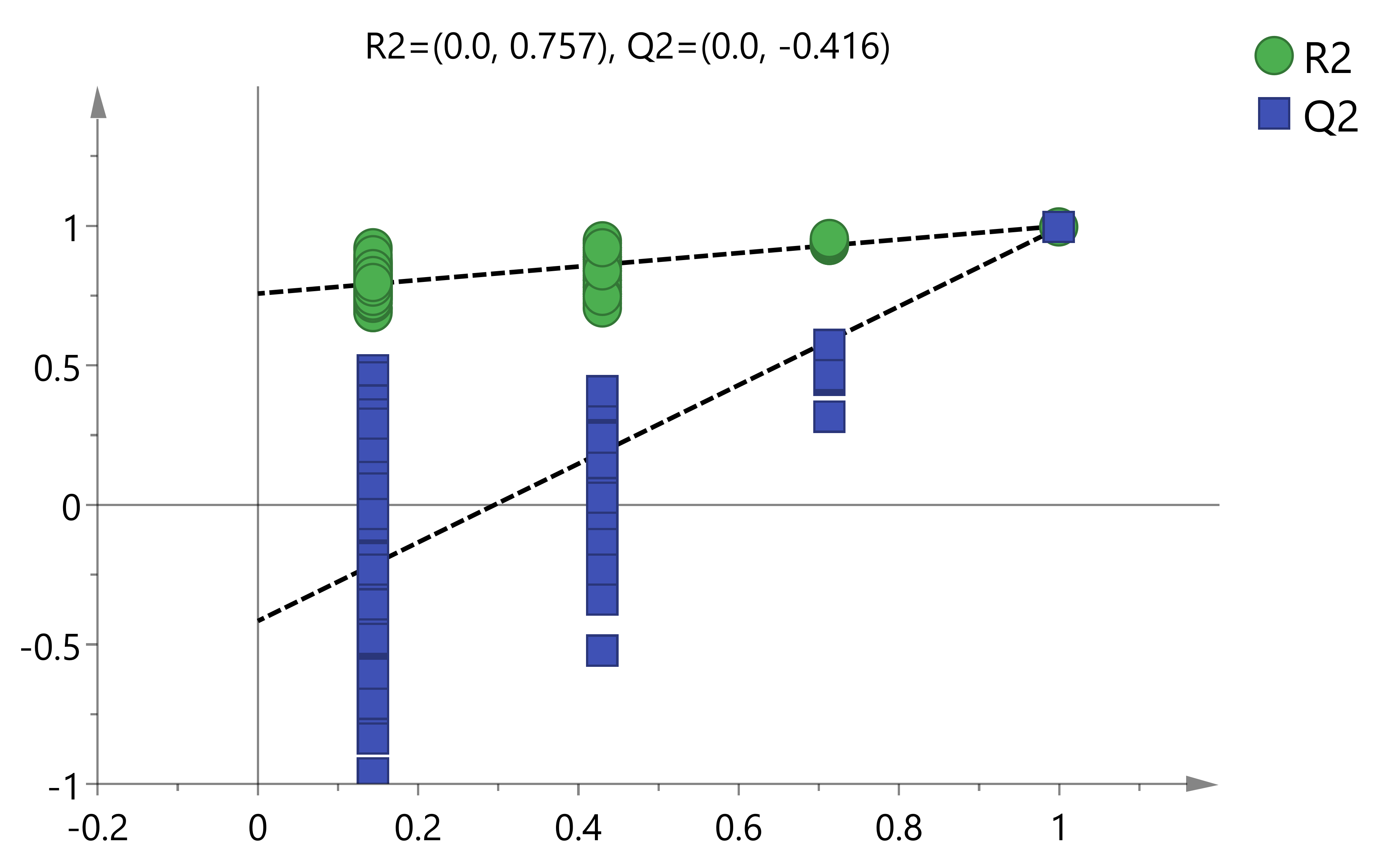

Supplement: Supplementary file 1 [file ijms-20-02330-s001.zip › supplementary material/2、Multivariate statistical analysis/Permutation(A4-B4).tif]

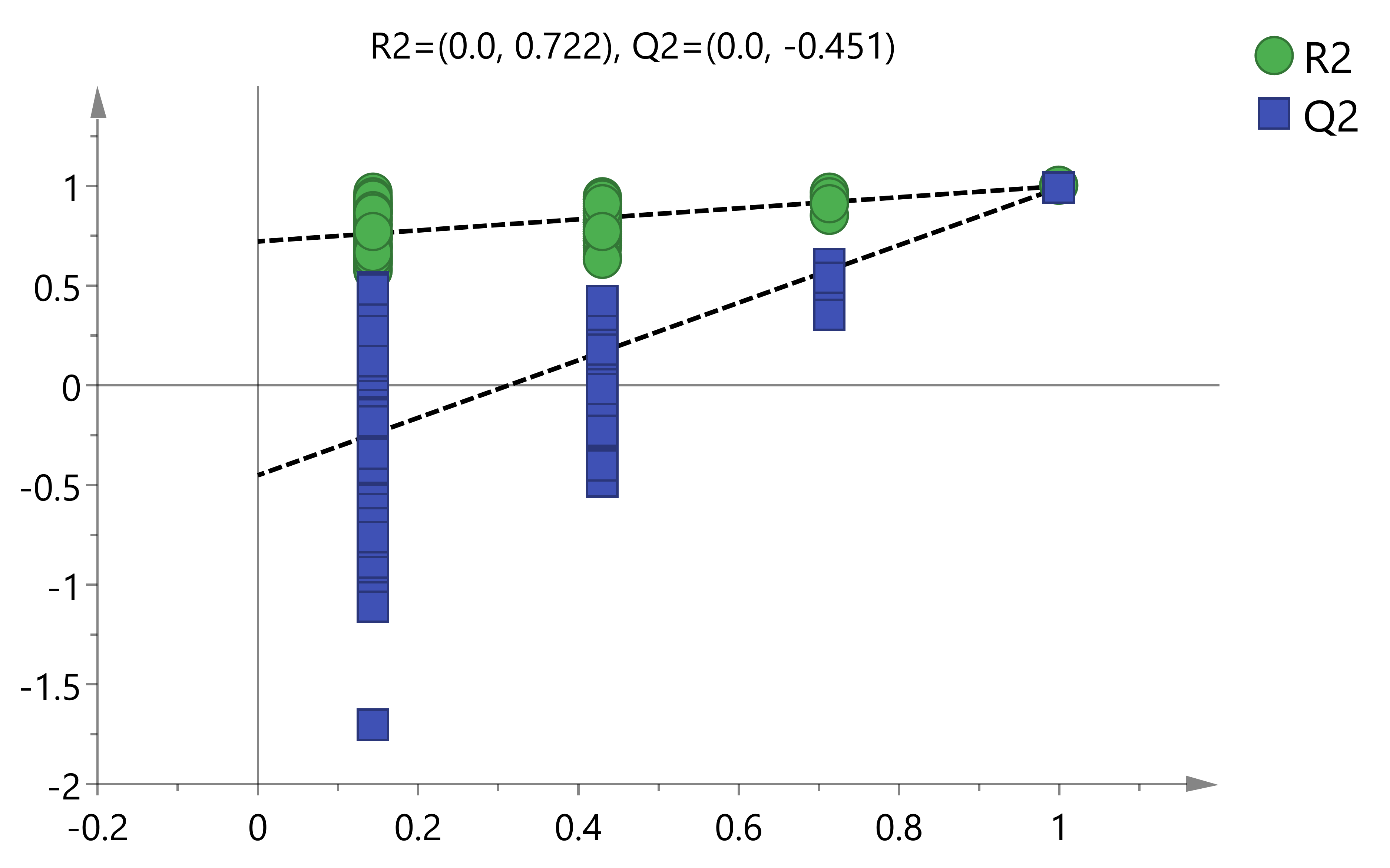

Supplement: Supplementary file 1 [file ijms-20-02330-s001.zip › supplementary material/2、Multivariate statistical analysis/Permutation(A8-B8).tif]

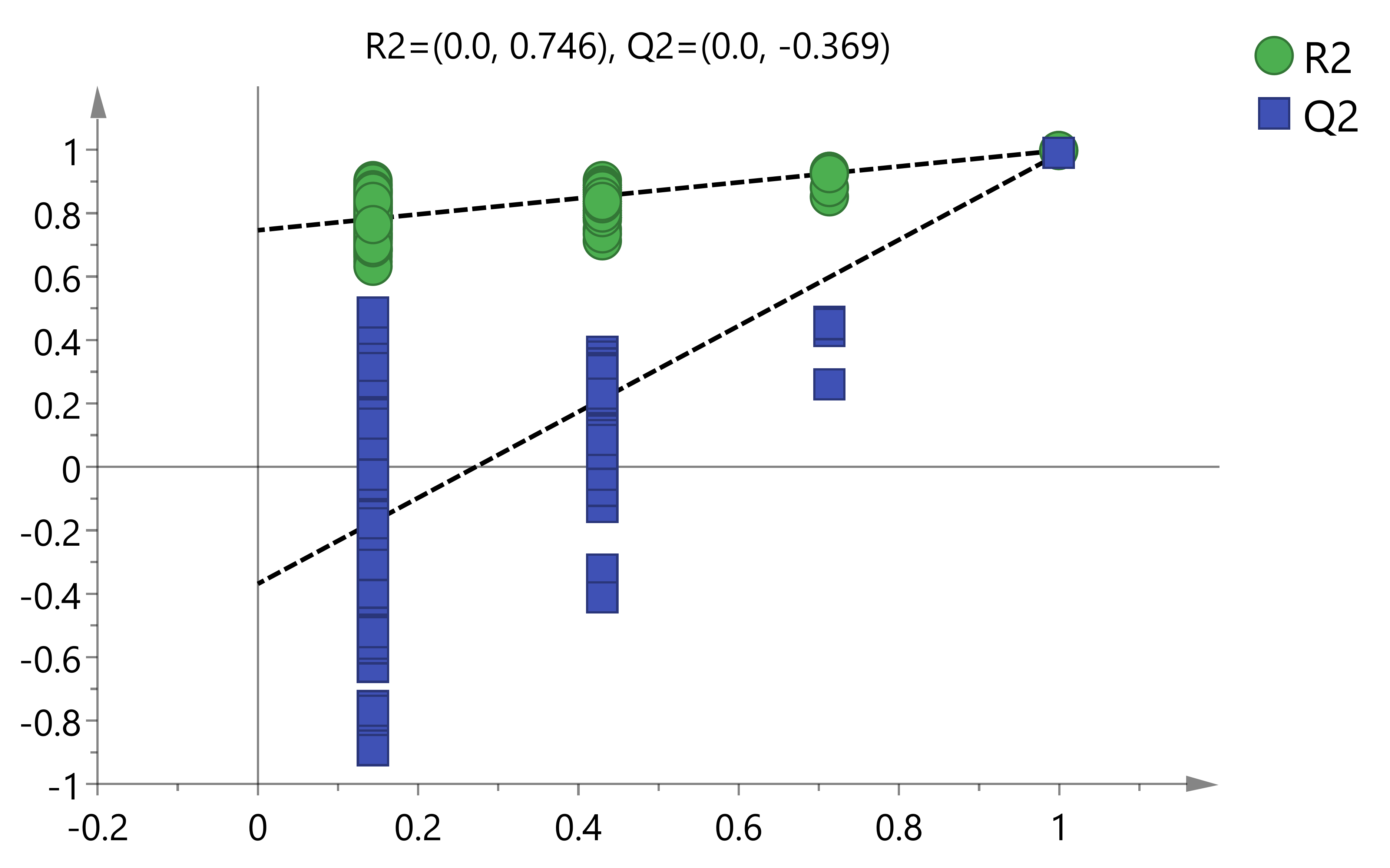

Supplement: Supplementary file 1 [file ijms-20-02330-s001.zip › supplementary material/2、Multivariate statistical analysis/Permutation(B0-18).tif]

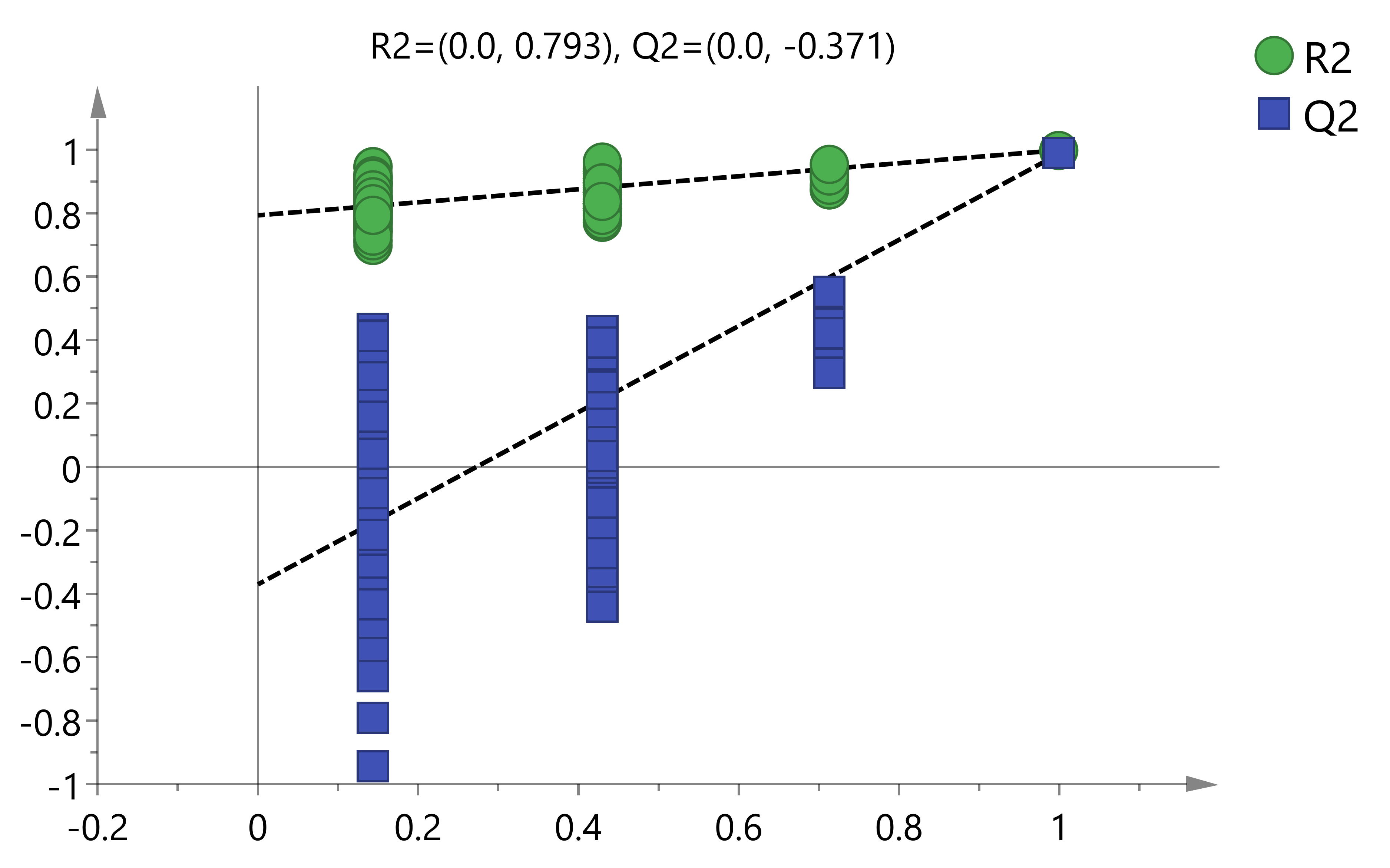

Supplement: Supplementary file 1 [file ijms-20-02330-s001.zip › supplementary material/2、Multivariate statistical analysis/Permutation(B0-24).tif]

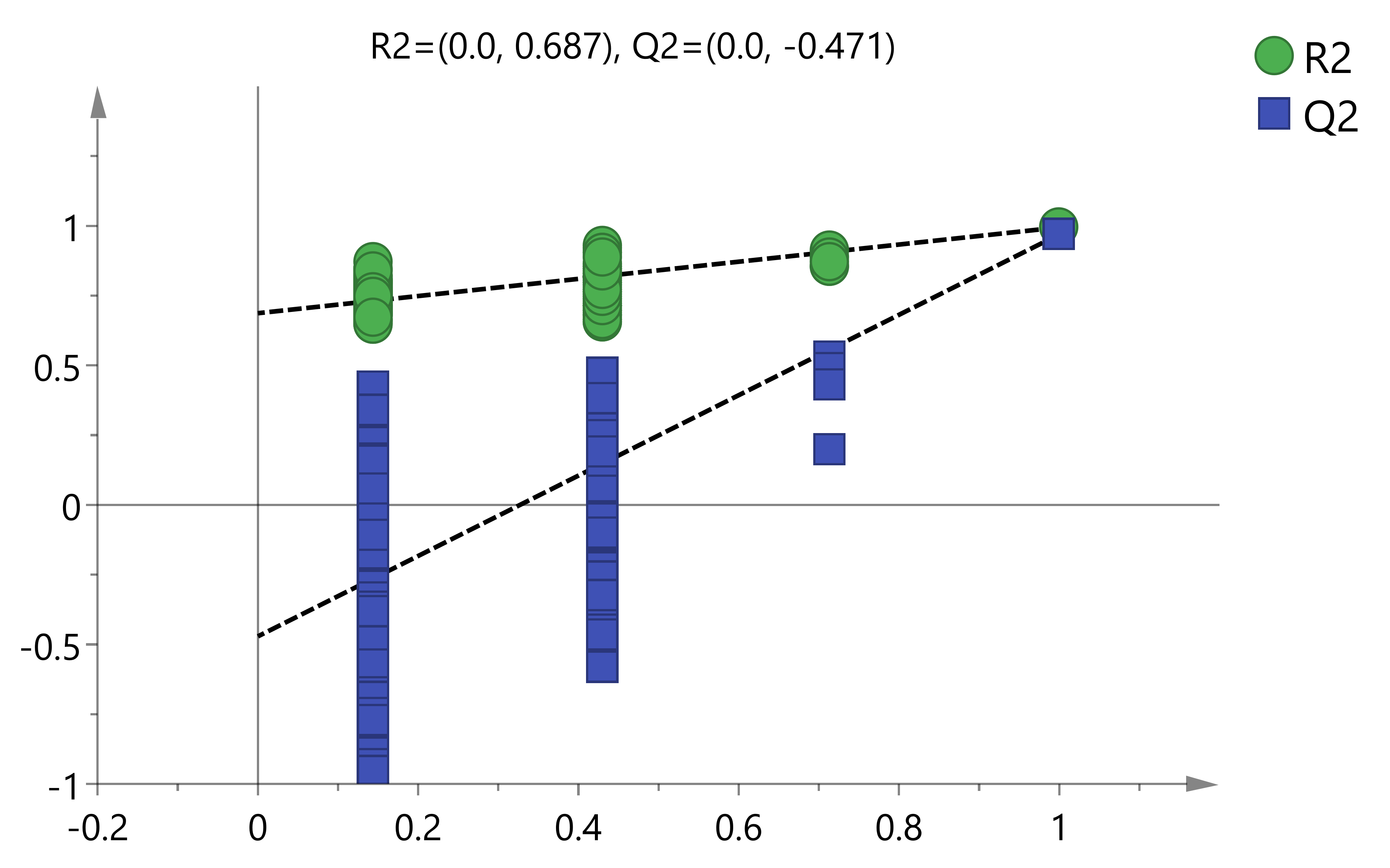

Supplement: Supplementary file 1 [file ijms-20-02330-s001.zip › supplementary material/2、Multivariate statistical analysis/Permutation(B0-4).tif]

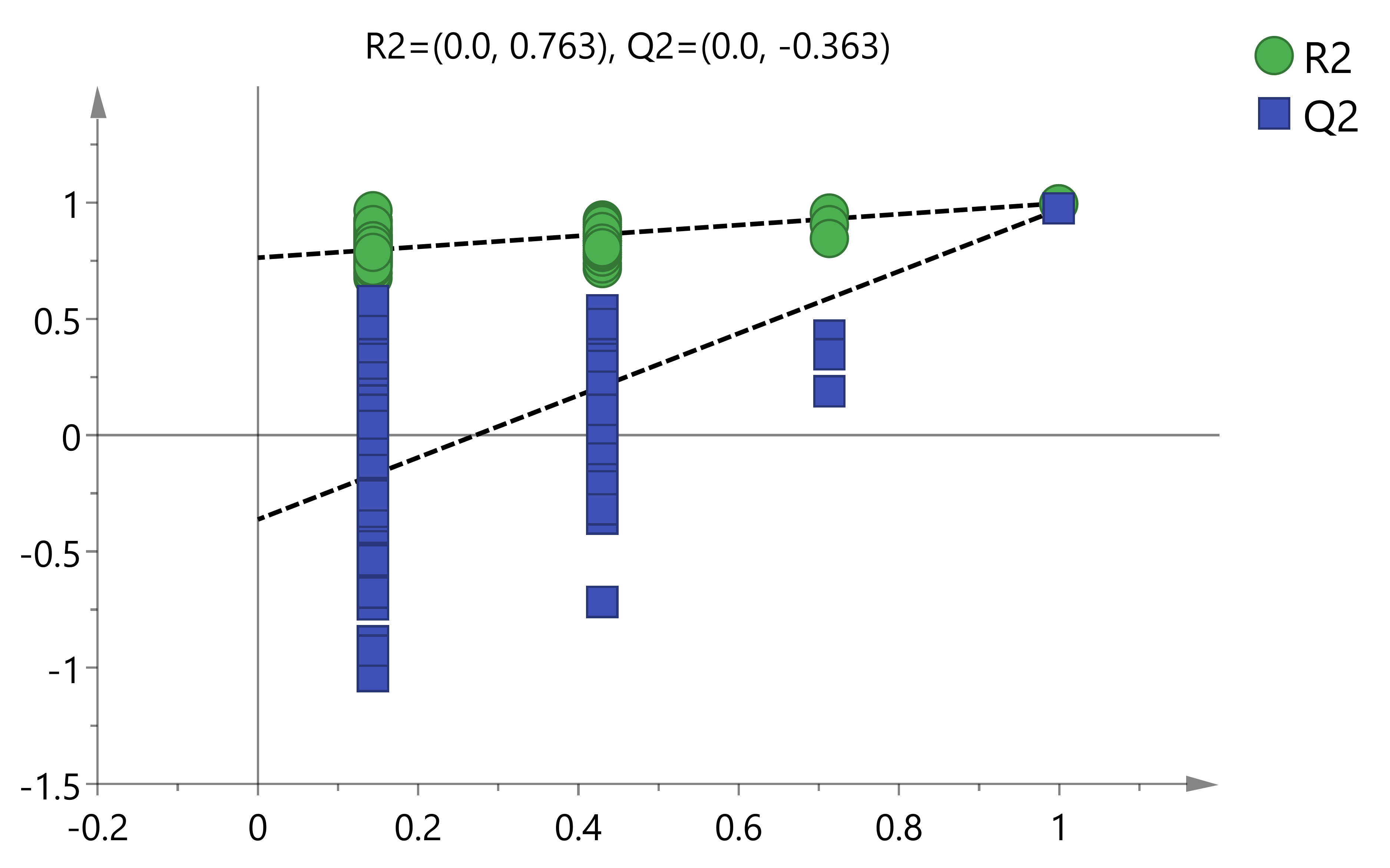

Supplement: Supplementary file 1 [file ijms-20-02330-s001.zip › supplementary material/2、Multivariate statistical analysis/Permutation(B0-8).tif]

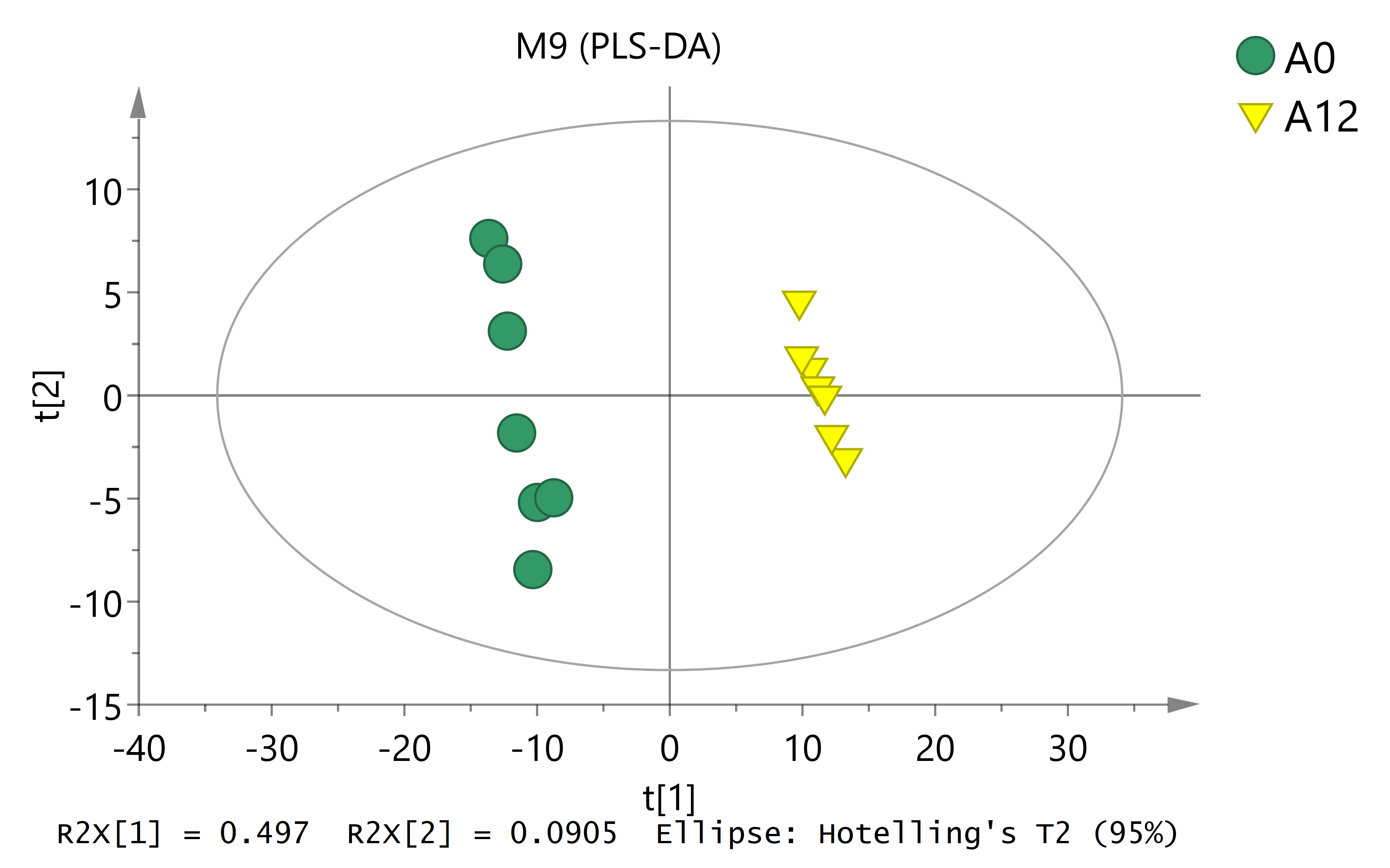

Supplement: Supplementary file 1 [file ijms-20-02330-s001.zip › supplementary material/2、Multivariate statistical analysis/pls(A0-12).tif]

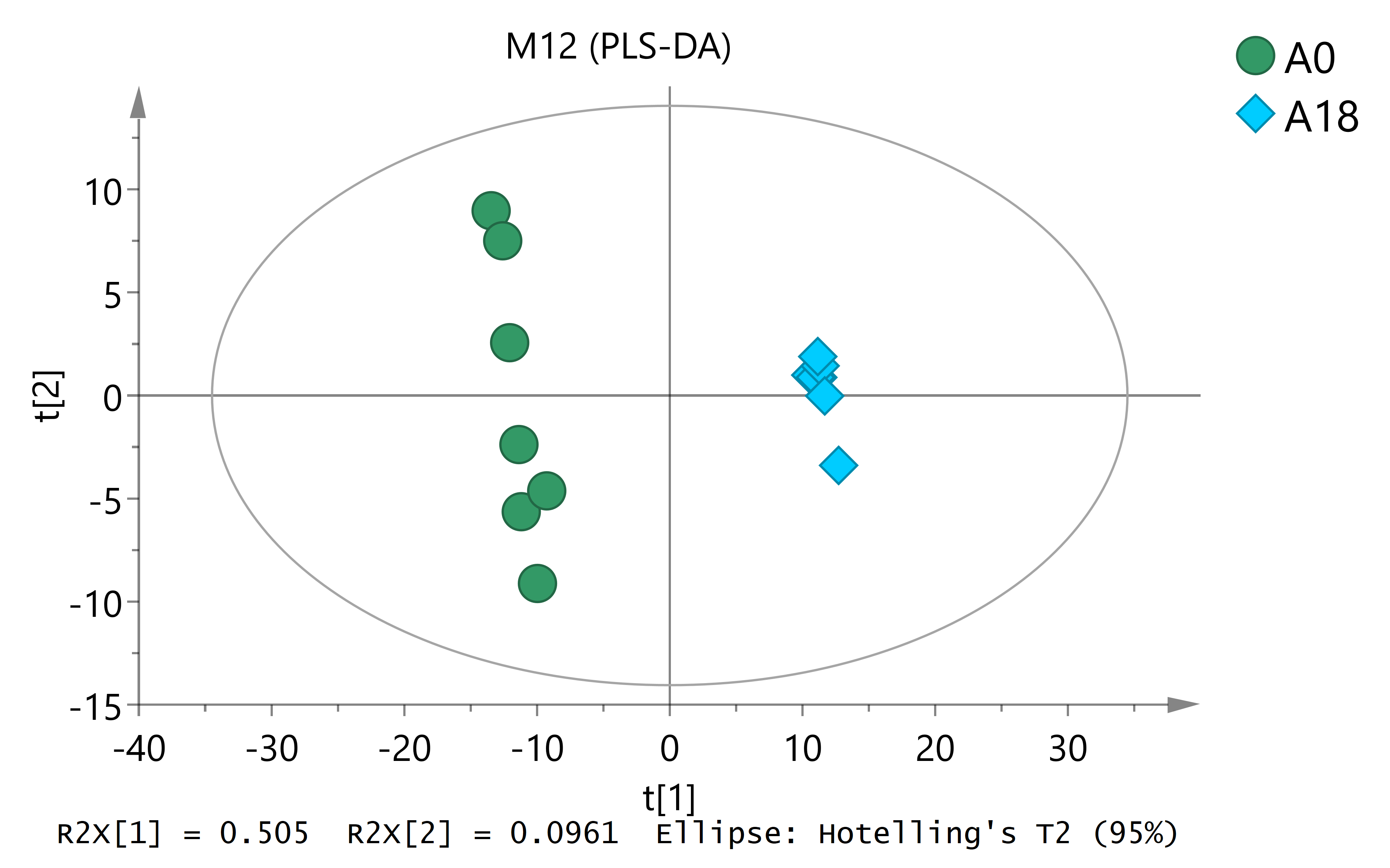

Supplement: Supplementary file 1 [file ijms-20-02330-s001.zip › supplementary material/2、Multivariate statistical analysis/pls(A0-18).tif]

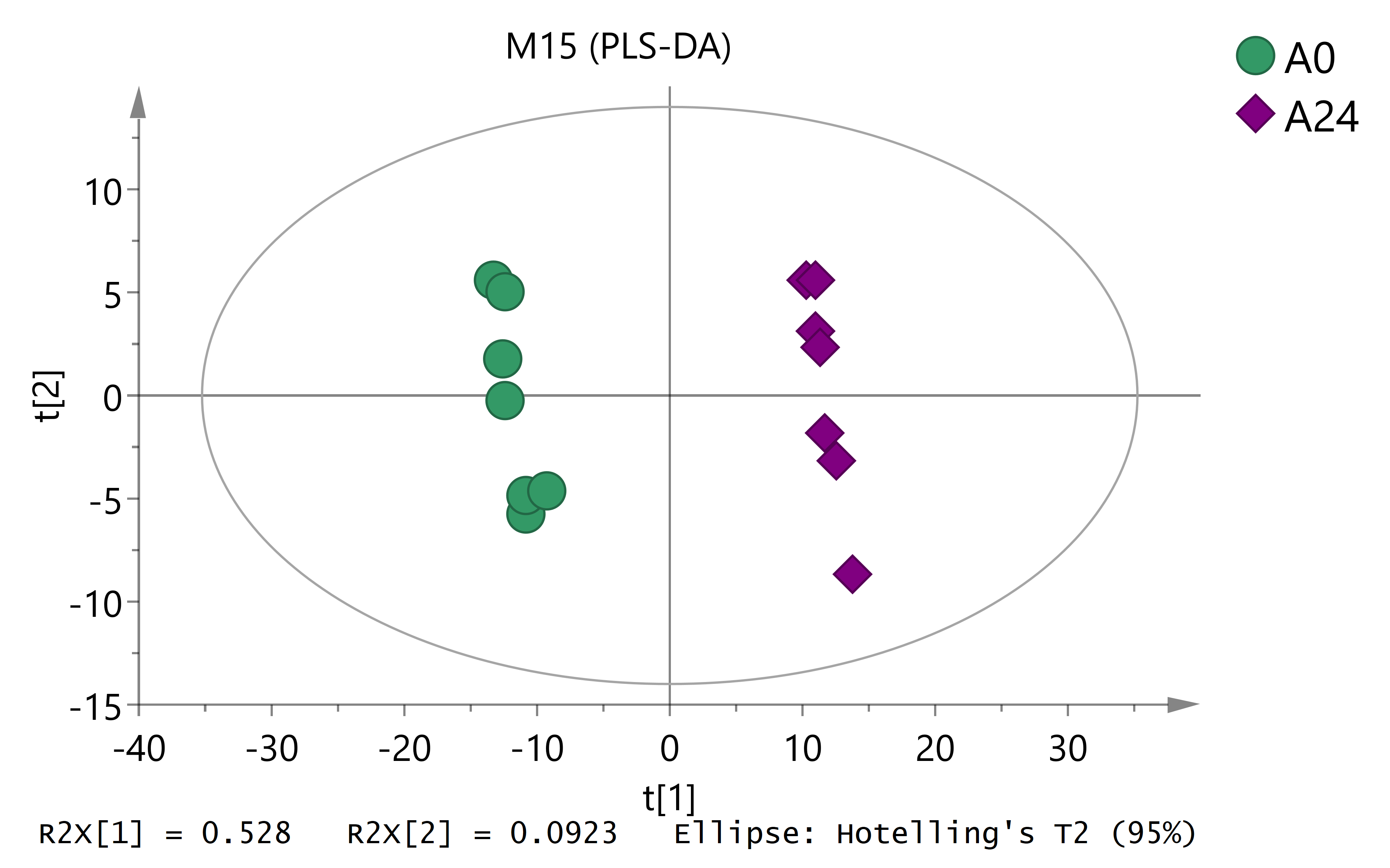

Supplement: Supplementary file 1 [file ijms-20-02330-s001.zip › supplementary material/2、Multivariate statistical analysis/pls(A0-24).tif]

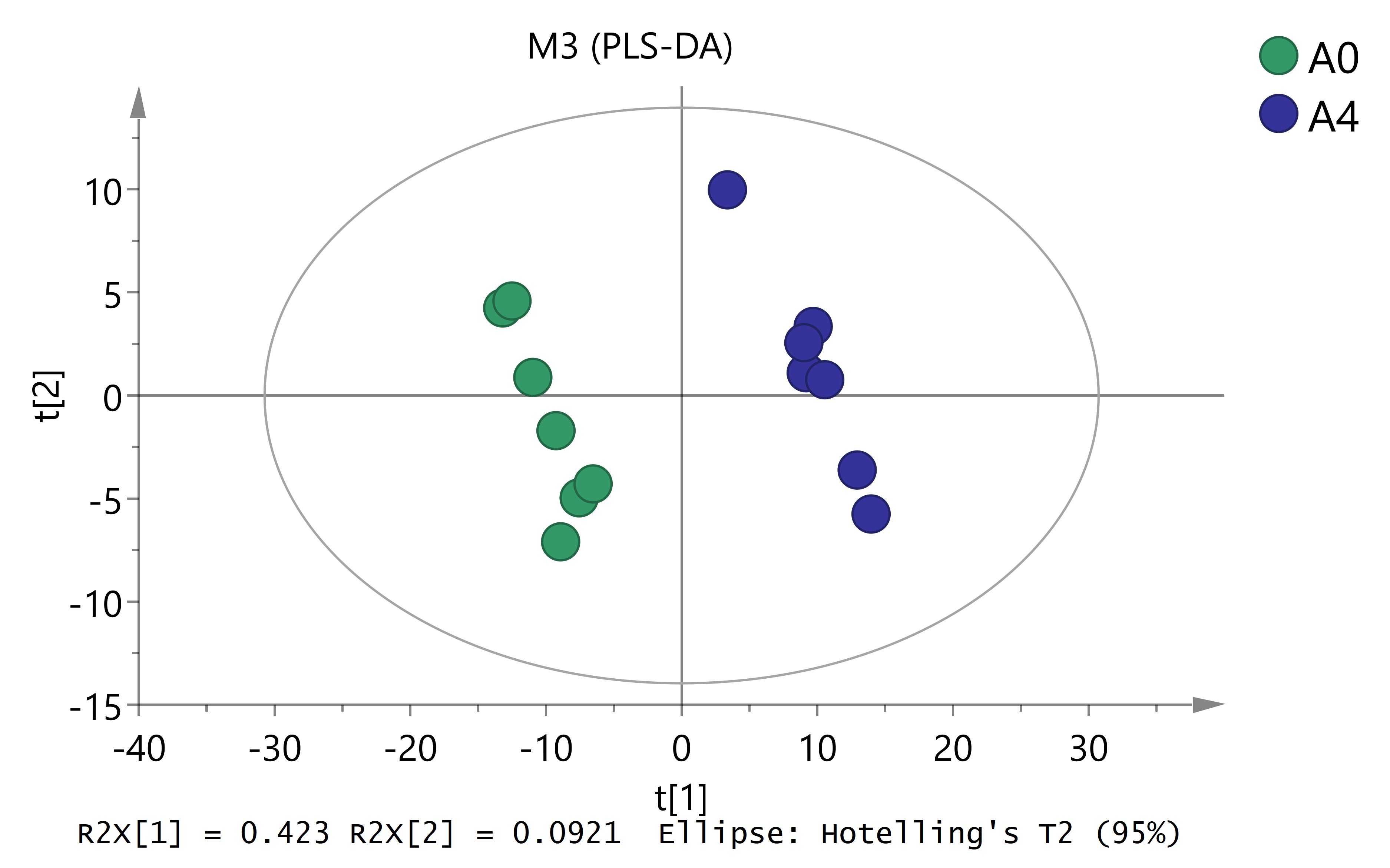

Supplement: Supplementary file 1 [file ijms-20-02330-s001.zip › supplementary material/2、Multivariate statistical analysis/pls(A0-4).tif]

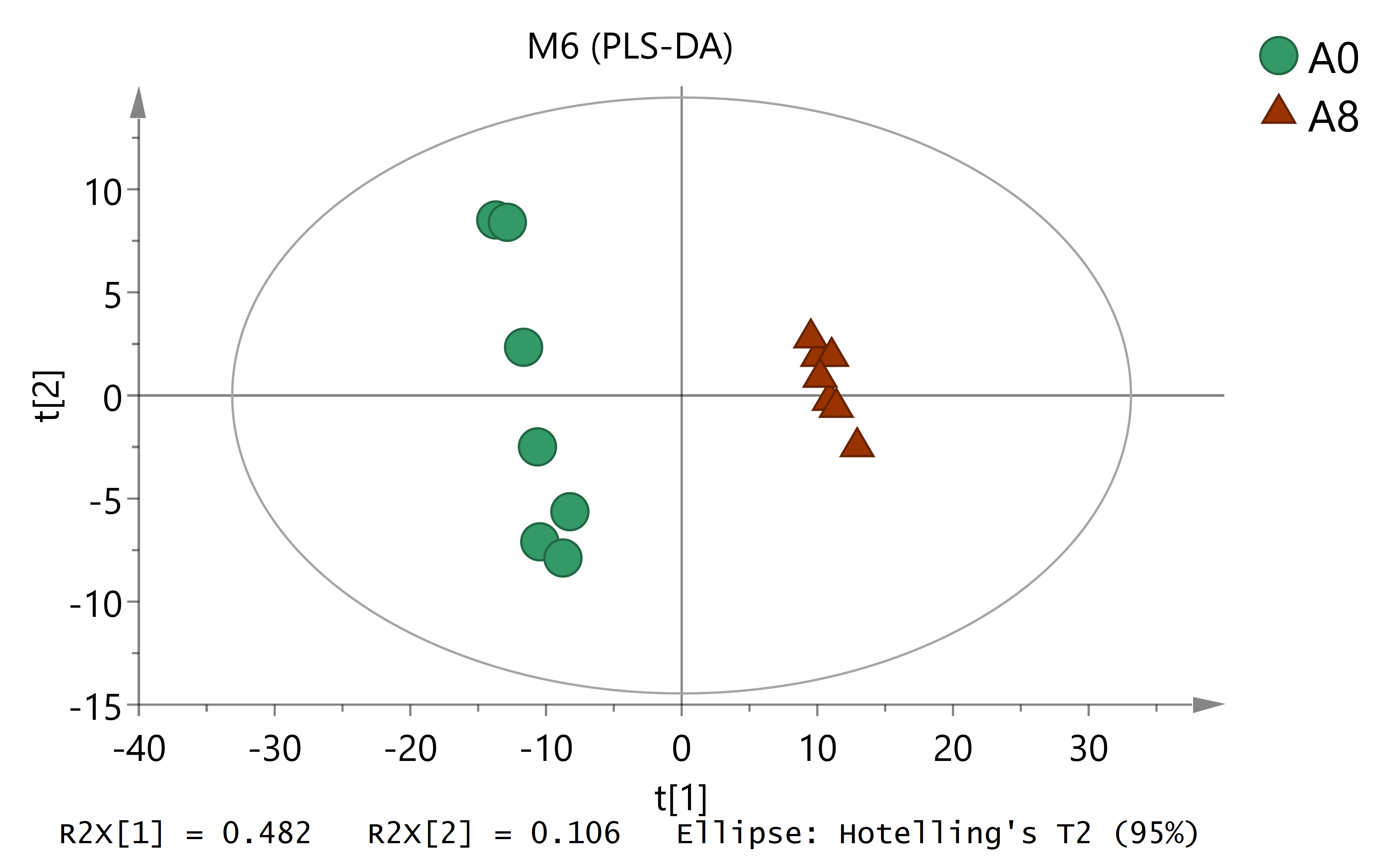

Supplement: Supplementary file 1 [file ijms-20-02330-s001.zip › supplementary material/2、Multivariate statistical analysis/pls(A0-8).tif]

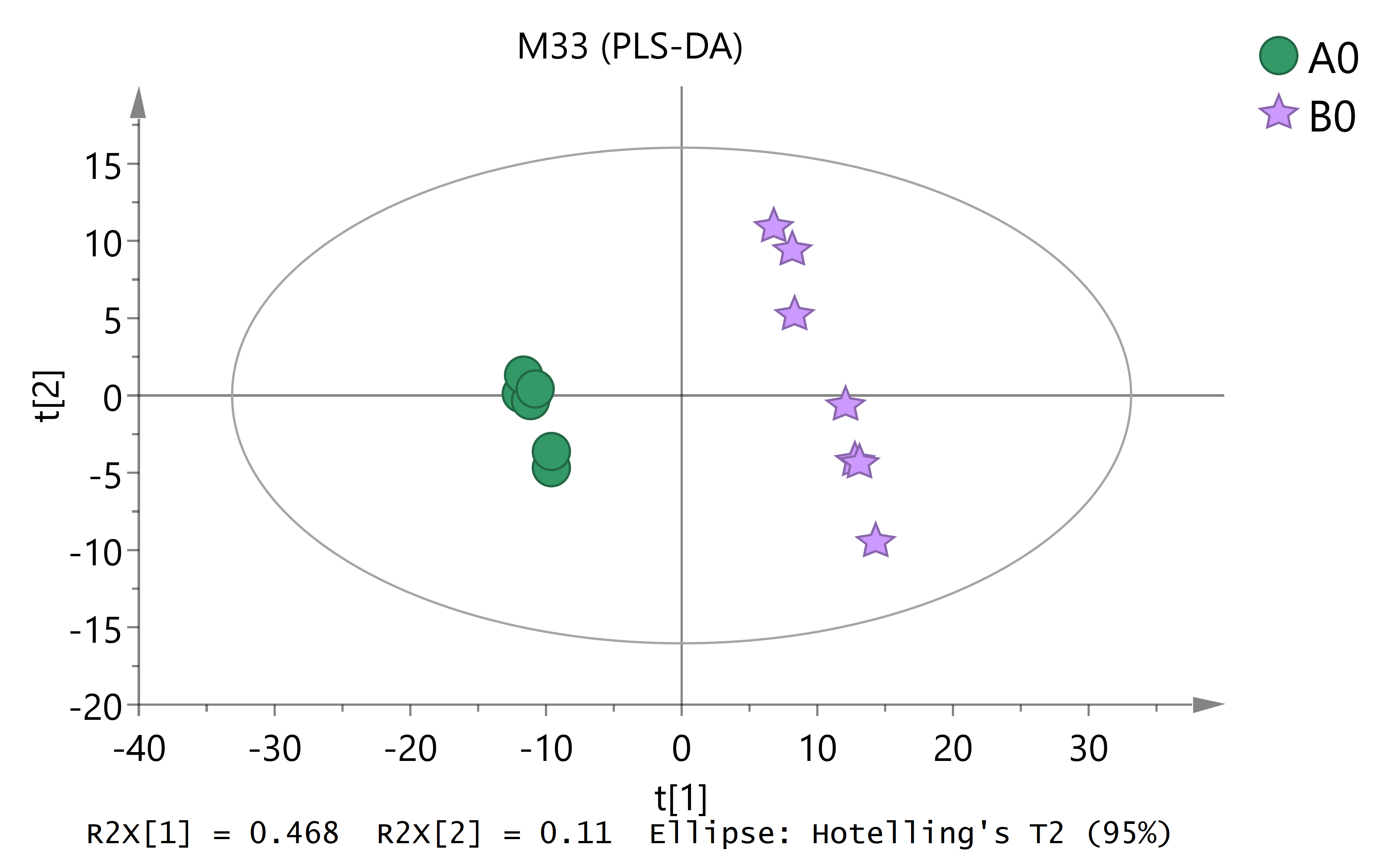

Supplement: Supplementary file 1 [file ijms-20-02330-s001.zip › supplementary material/2、Multivariate statistical analysis/pls(A0-B0).tif]

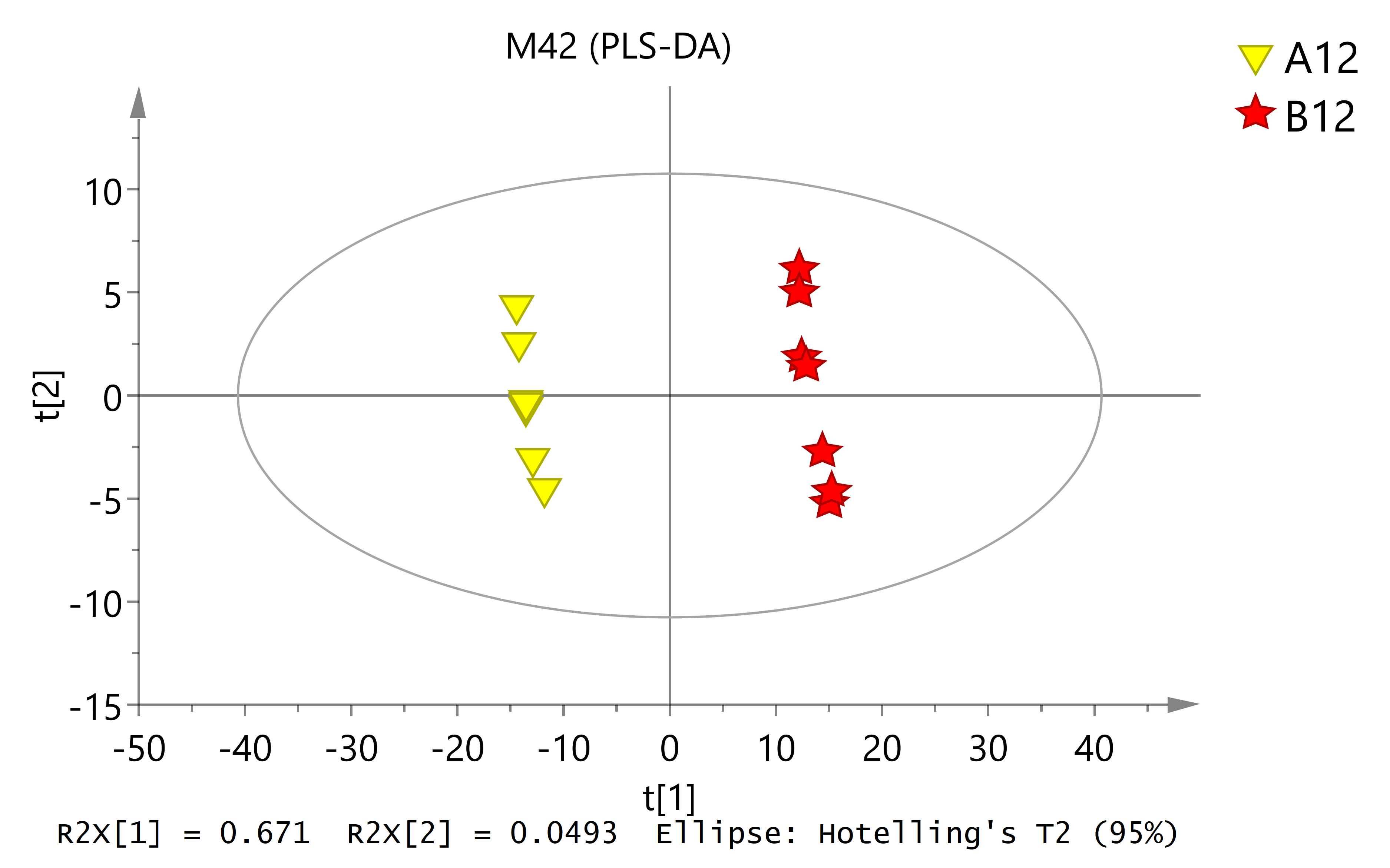

Supplement: Supplementary file 1 [file ijms-20-02330-s001.zip › supplementary material/2、Multivariate statistical analysis/pls(A12-B12).tif]

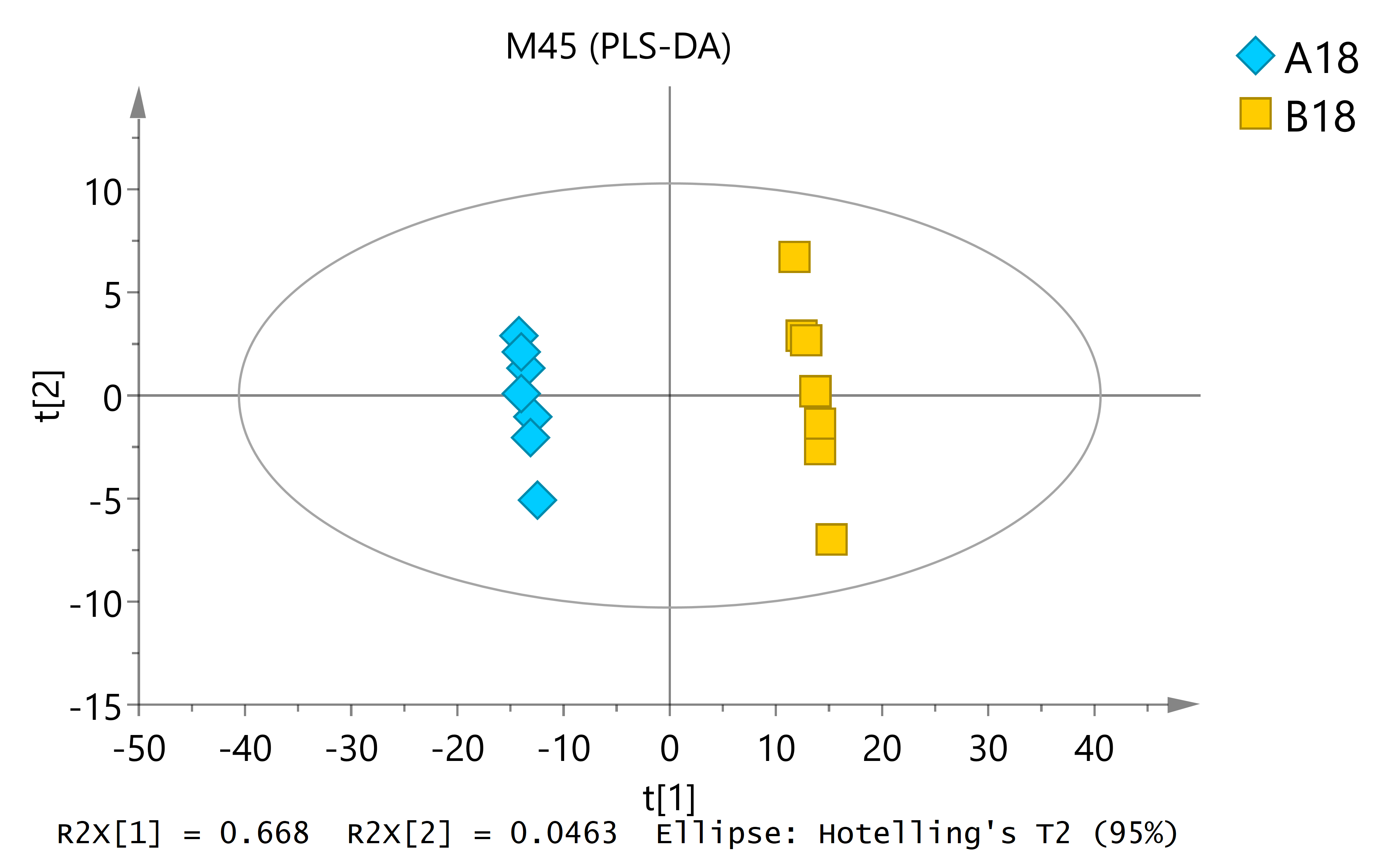

Supplement: Supplementary file 1 [file ijms-20-02330-s001.zip › supplementary material/2、Multivariate statistical analysis/pls(A18-B18).tif]

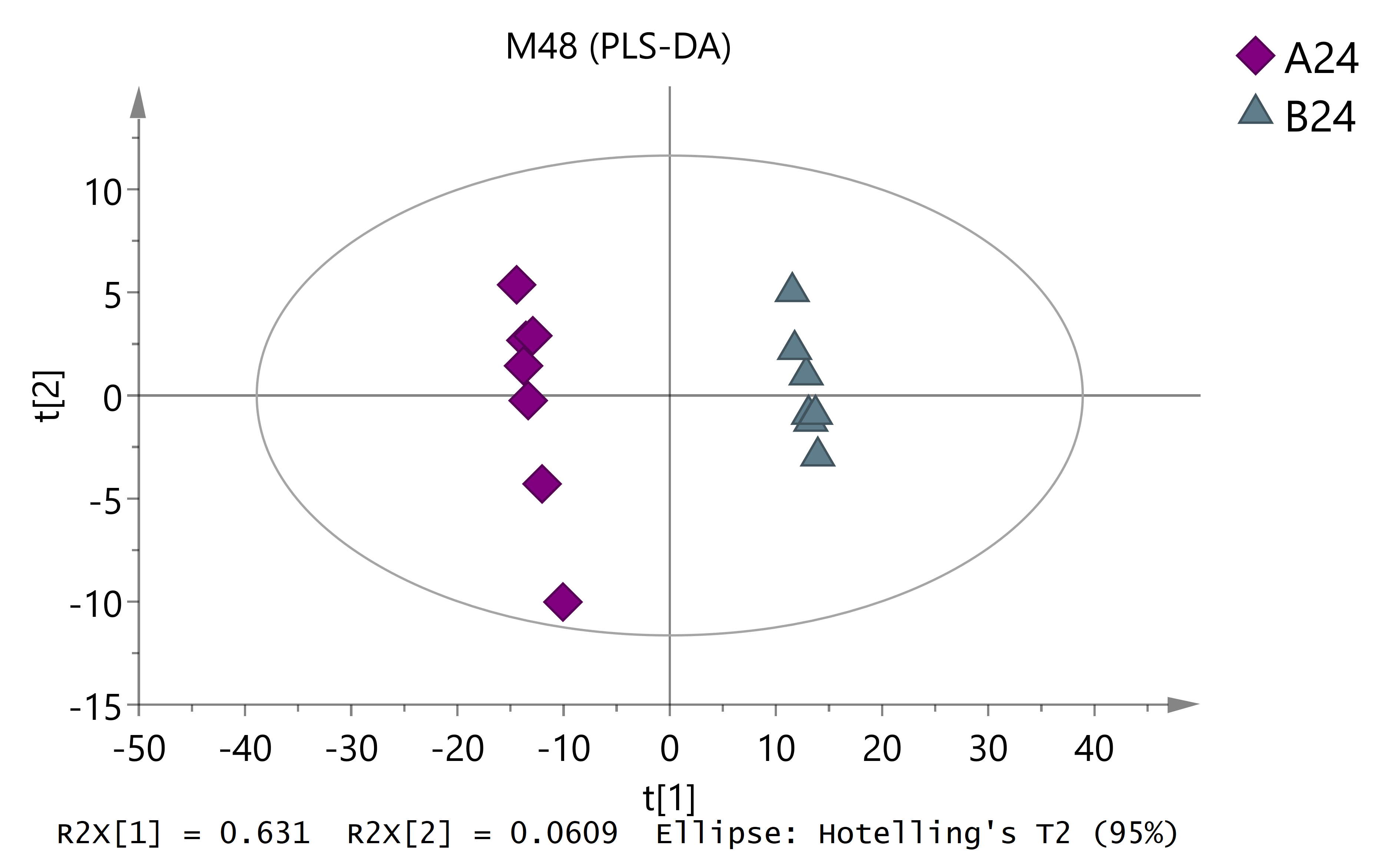

Supplement: Supplementary file 1 [file ijms-20-02330-s001.zip › supplementary material/2、Multivariate statistical analysis/pls(A24-B24).tif]

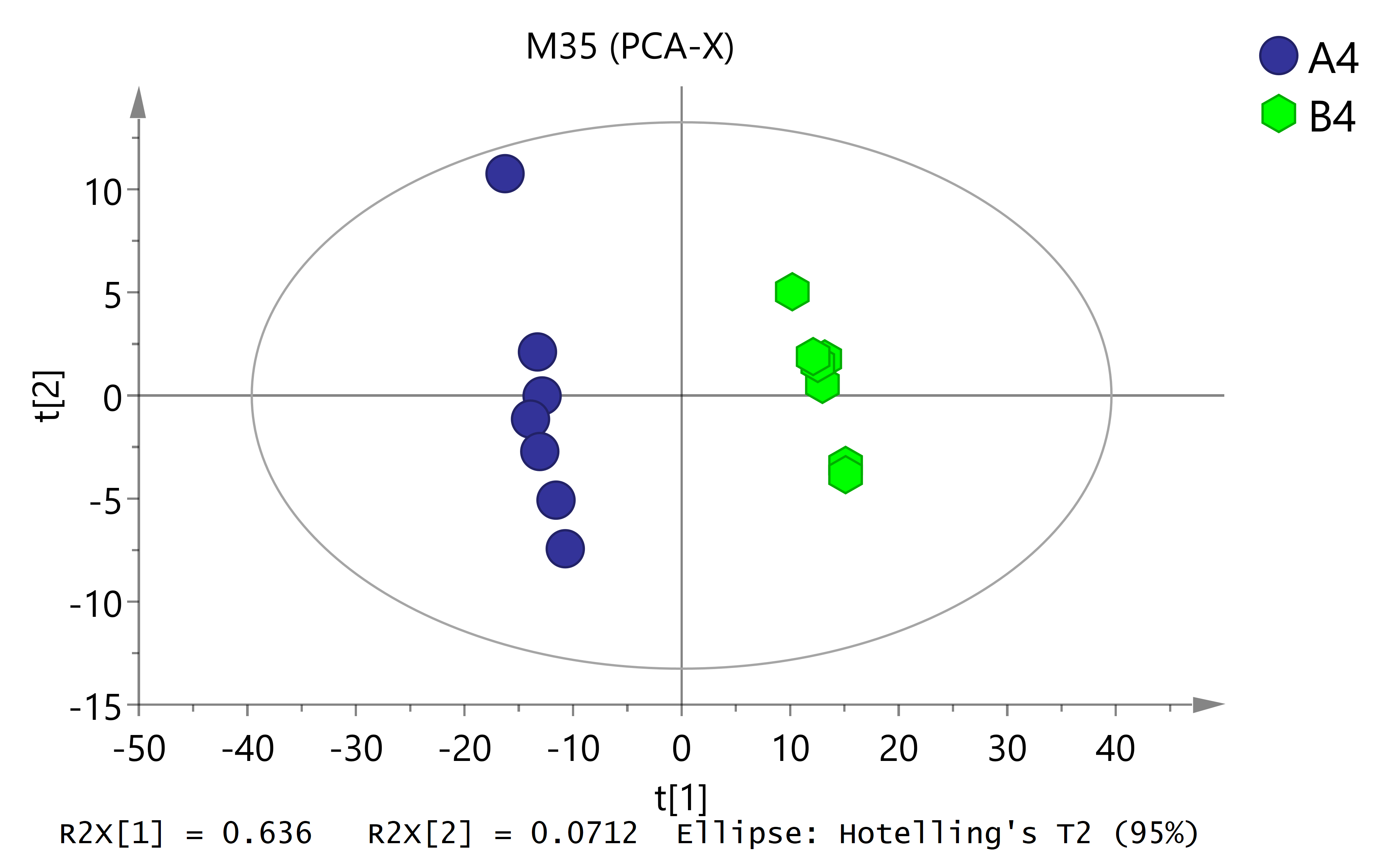

Supplement: Supplementary file 1 [file ijms-20-02330-s001.zip › supplementary material/2、Multivariate statistical analysis/pls(A4-B4).tif]

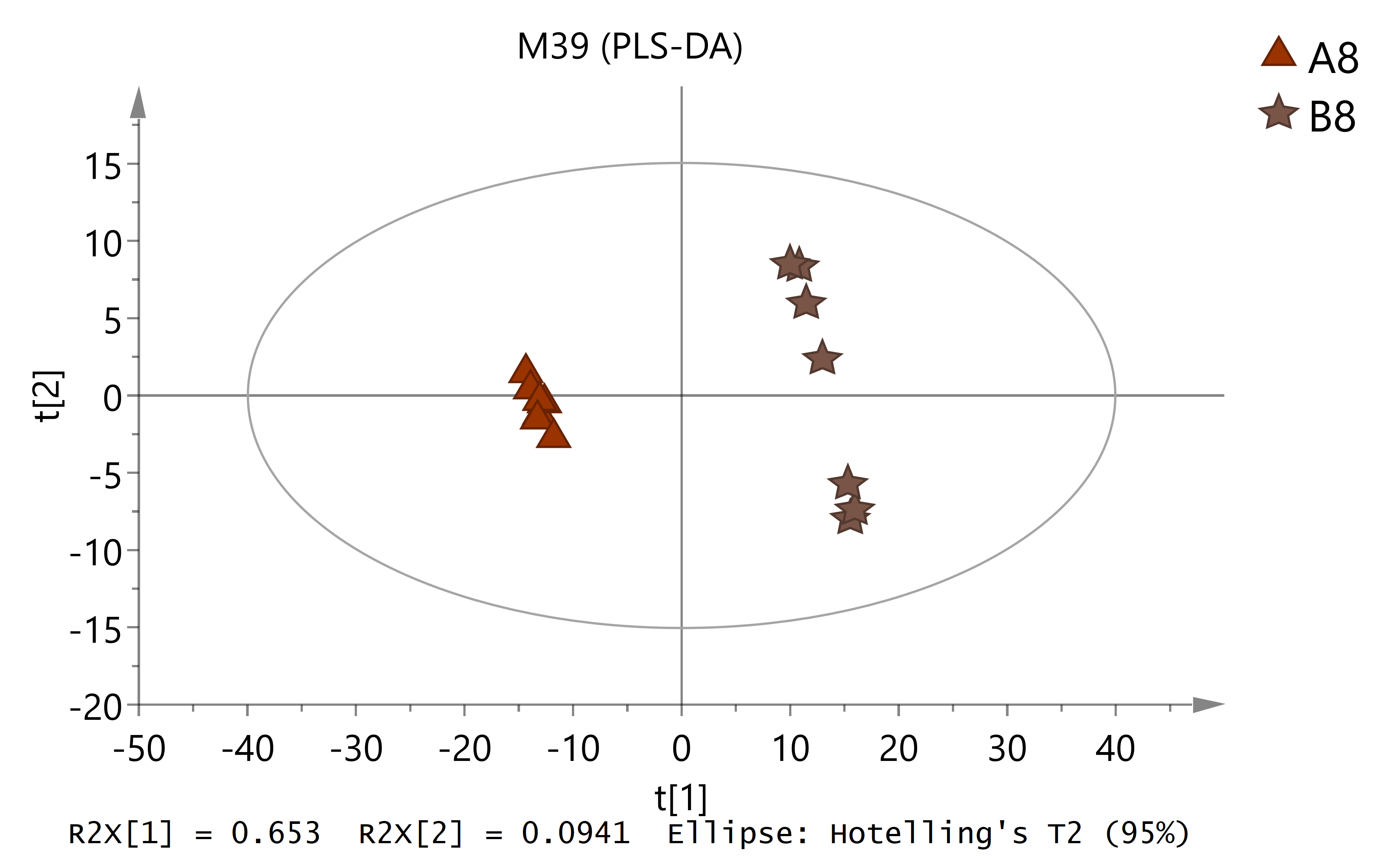

Supplement: Supplementary file 1 [file ijms-20-02330-s001.zip › supplementary material/2、Multivariate statistical analysis/pls(A8-B8).tif]

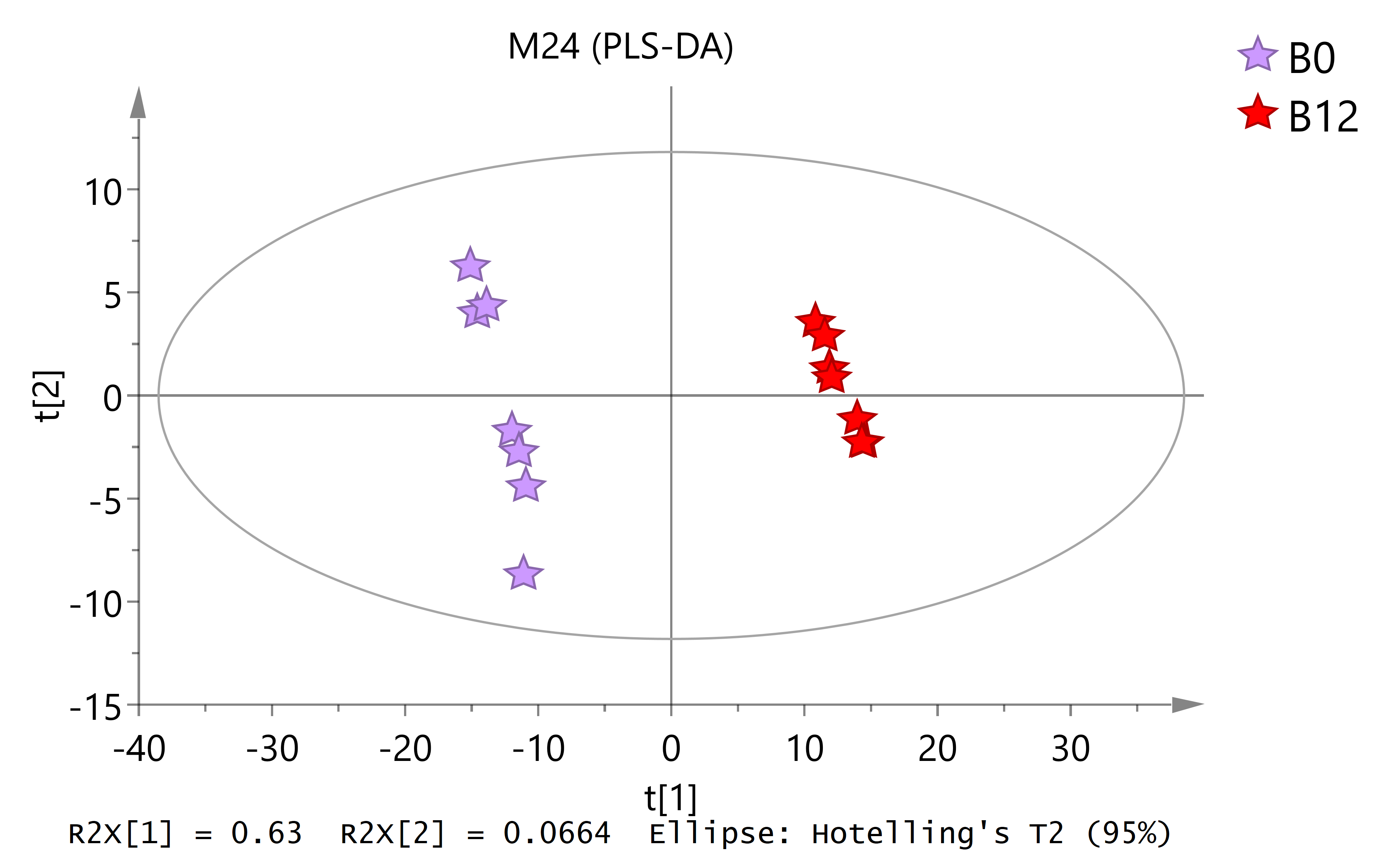

Supplement: Supplementary file 1 [file ijms-20-02330-s001.zip › supplementary material/2、Multivariate statistical analysis/pls(B0-12).tif]

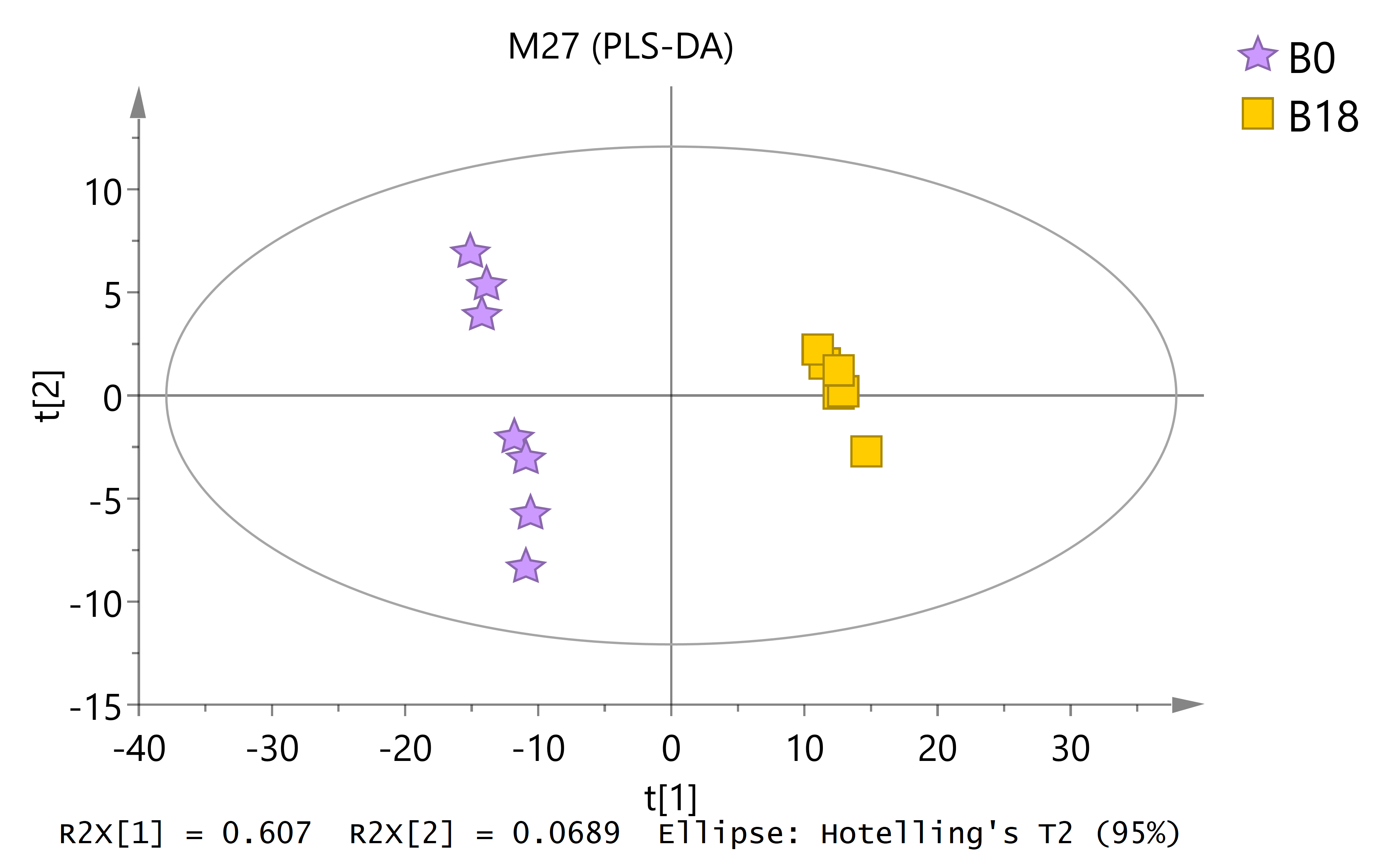

Supplement: Supplementary file 1 [file ijms-20-02330-s001.zip › supplementary material/2、Multivariate statistical analysis/pls(B0-18).tif]

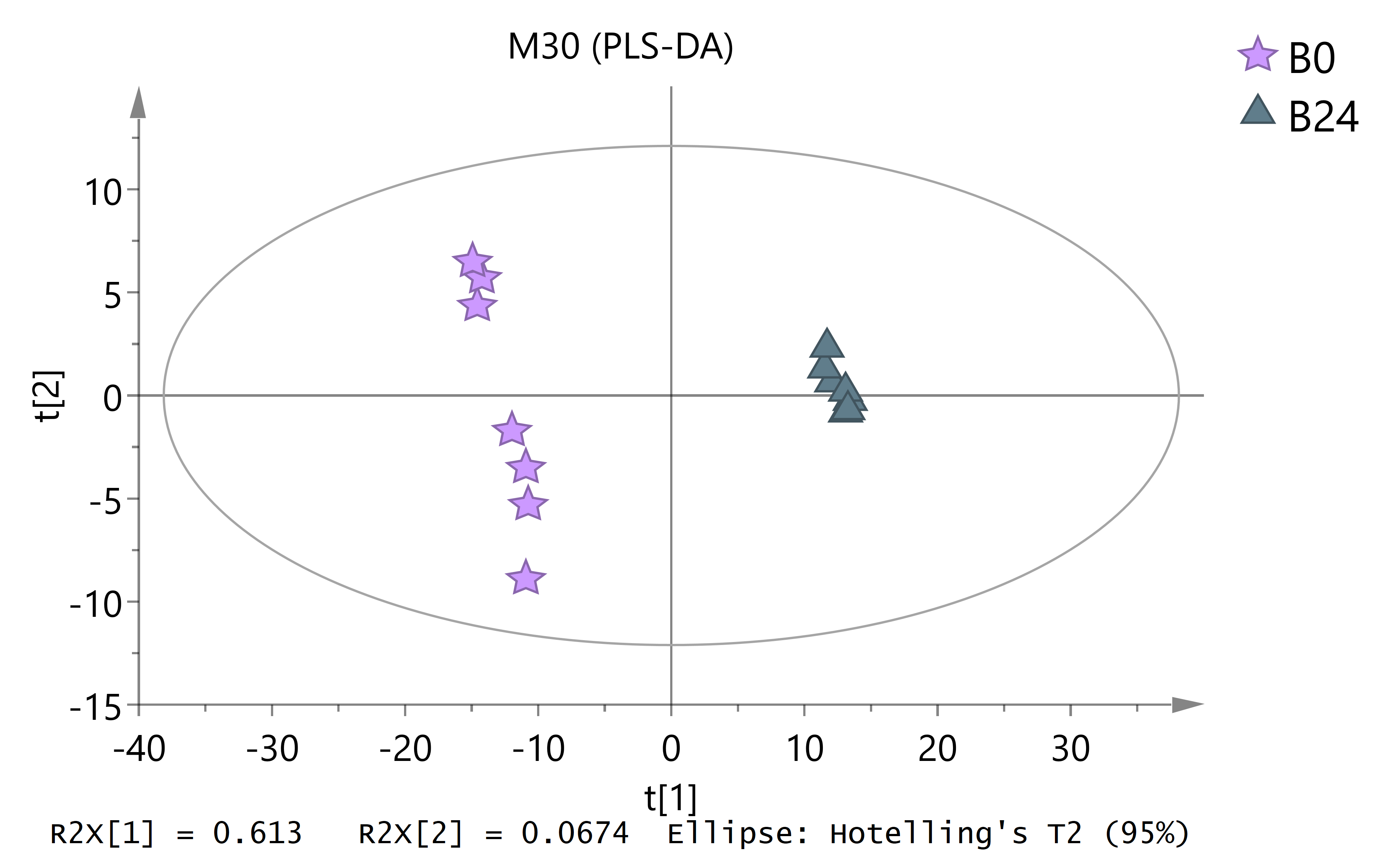

Supplement: Supplementary file 1 [file ijms-20-02330-s001.zip › supplementary material/2、Multivariate statistical analysis/pls(B0-24).tif]

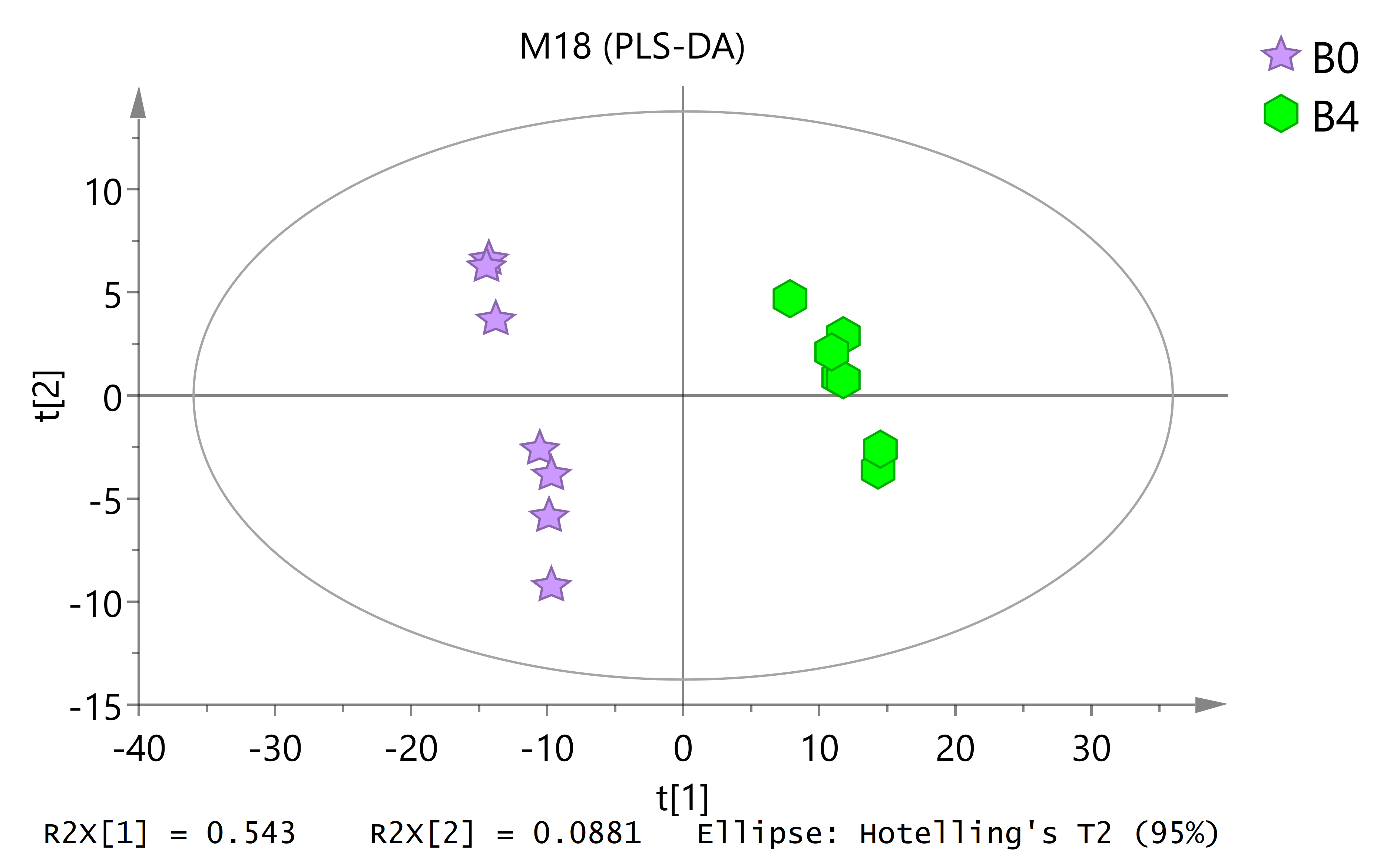

Supplement: Supplementary file 1 [file ijms-20-02330-s001.zip › supplementary material/2、Multivariate statistical analysis/pls(B0-4).tif]

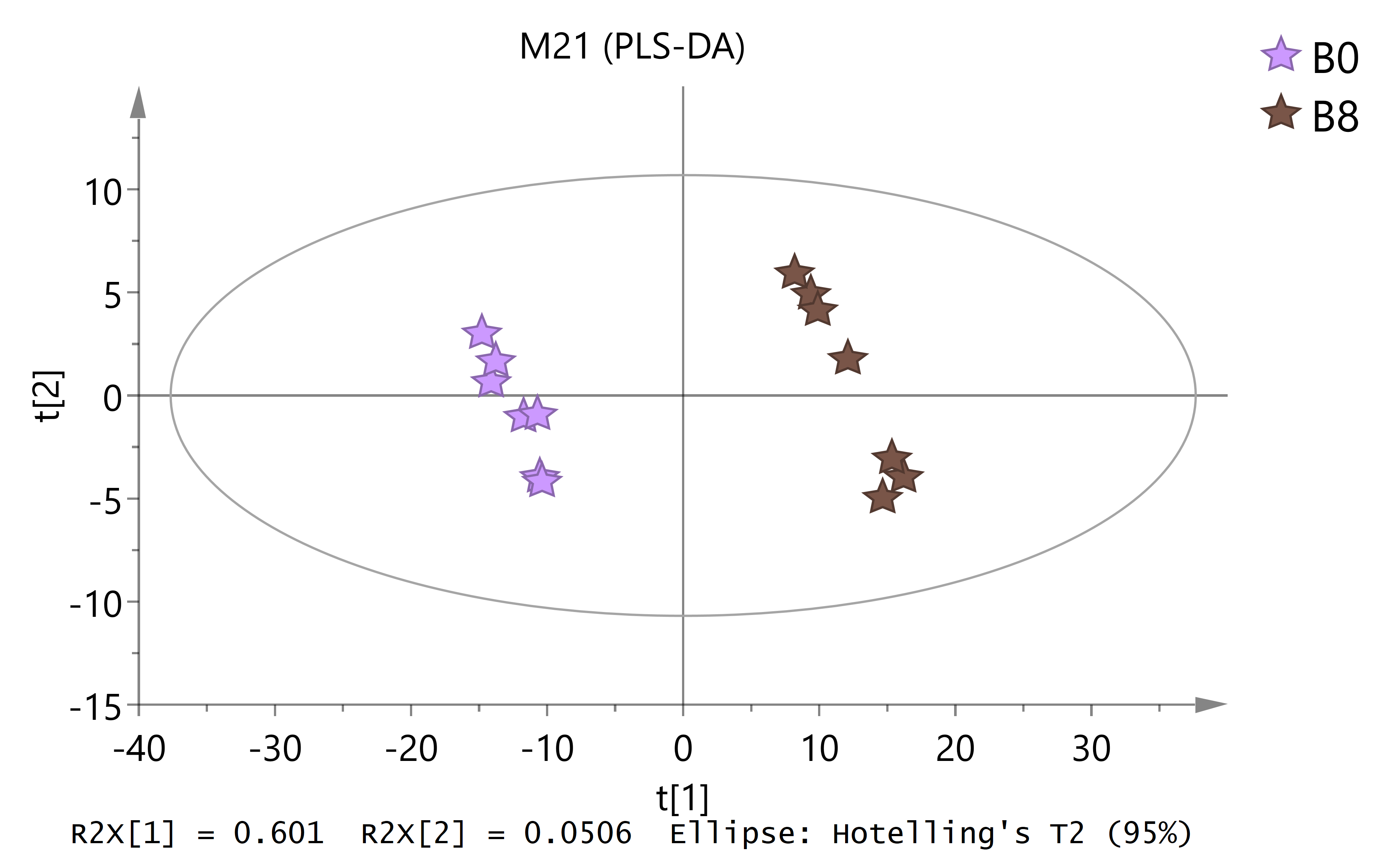

Supplement: Supplementary file 1 [file ijms-20-02330-s001.zip › supplementary material/2、Multivariate statistical analysis/pls(B0-8).tif]

Volcanoplot

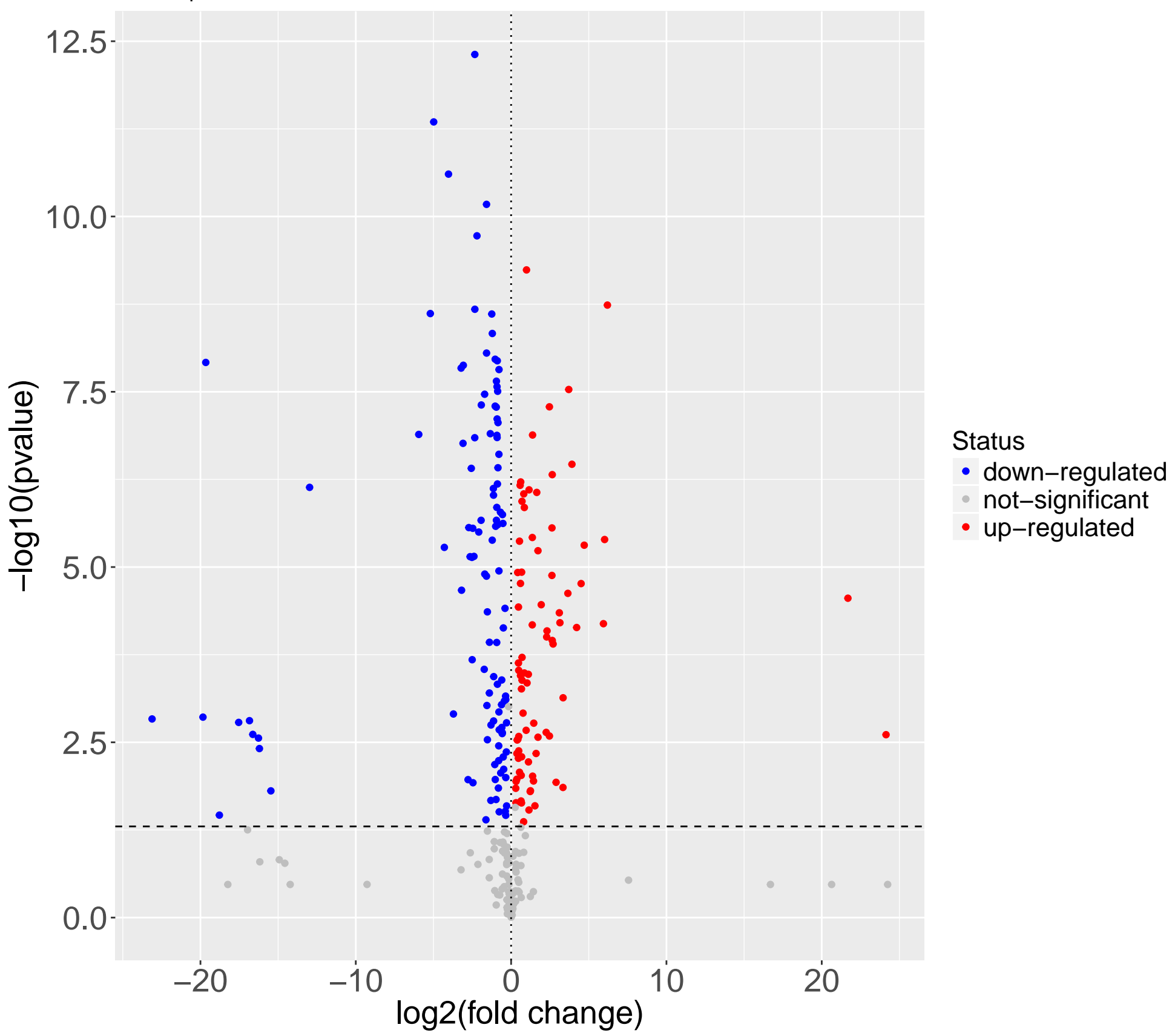

Supplement: Supplementary file 1 [file ijms-20-02330-s001.zip › supplementary material/3、The volcano figure/A0-A12.pdf]

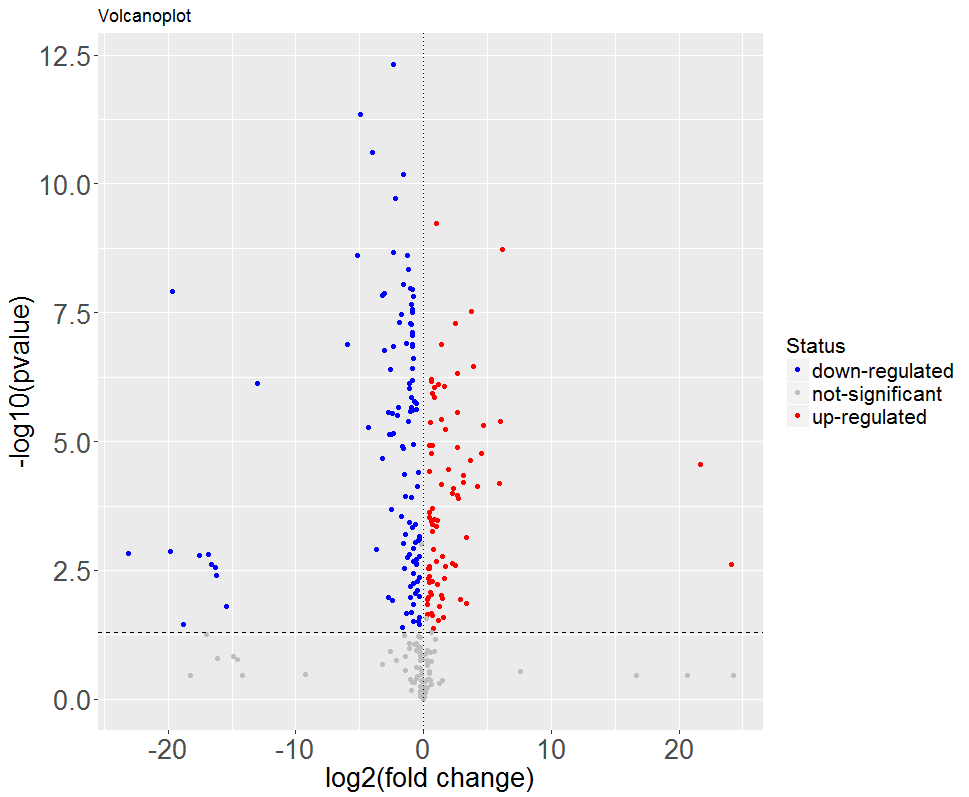

Supplement: Supplementary file 1 [file ijms-20-02330-s001.zip › supplementary material/3、The volcano figure/A0-A12.png]

Volcanoplot

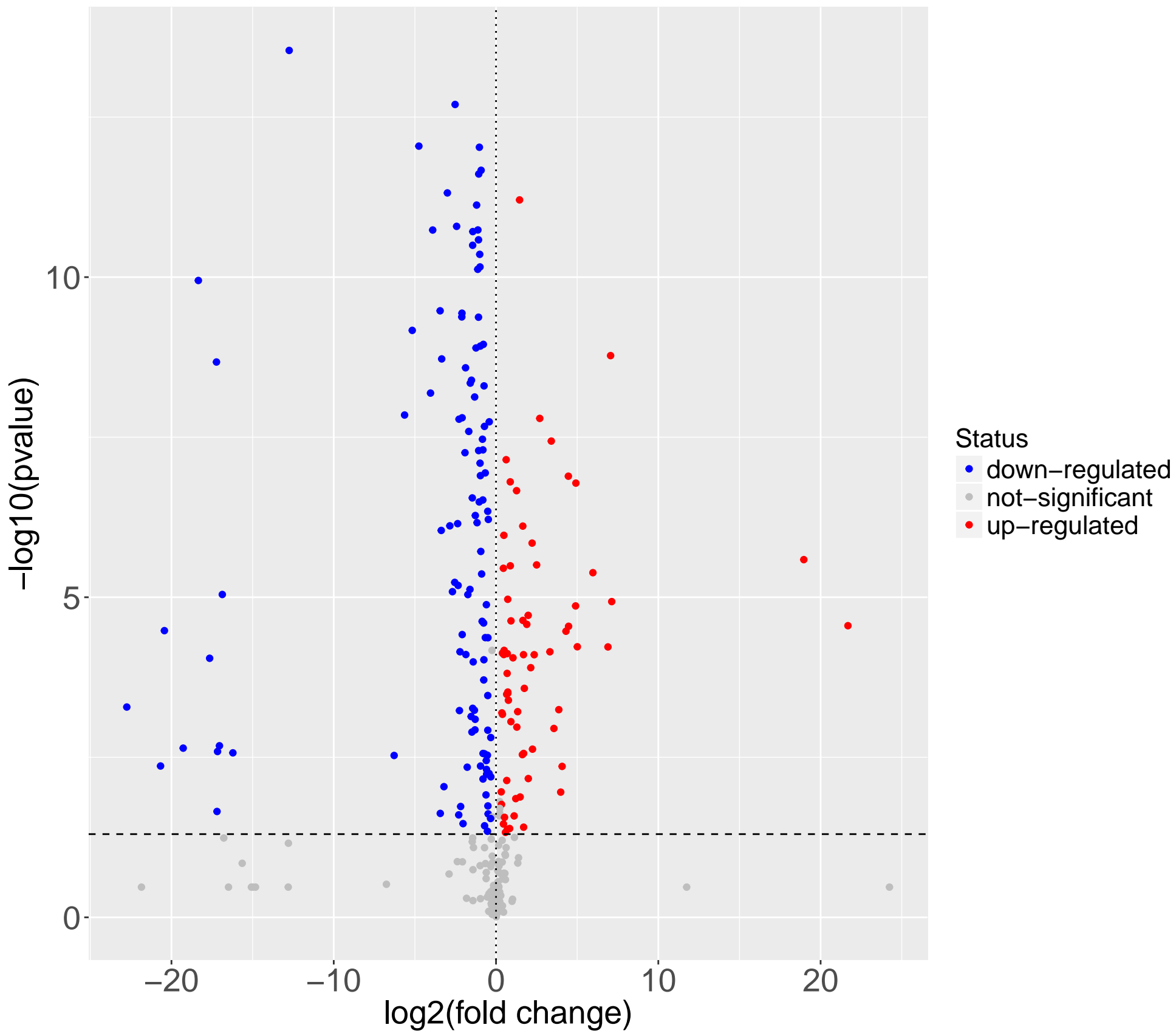

Supplement: Supplementary file 1 [file ijms-20-02330-s001.zip › supplementary material/3、The volcano figure/A0-A18.pdf]

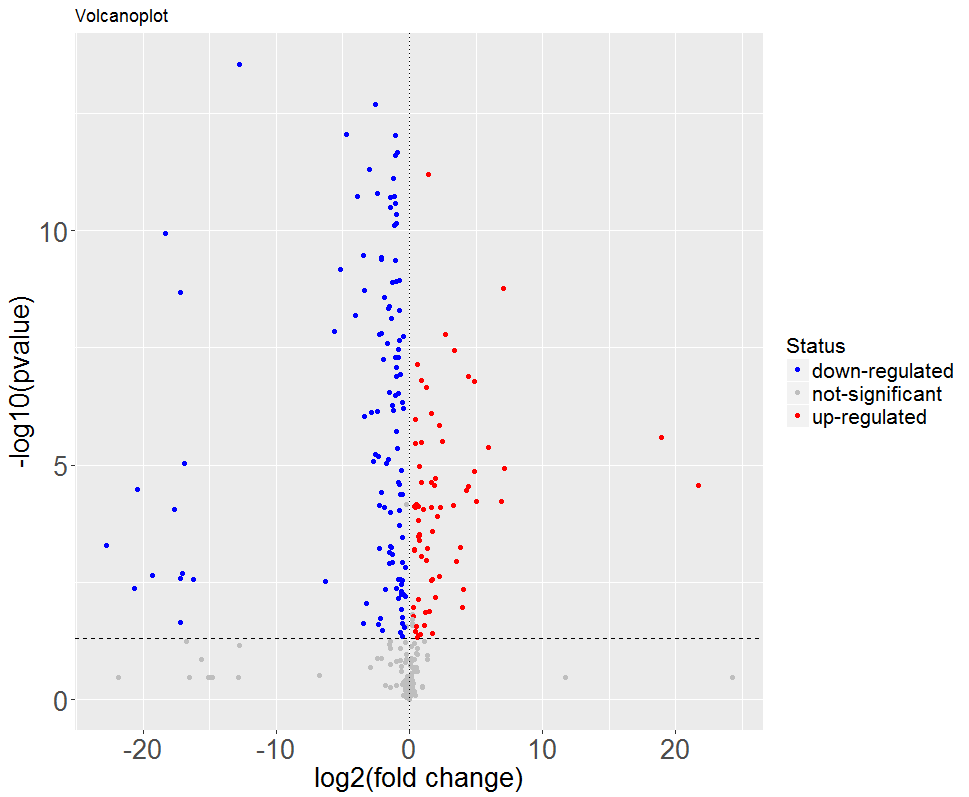

Supplement: Supplementary file 1 [file ijms-20-02330-s001.zip › supplementary material/3、The volcano figure/A0-A18.png]

Volcanoplot

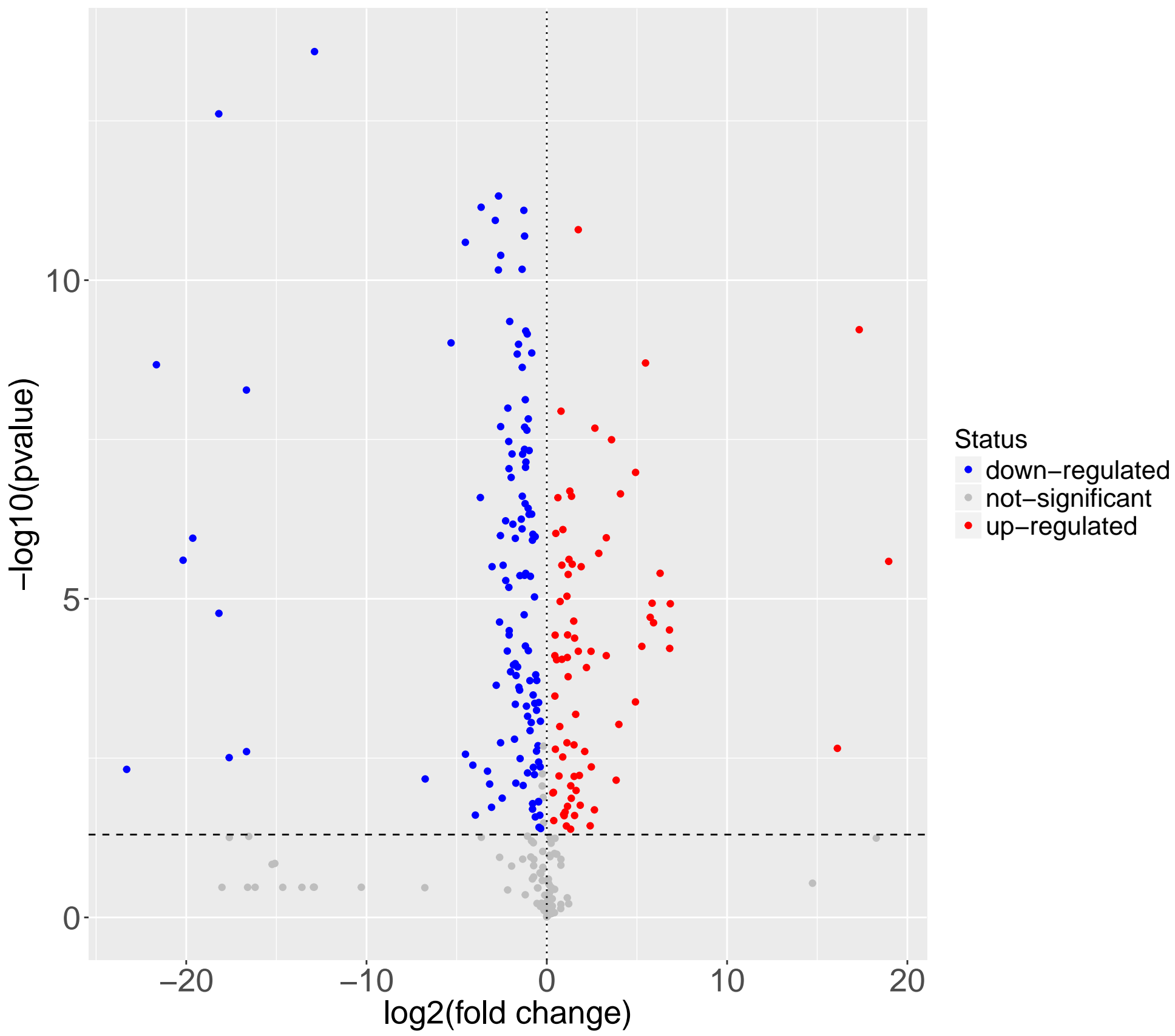

Supplement: Supplementary file 1 [file ijms-20-02330-s001.zip › supplementary material/3、The volcano figure/A0-A24.pdf]

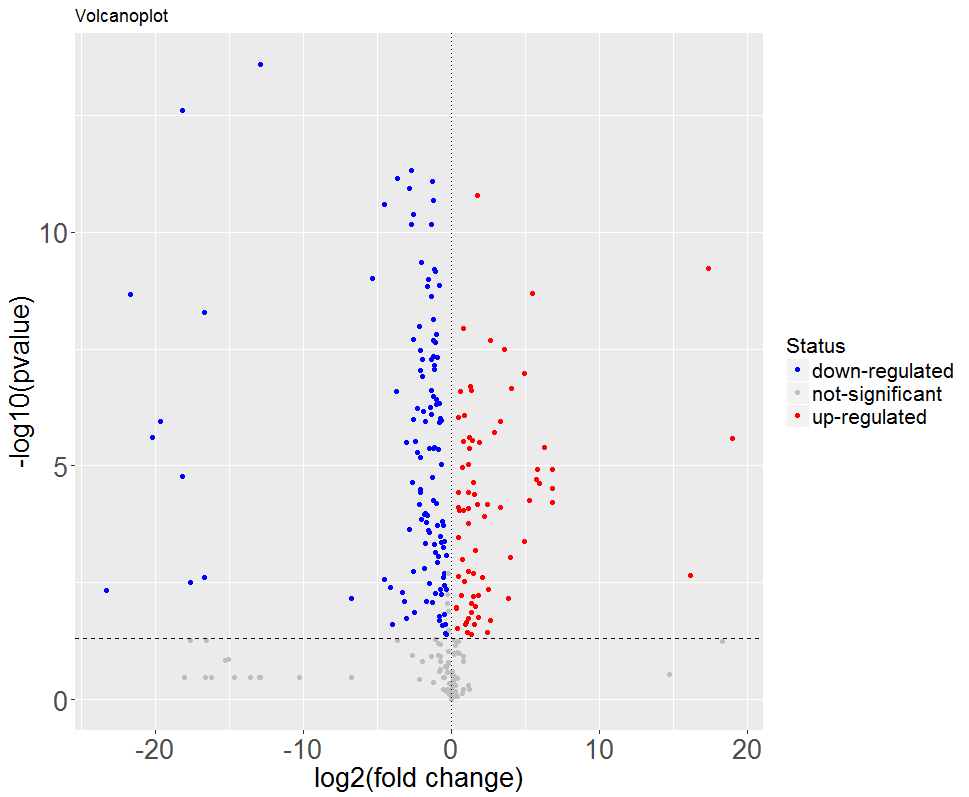

Supplement: Supplementary file 1 [file ijms-20-02330-s001.zip › supplementary material/3、The volcano figure/A0-A24.png]

Volcanoplot

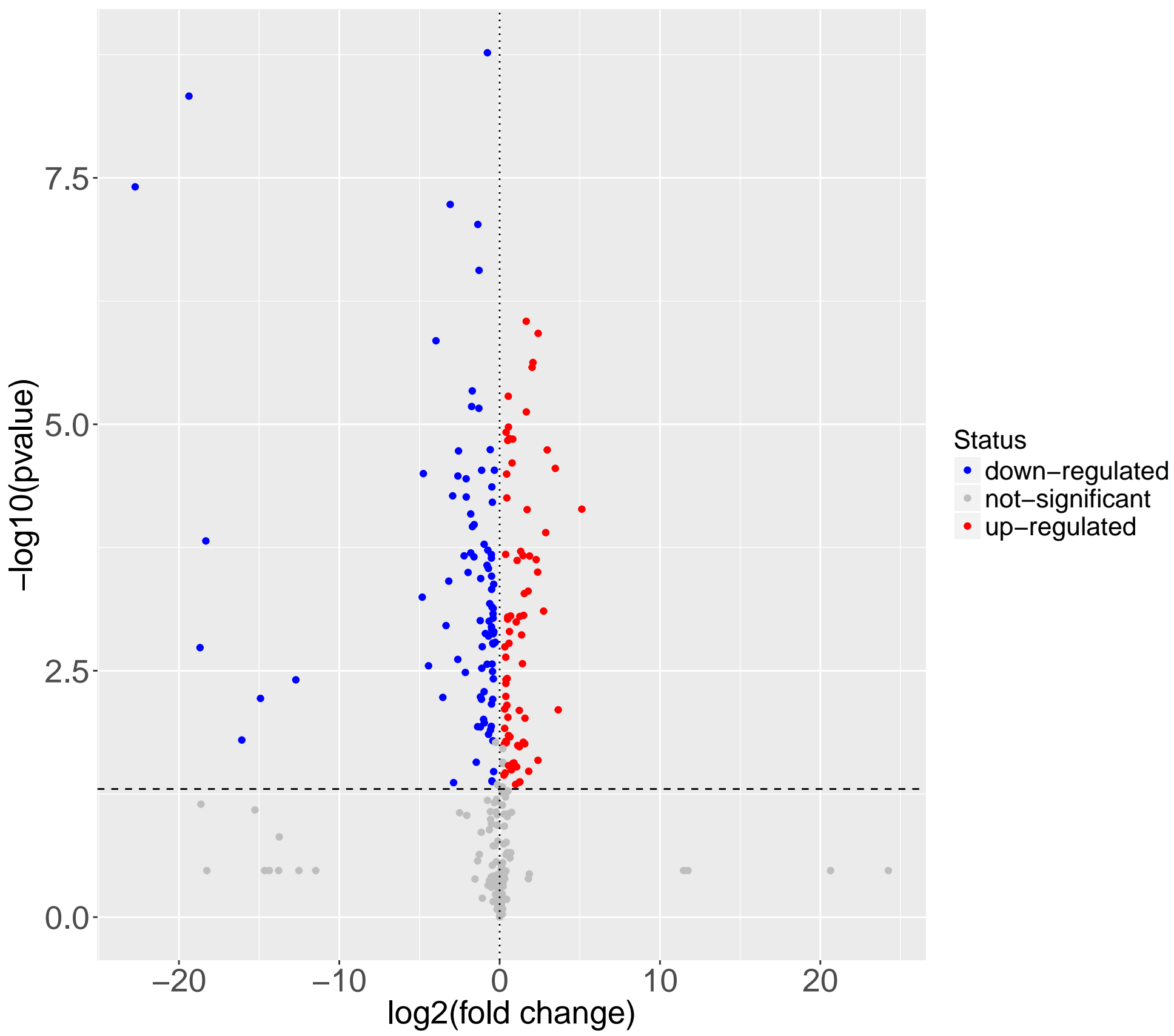

Supplement: Supplementary file 1 [file ijms-20-02330-s001.zip › supplementary material/3、The volcano figure/A0-A4.pdf]

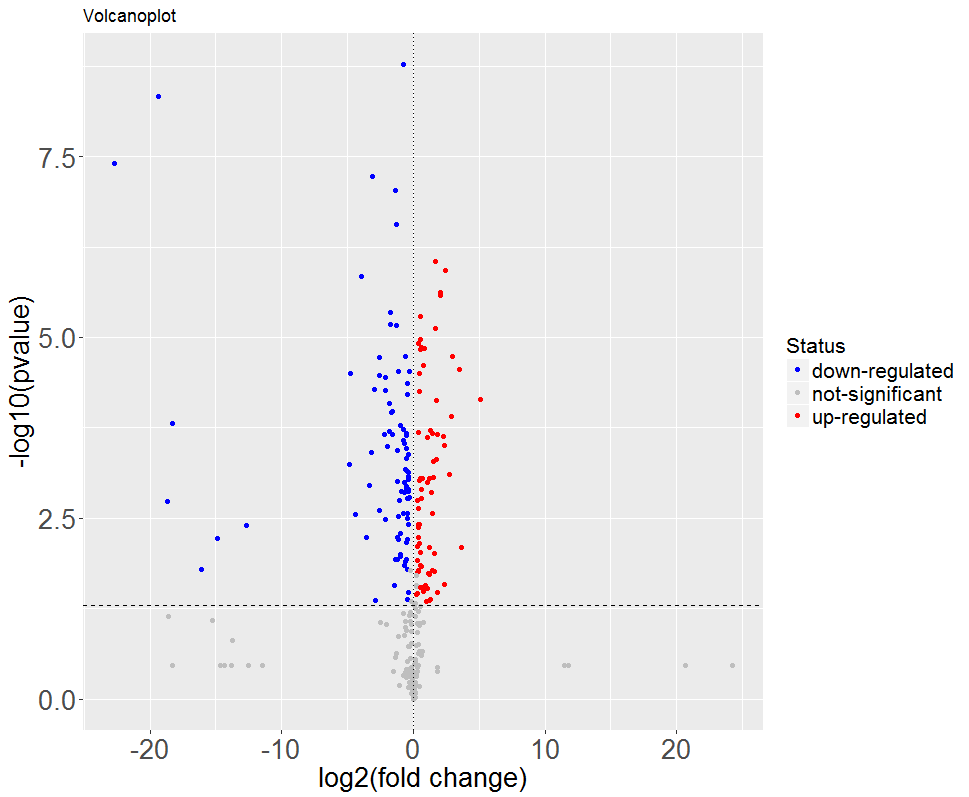

Supplement: Supplementary file 1 [file ijms-20-02330-s001.zip › supplementary material/3、The volcano figure/A0-A4.png]

Volcanoplot

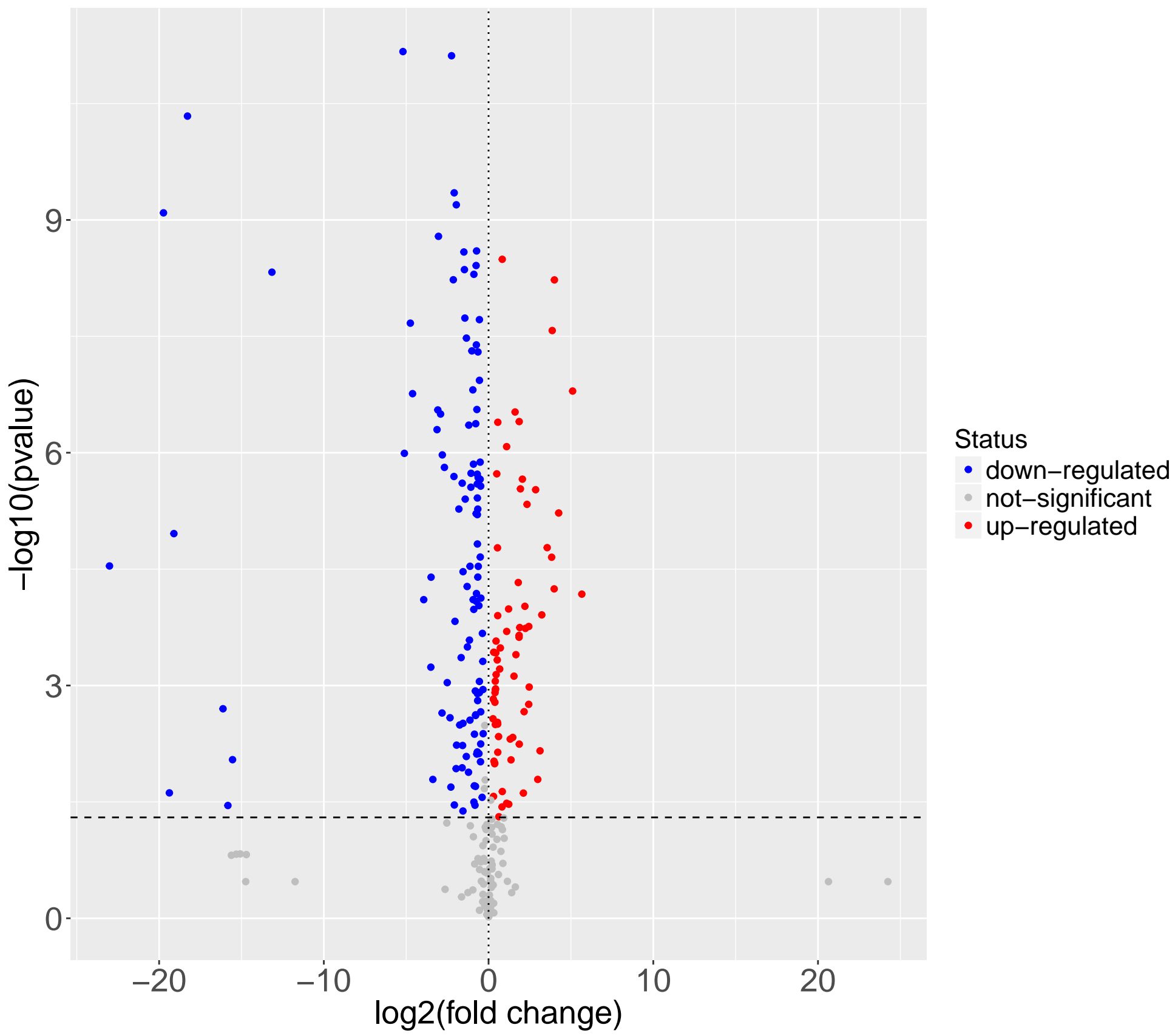

Supplement: Supplementary file 1 [file ijms-20-02330-s001.zip › supplementary material/3、The volcano figure/A0-A8.pdf]

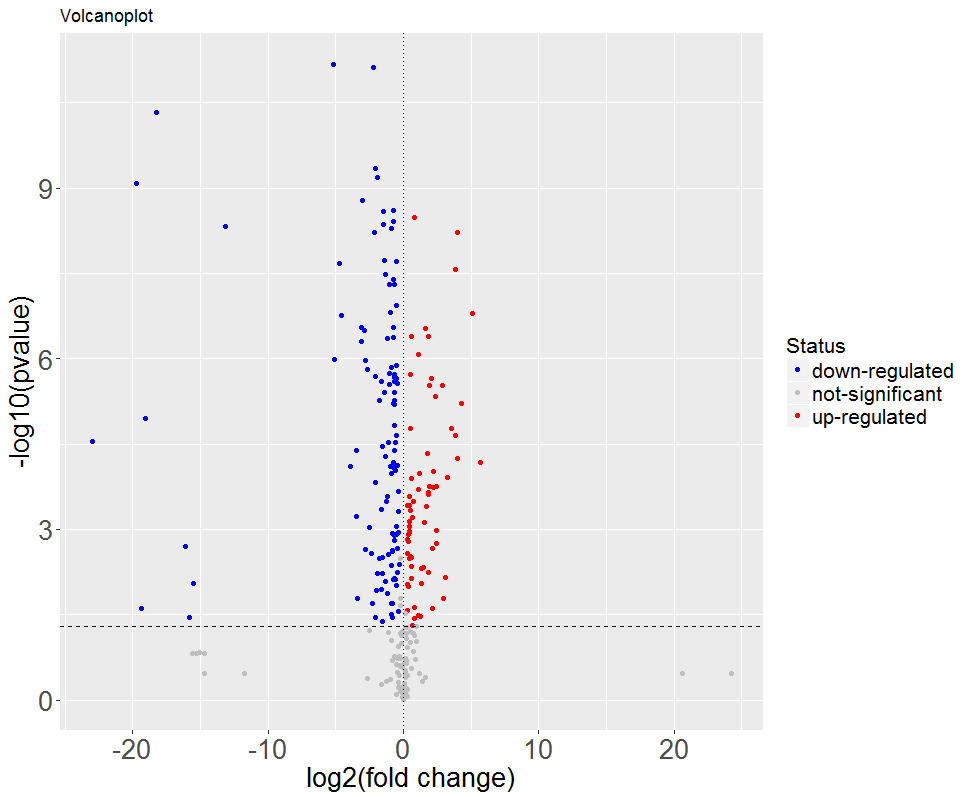

Supplement: Supplementary file 1 [file ijms-20-02330-s001.zip › supplementary material/3、The volcano figure/A0-A8.png]

Volcanoplot

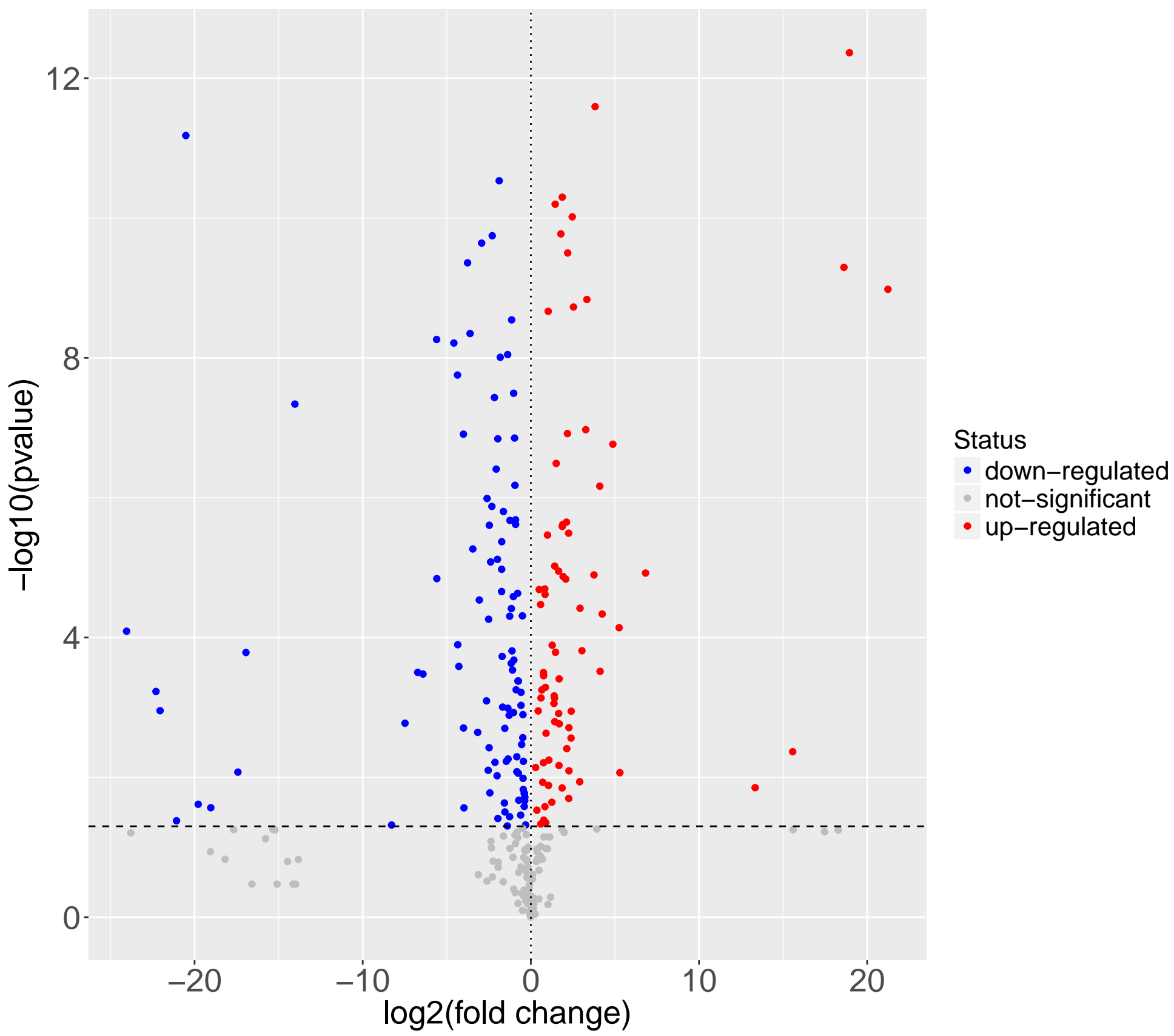

Supplement: Supplementary file 1 [file ijms-20-02330-s001.zip › supplementary material/3、The volcano figure/A0-B0.pdf]

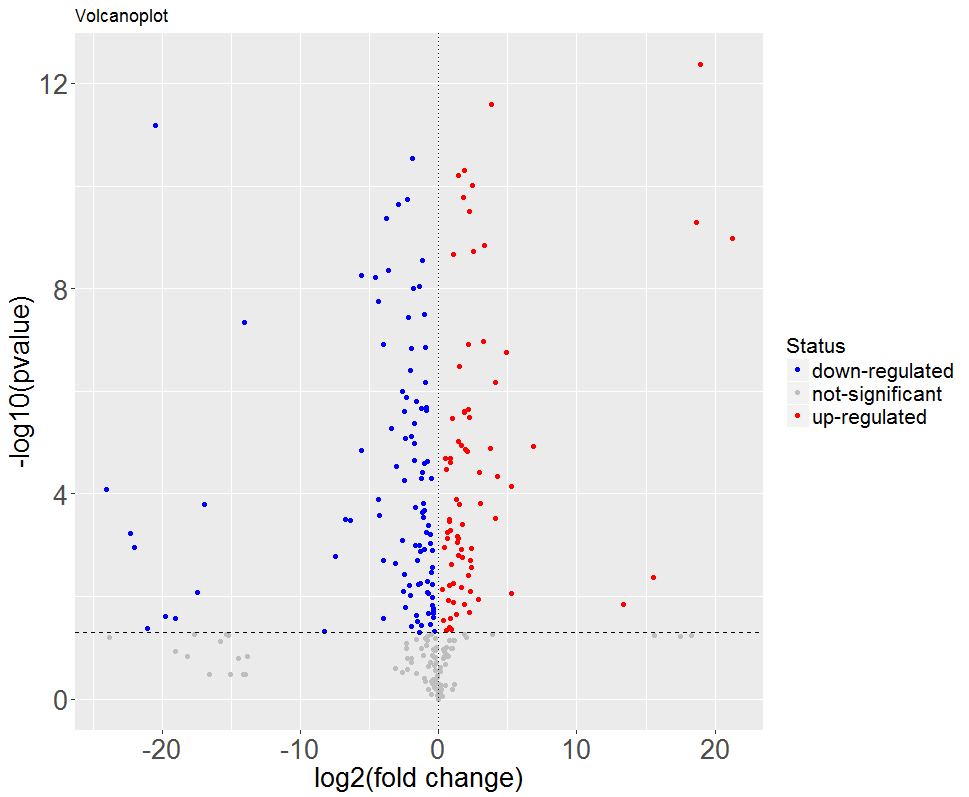

Supplement: Supplementary file 1 [file ijms-20-02330-s001.zip › supplementary material/3、The volcano figure/A0-B0.png]

Volcanoplot

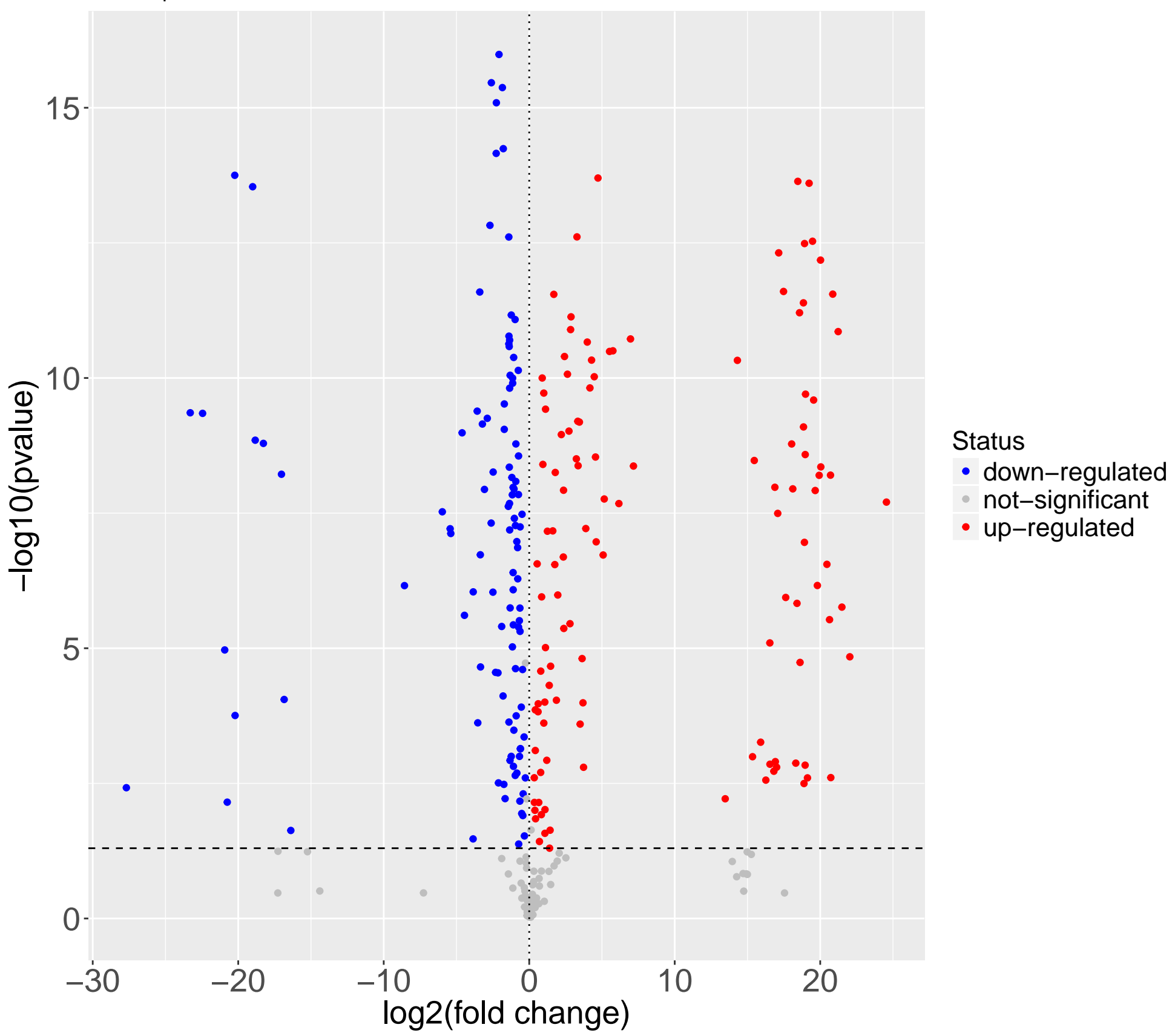

Supplement: Supplementary file 1 [file ijms-20-02330-s001.zip › supplementary material/3、The volcano figure/A12-B12.pdf]

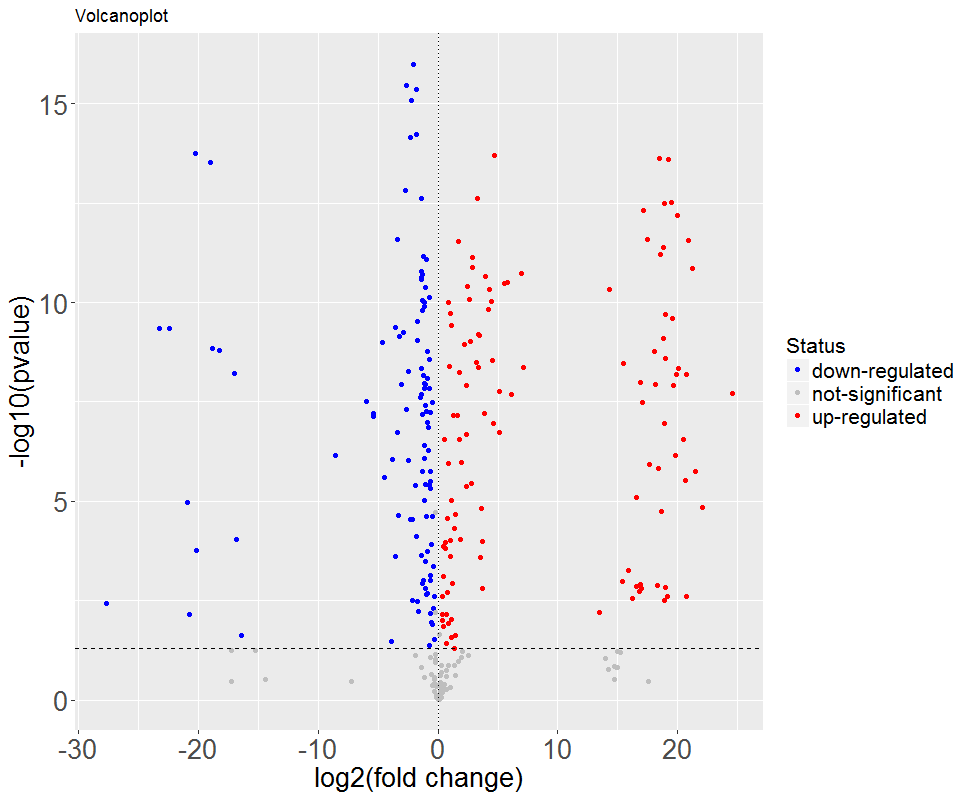

Supplement: Supplementary file 1 [file ijms-20-02330-s001.zip › supplementary material/3、The volcano figure/A12-B12.png]

Volcanoplot

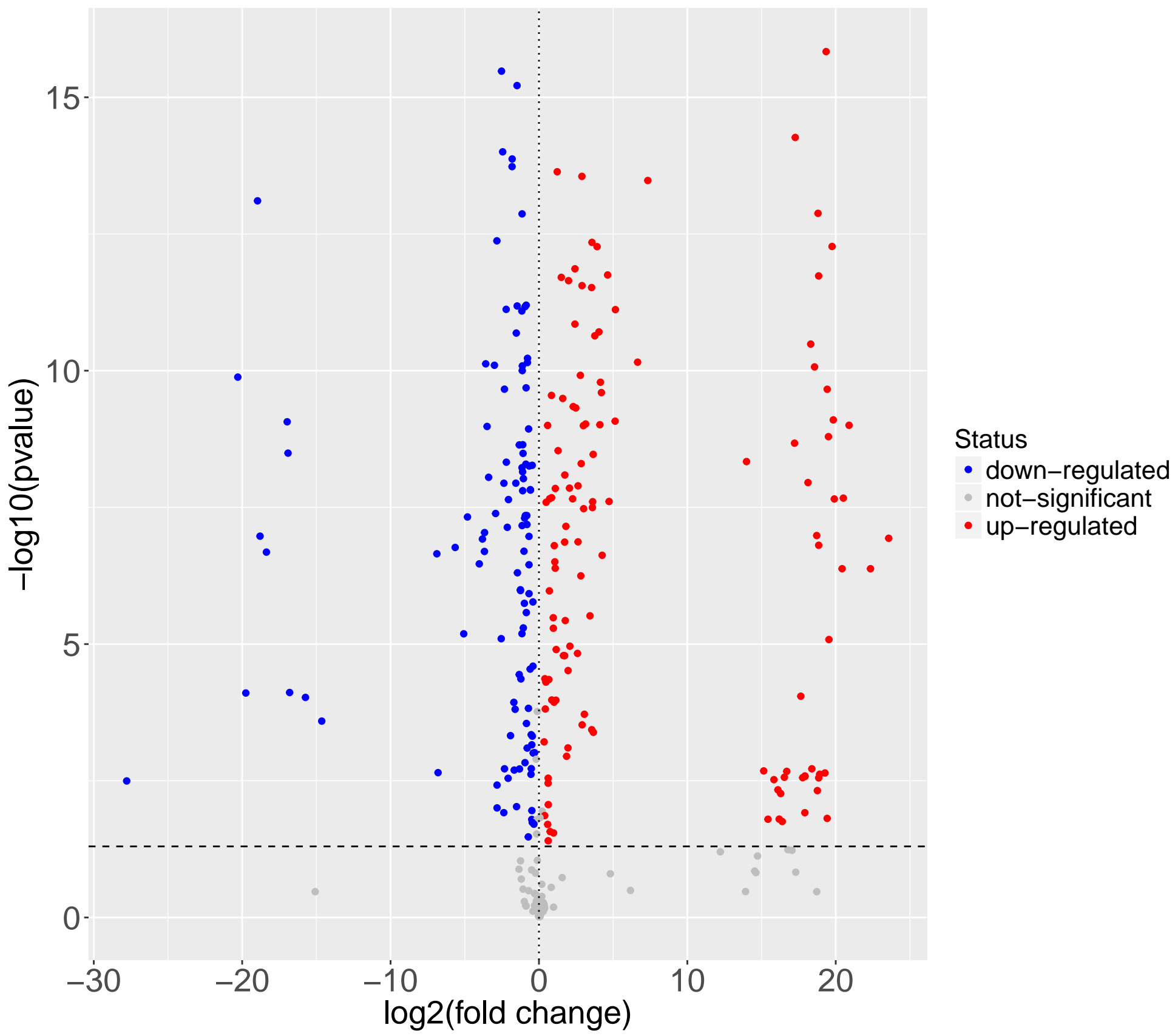

Supplement: Supplementary file 1 [file ijms-20-02330-s001.zip › supplementary material/3、The volcano figure/A18-B18.pdf]

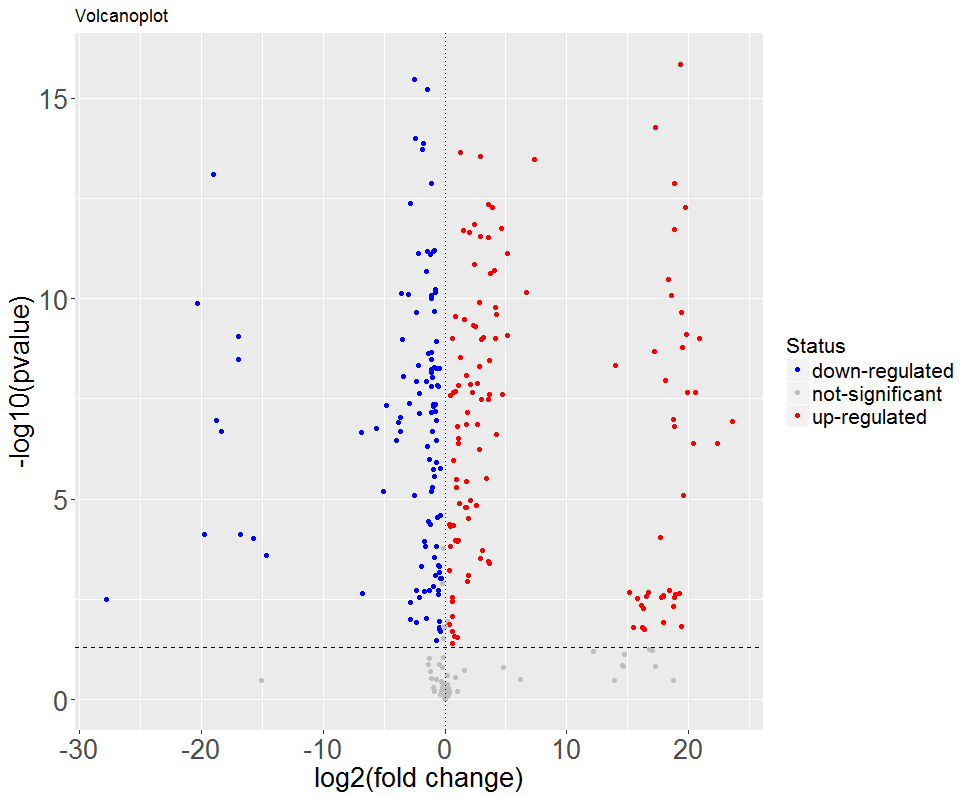

Supplement: Supplementary file 1 [file ijms-20-02330-s001.zip › supplementary material/3、The volcano figure/A18-B18.png]

Volcanoplot

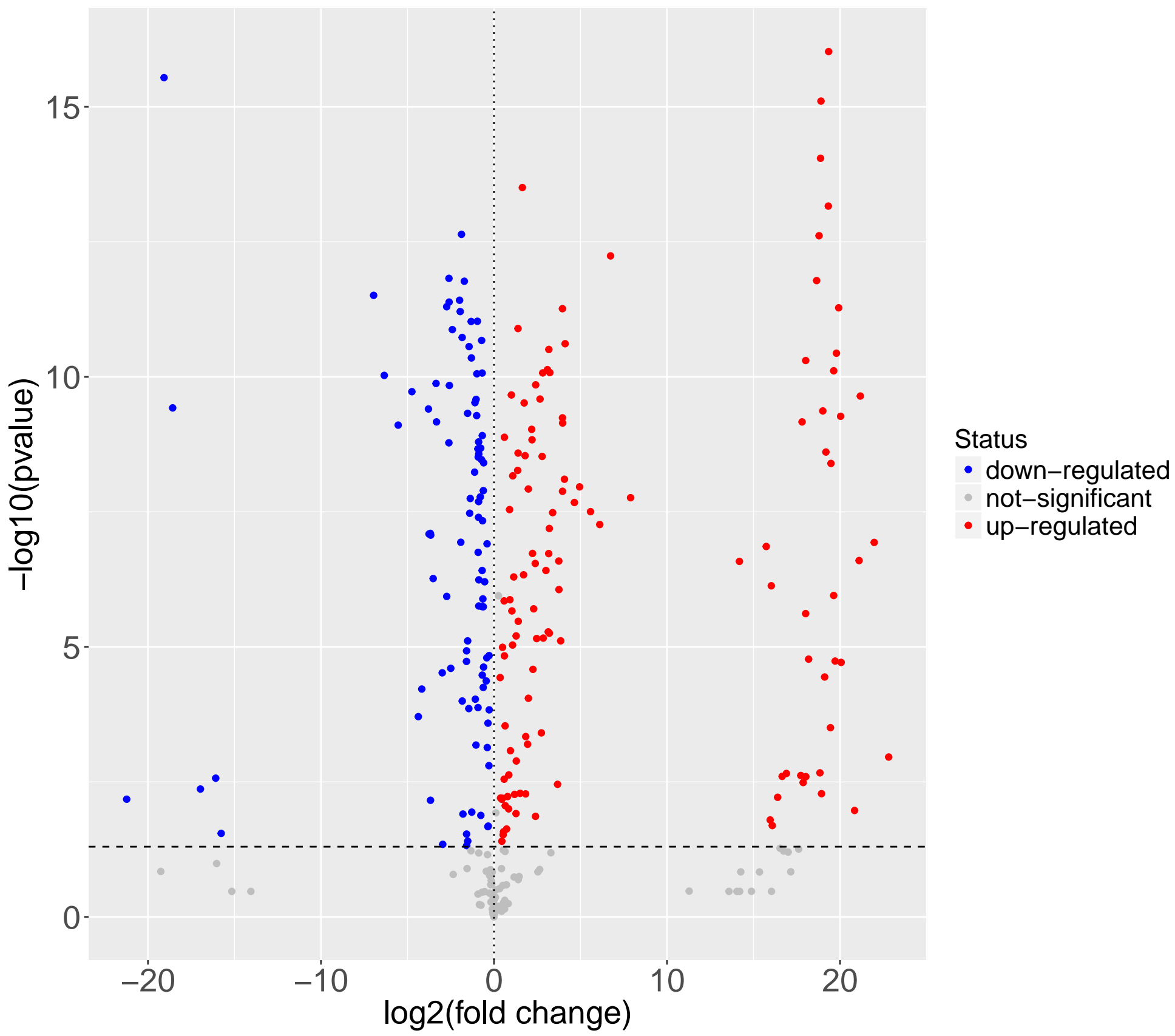

Supplement: Supplementary file 1 [file ijms-20-02330-s001.zip › supplementary material/3、The volcano figure/A24-B24.pdf]

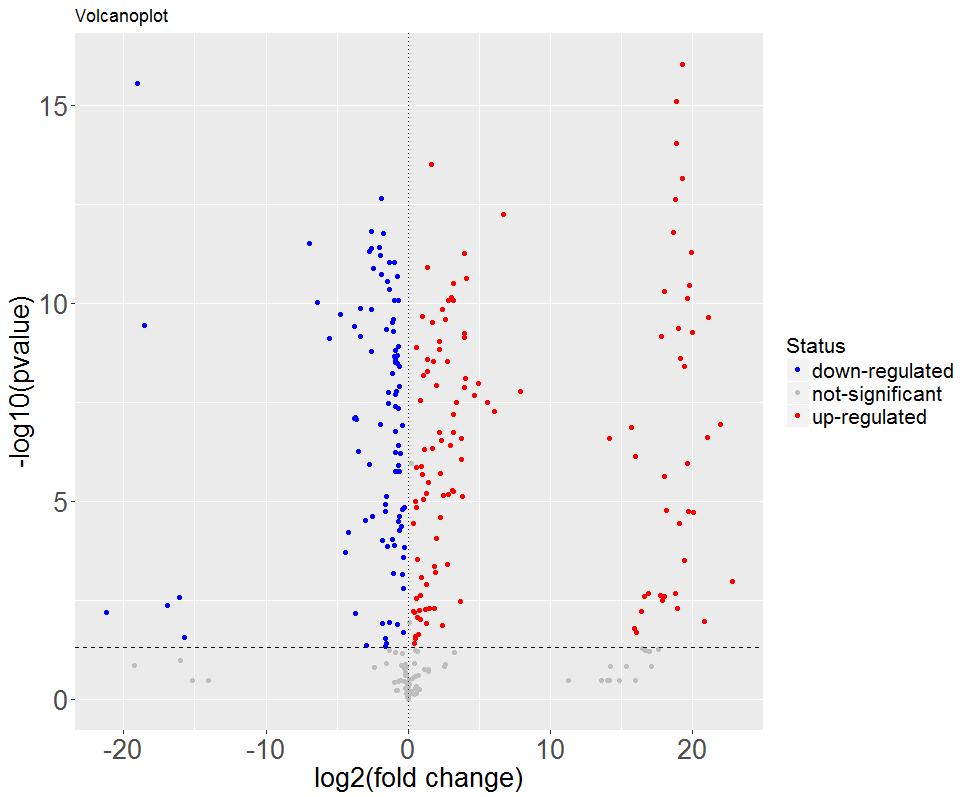

Supplement: Supplementary file 1 [file ijms-20-02330-s001.zip › supplementary material/3、The volcano figure/A24-B24.png]

Volcanoplot

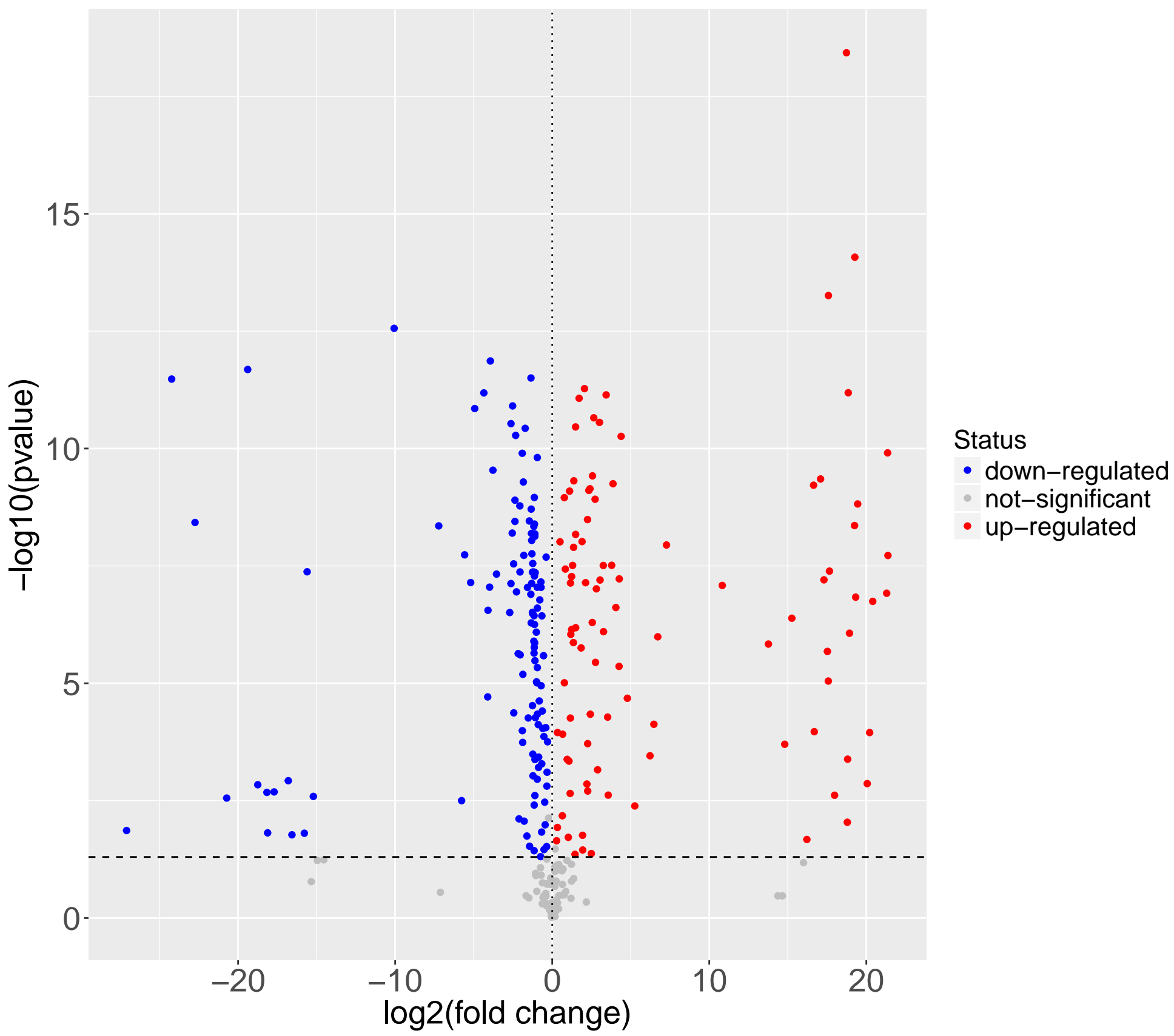

Supplement: Supplementary file 1 [file ijms-20-02330-s001.zip › supplementary material/3、The volcano figure/A4-B4.pdf]

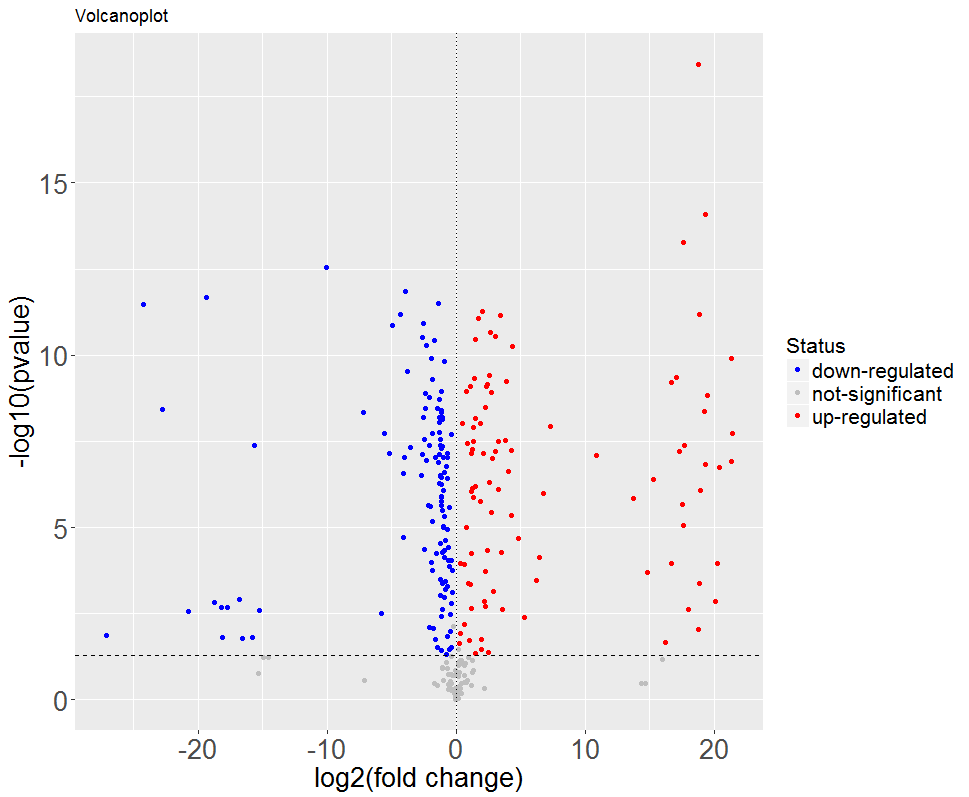

Supplement: Supplementary file 1 [file ijms-20-02330-s001.zip › supplementary material/3、The volcano figure/A4-B4.png]

Volcanoplot

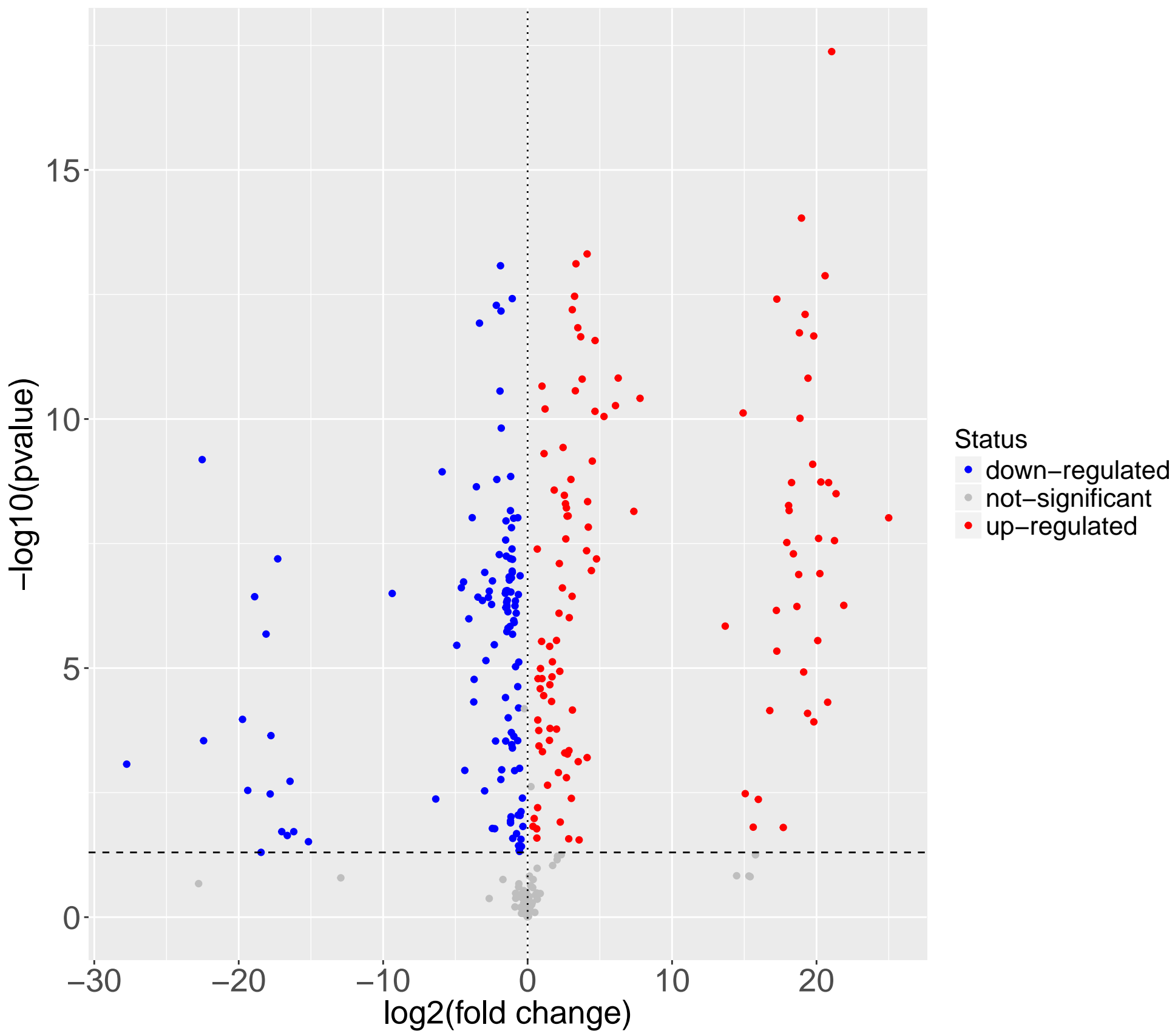

Supplement: Supplementary file 1 [file ijms-20-02330-s001.zip › supplementary material/3、The volcano figure/A8-B8.pdf]

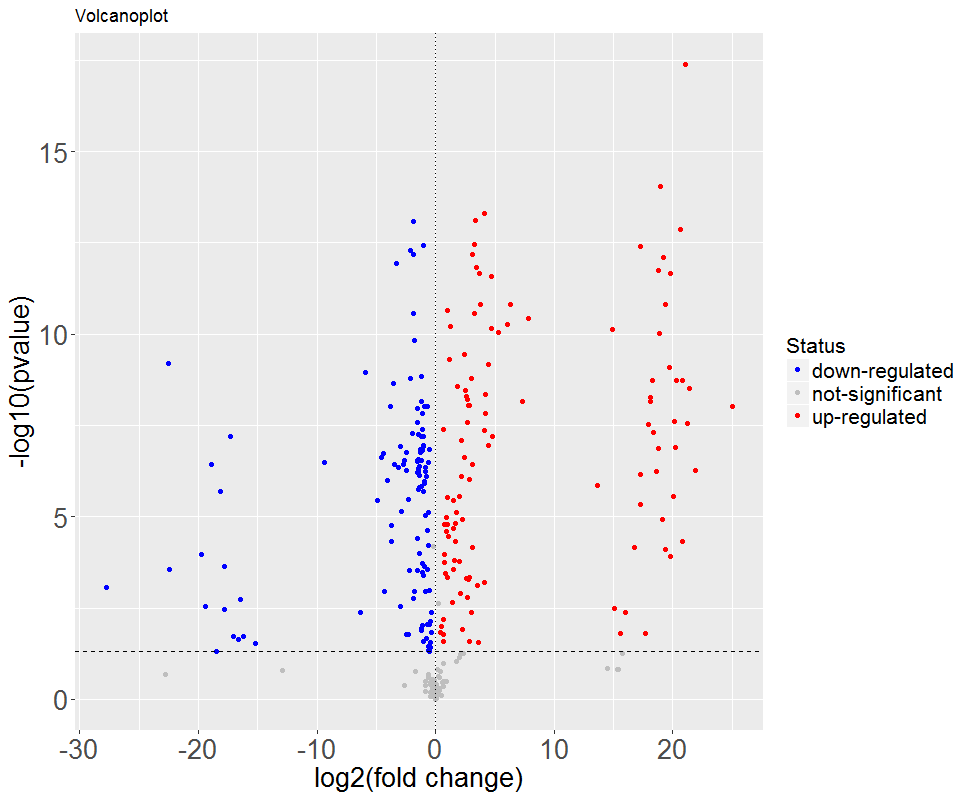

Supplement: Supplementary file 1 [file ijms-20-02330-s001.zip › supplementary material/3、The volcano figure/A8-B8.png]

Volcanoplot

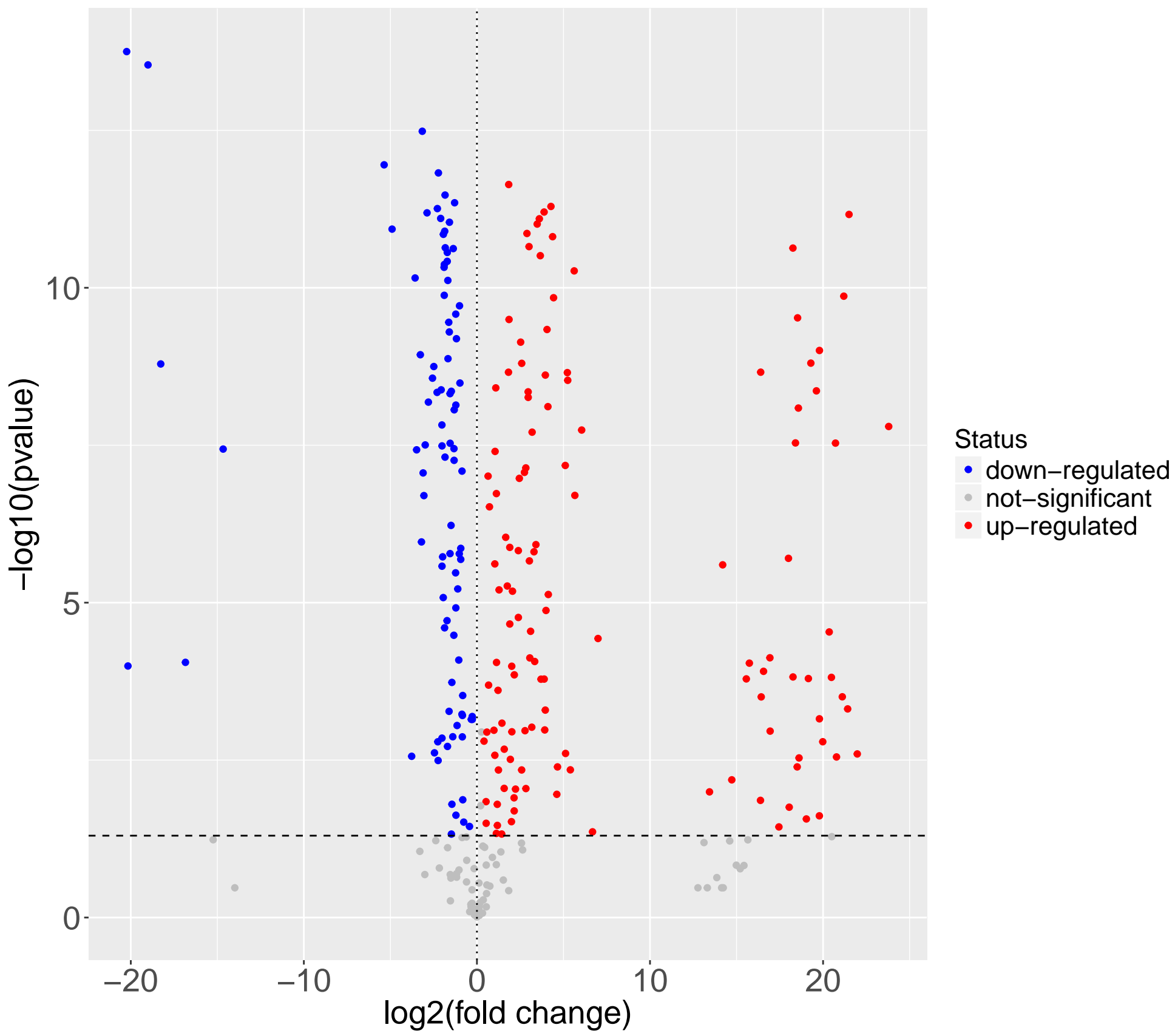

Supplement: Supplementary file 1 [file ijms-20-02330-s001.zip › supplementary material/3、The volcano figure/B0-B12.pdf]

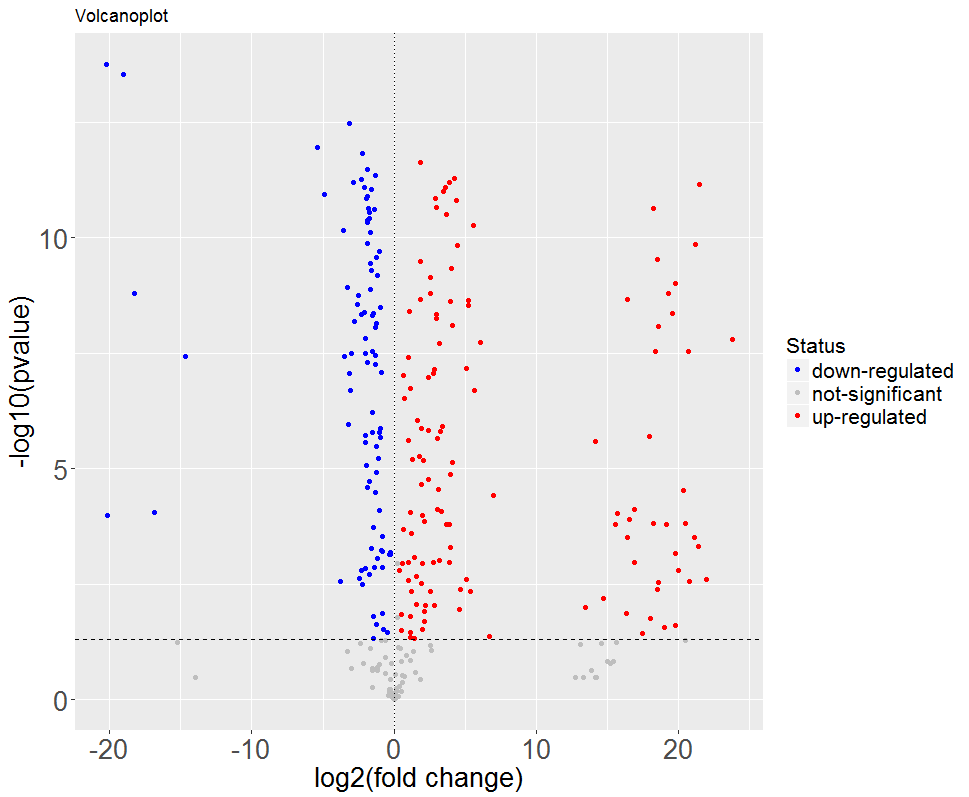

Supplement: Supplementary file 1 [file ijms-20-02330-s001.zip › supplementary material/3、The volcano figure/B0-B12.png]

Volcanoplot

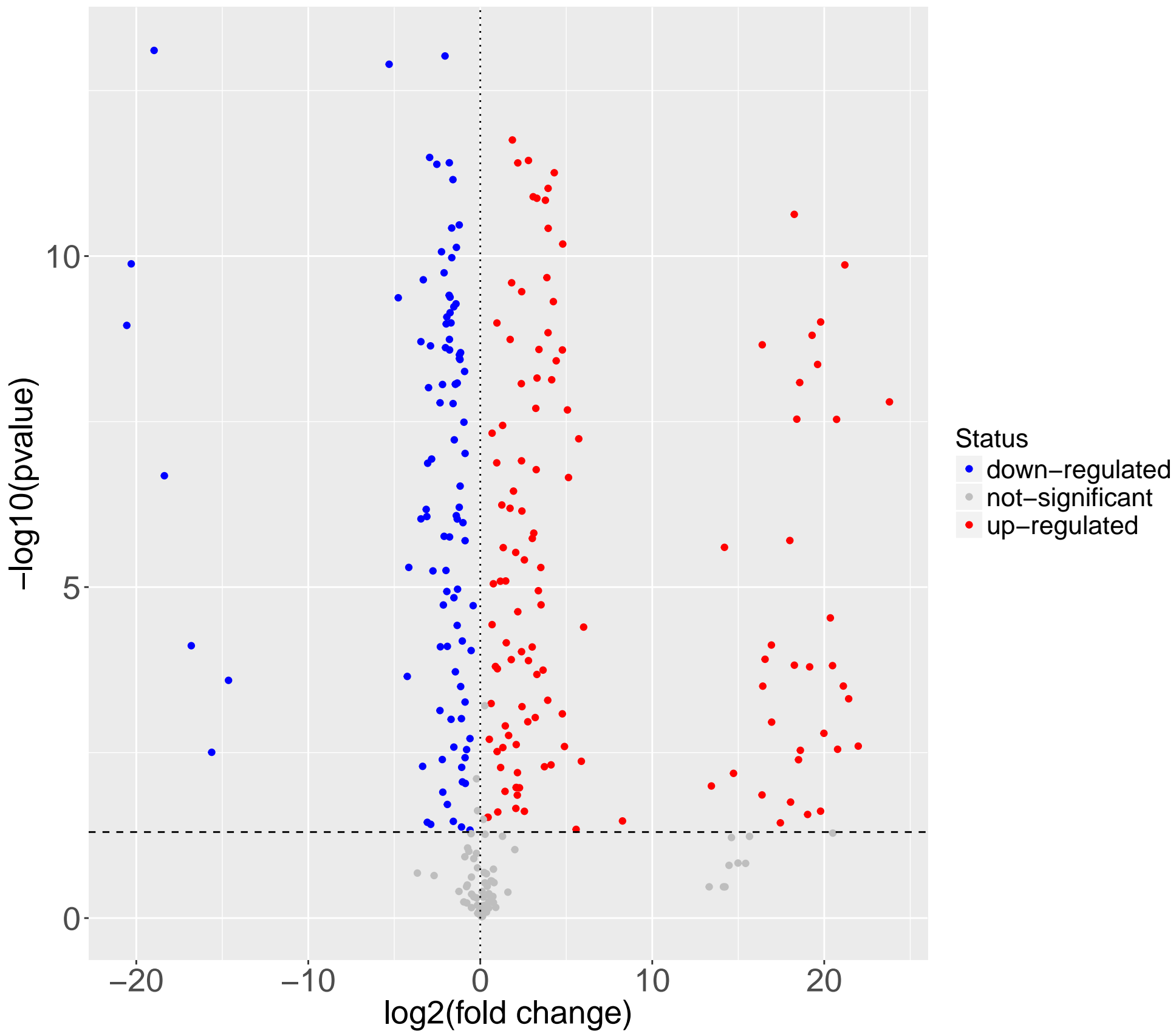

Supplement: Supplementary file 1 [file ijms-20-02330-s001.zip › supplementary material/3、The volcano figure/B0-B18.pdf]

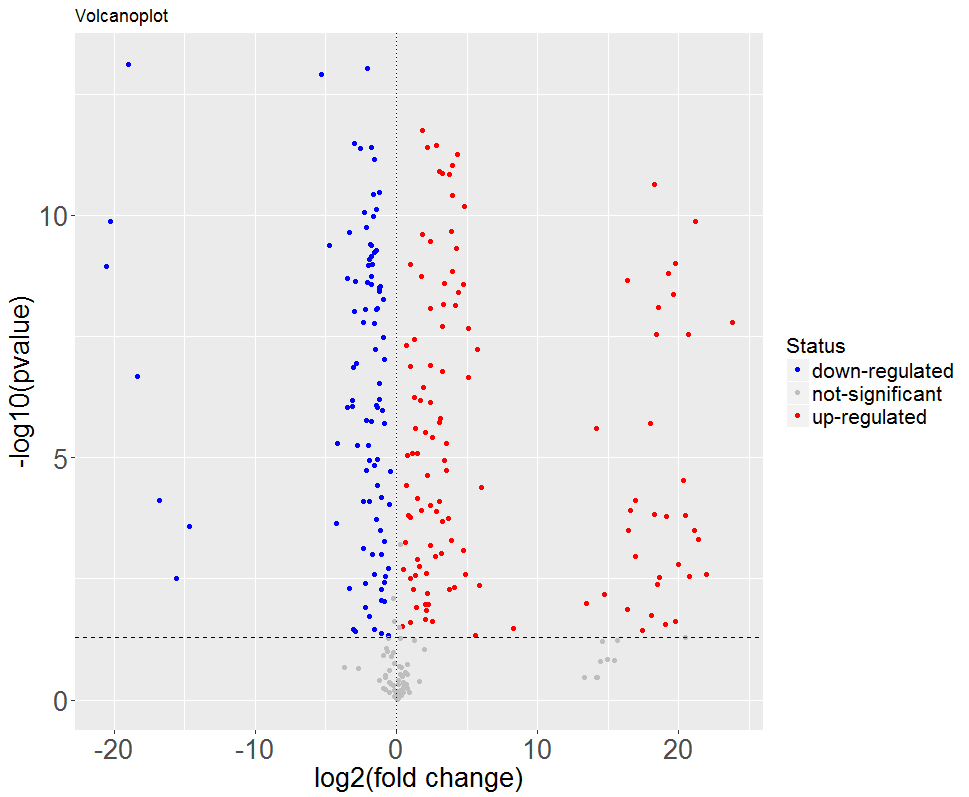

Supplement: Supplementary file 1 [file ijms-20-02330-s001.zip › supplementary material/3、The volcano figure/B0-B18.png]

Volcanoplot

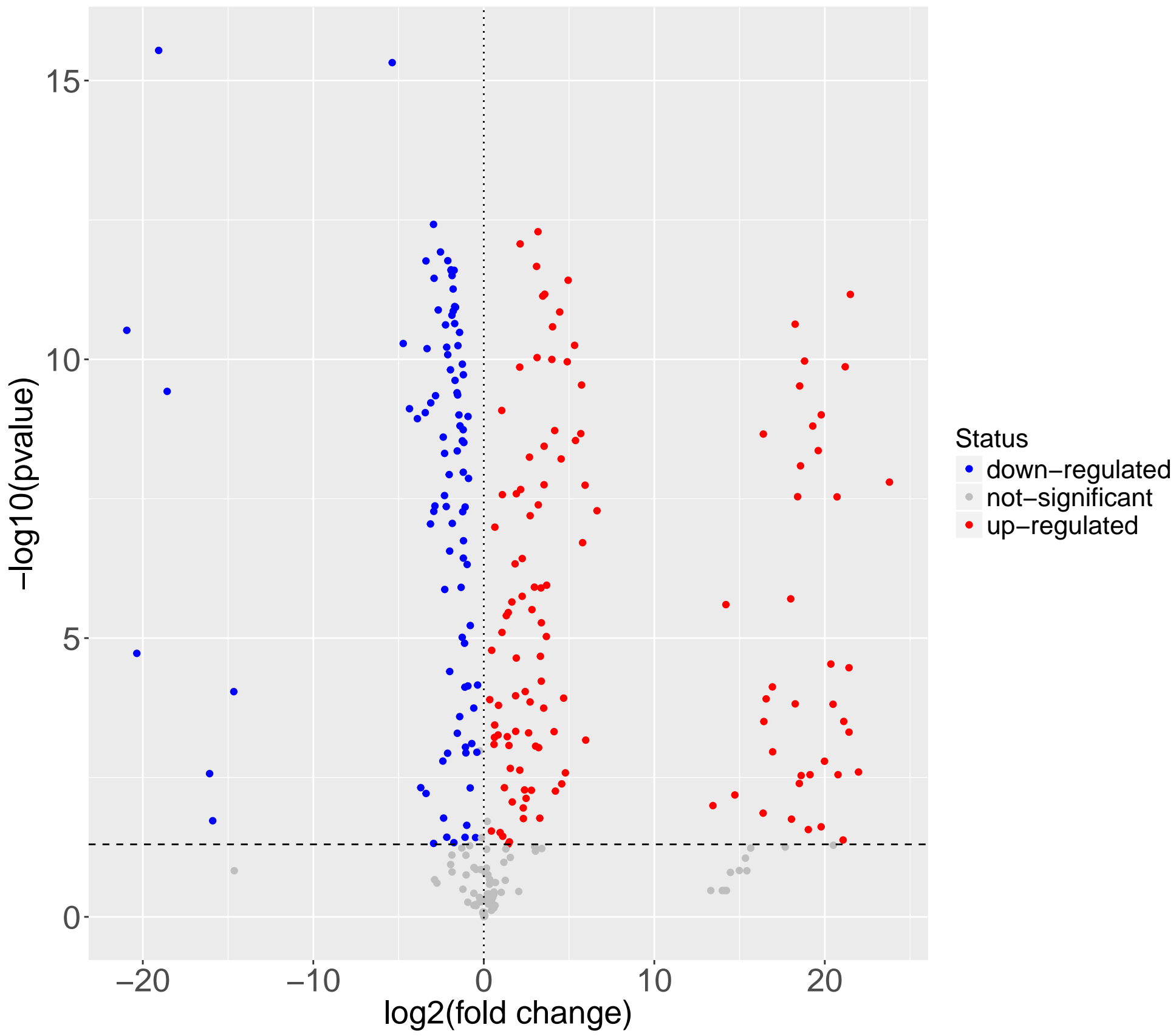

Supplement: Supplementary file 1 [file ijms-20-02330-s001.zip › supplementary material/3、The volcano figure/B0-B24.pdf]

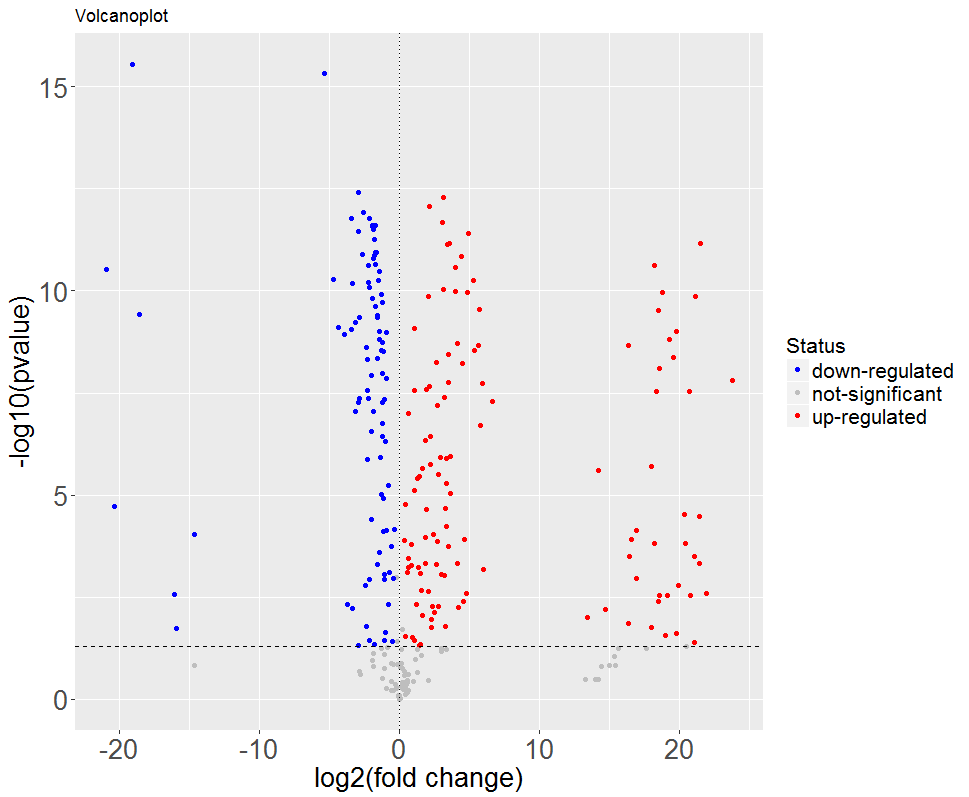

Supplement: Supplementary file 1 [file ijms-20-02330-s001.zip › supplementary material/3、The volcano figure/B0-B24.png]

Volcanoplot

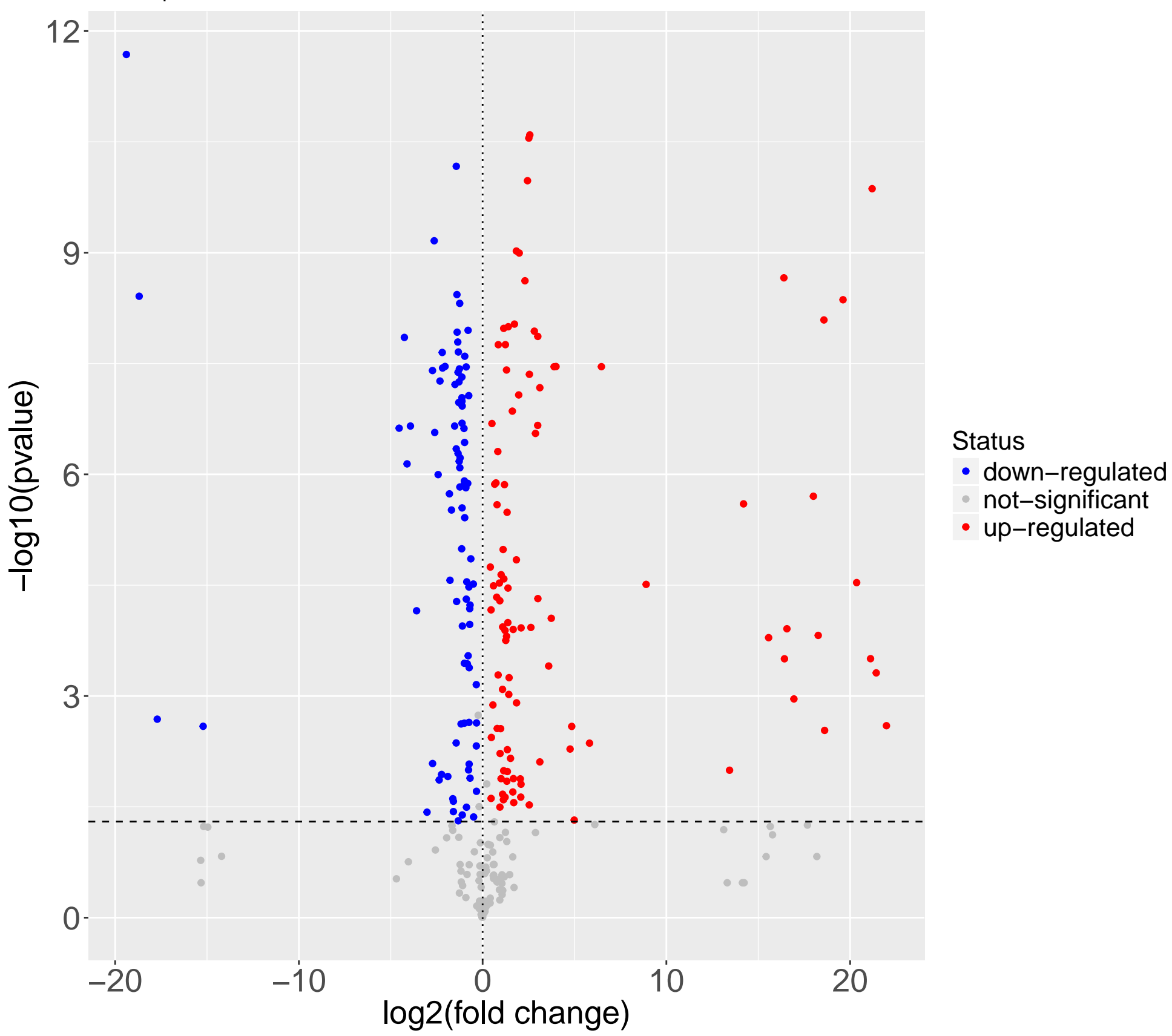

Supplement: Supplementary file 1 [file ijms-20-02330-s001.zip › supplementary material/3、The volcano figure/B0-B4.pdf]

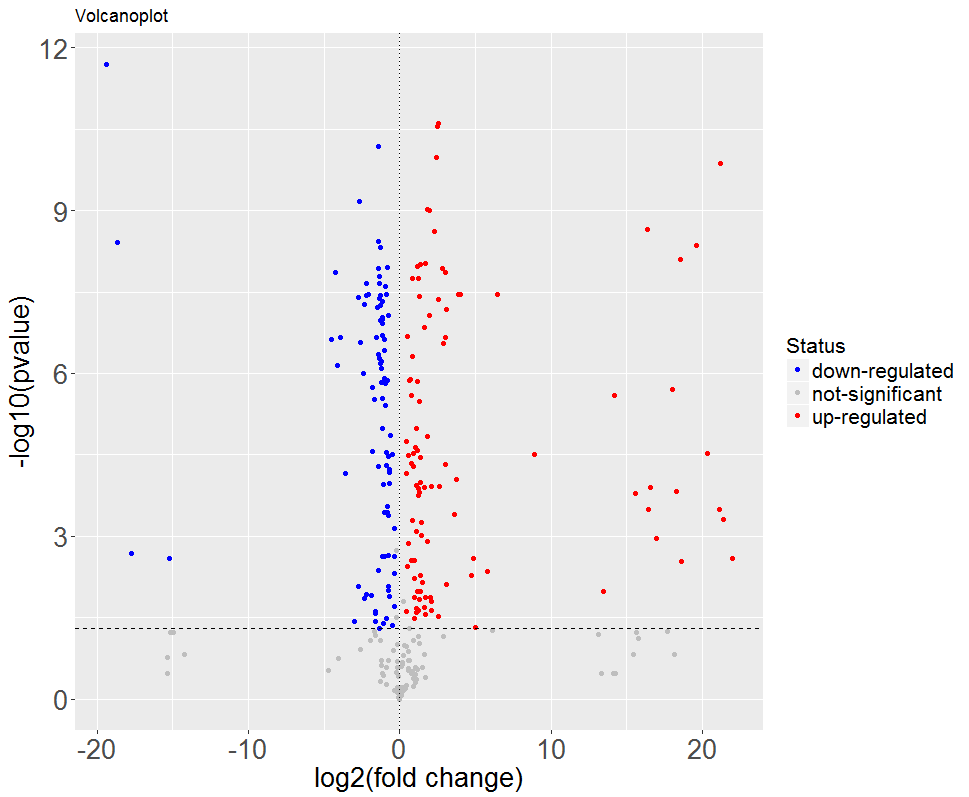

Supplement: Supplementary file 1 [file ijms-20-02330-s001.zip › supplementary material/3、The volcano figure/B0-B4.png]

Volcanoplot

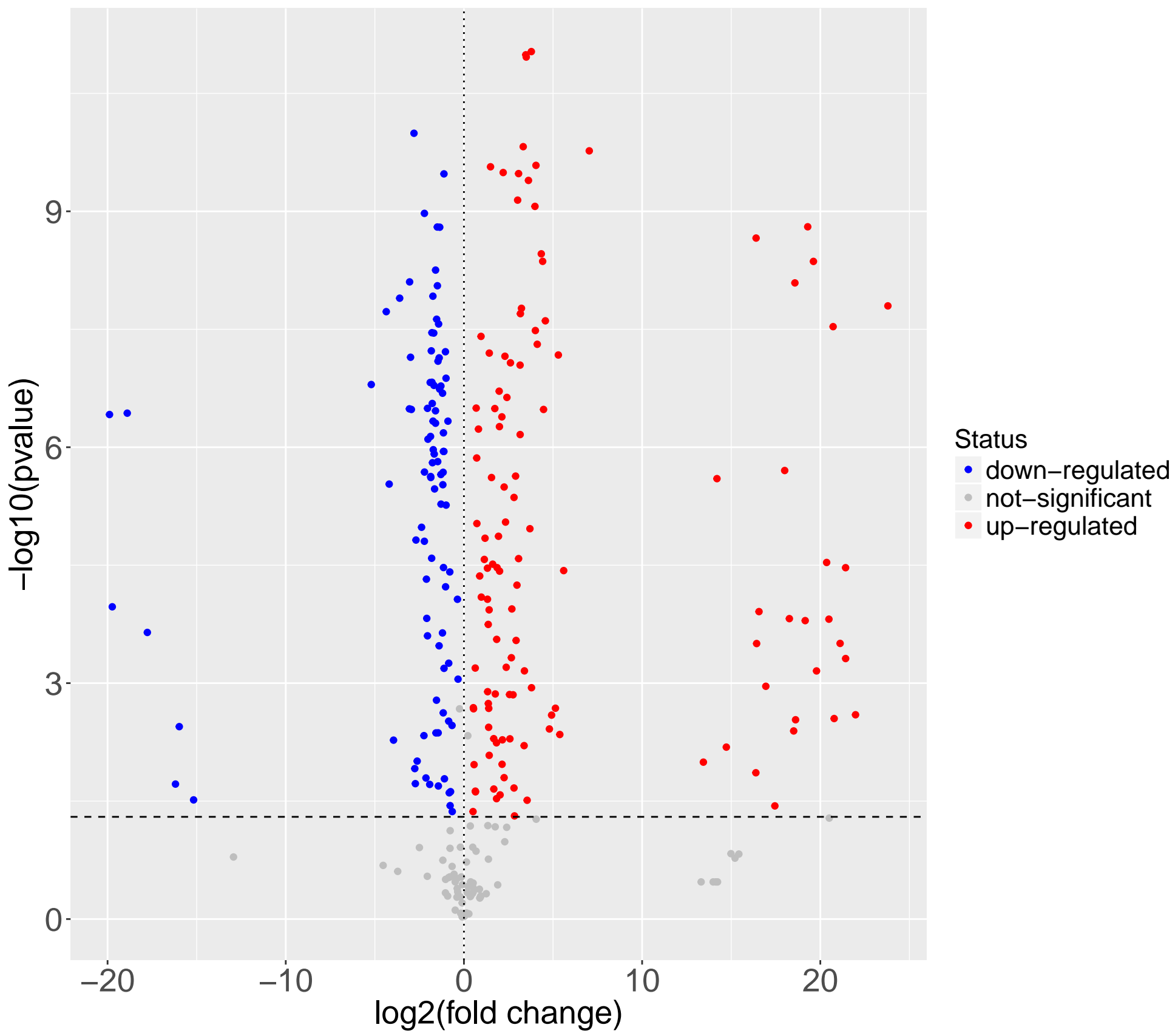

Supplement: Supplementary file 1 [file ijms-20-02330-s001.zip › supplementary material/3、The volcano figure/B0-B8.pdf]

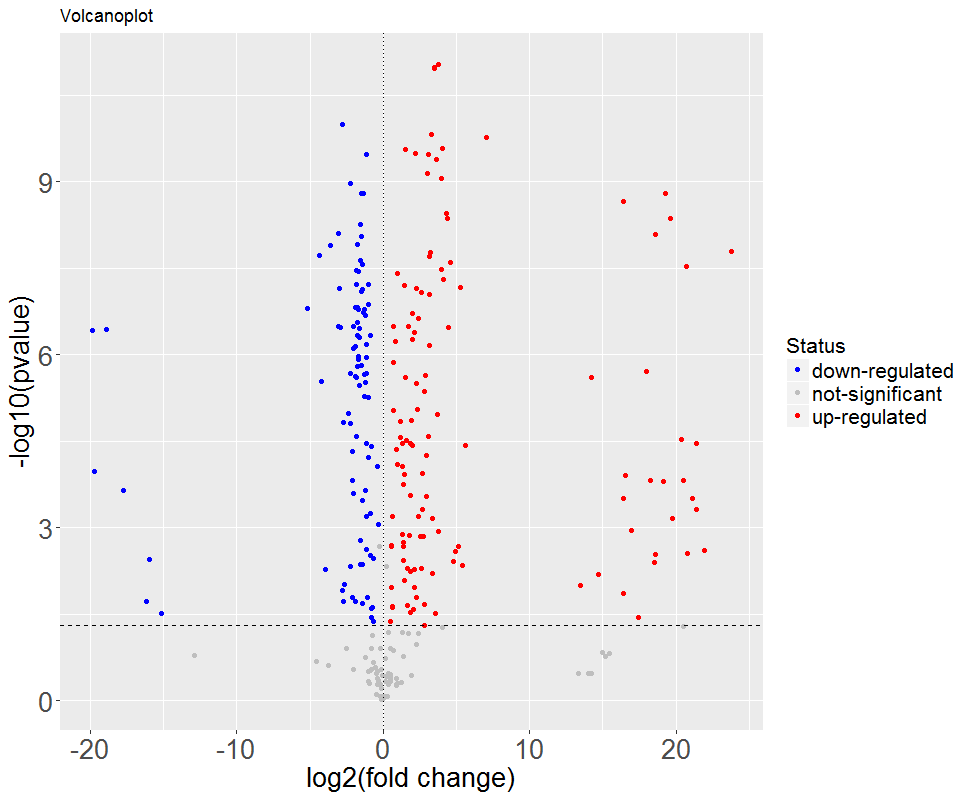

Supplement: Supplementary file 1 [file ijms-20-02330-s001.zip › supplementary material/3、The volcano figure/B0-B8.png]
